# Supplementary material for: Derivatization of Natural Compound β-Pinene Enhances Its In Vitro Antifungal Activity against Plant Pathogens
Source: Molecules. 2019 Aug 29;24(17):3144. doi: 10.3390/molecules24173144 (PMC6749435; doi:10.3390/molecules24173144)

IR Spectra of 3 and 4a-4t

(1S,2S,5S)-6,6-dimethylbicyclo[3.1.1]heptane-2-carboxylic acid (3)

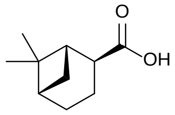

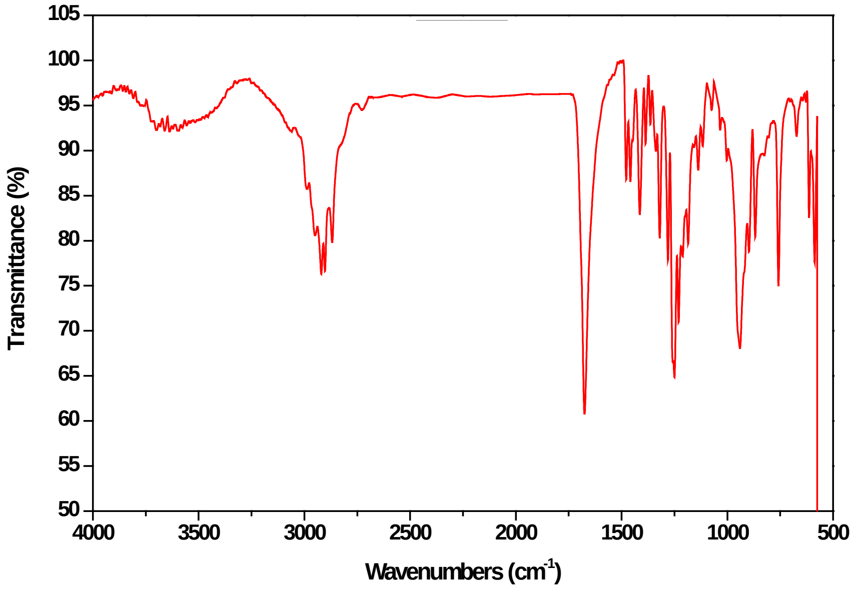

(1S,2S,5S)-6,6-dimethyl-N-phenylbicyclo[3.1.1]heptane-2-carboxamide (4a)

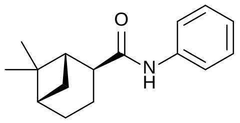

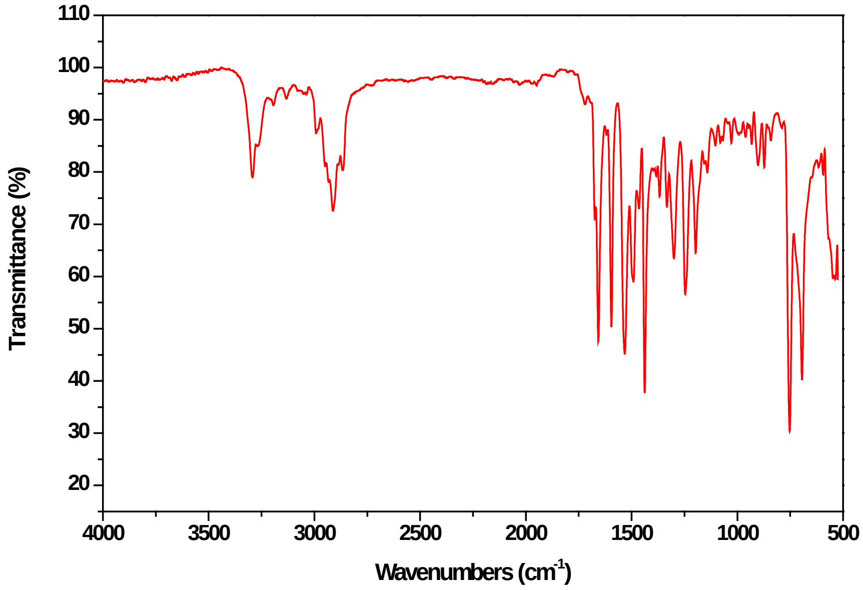

(1S,2S,5S)-N-(2-bromophenyl)-6,6-dimethylbicyclo[3.1.1]heptane-2-carboxamide (4b)

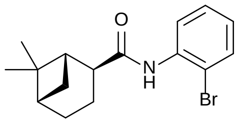

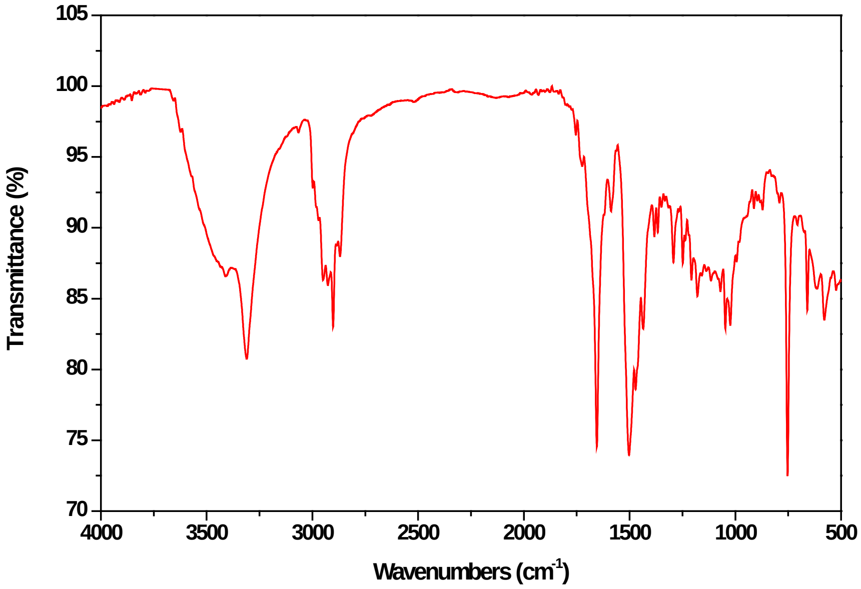

(1S,2S,5S)-N-(3-bromophenyl)-6,6-dimethylbicyclo[3.1.1]heptane-2-carboxamide (4c)

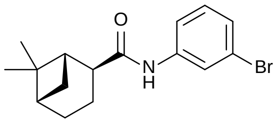

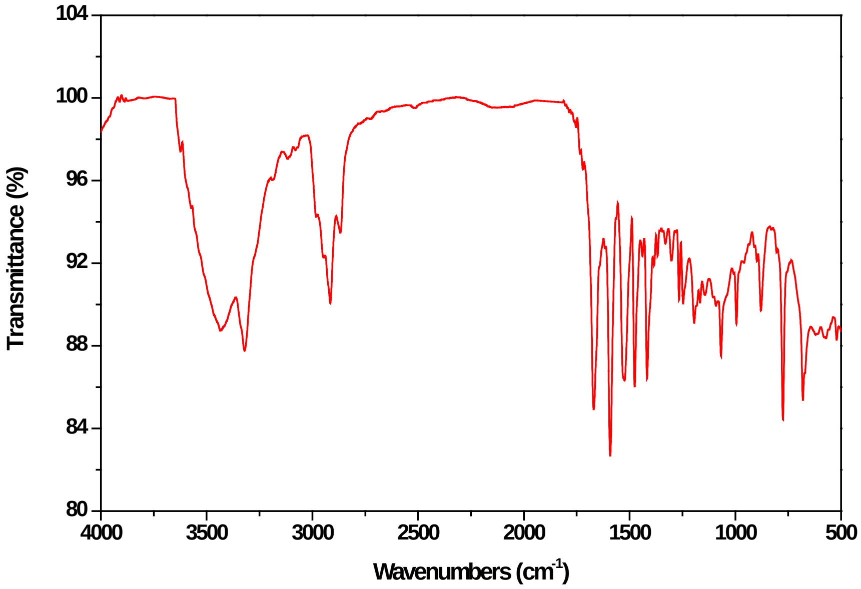

(1S,2S,5S)-N-(4-ethylphenyl)-6,6-dimethylbicyclo[3.1.1]heptane-2-carboxamide (4d)

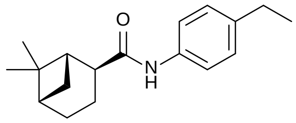

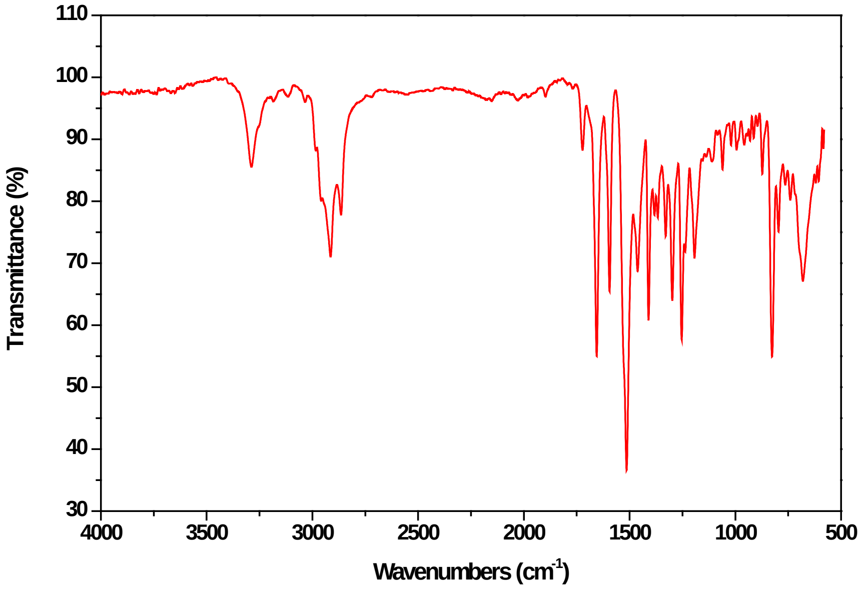

(1S,2S,5S)-6,6-dimethyl-N-(4-(trifluoromethyl)phenyl)bicyclo[3.1.1]heptane-2-carboxamide (4e)

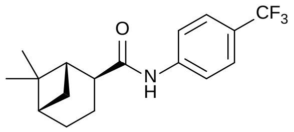

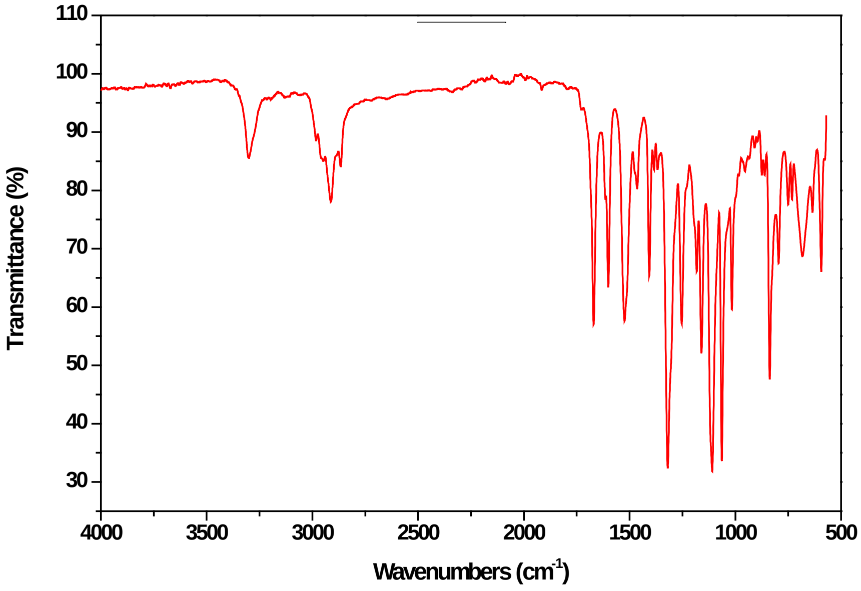

(1S,2S,5S)-N-(2,6-difluorophenyl)-6,6-dimethylbicyclo[3.1.1]heptane-2-carboxamide (4f)

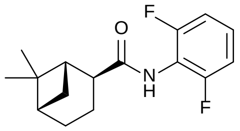

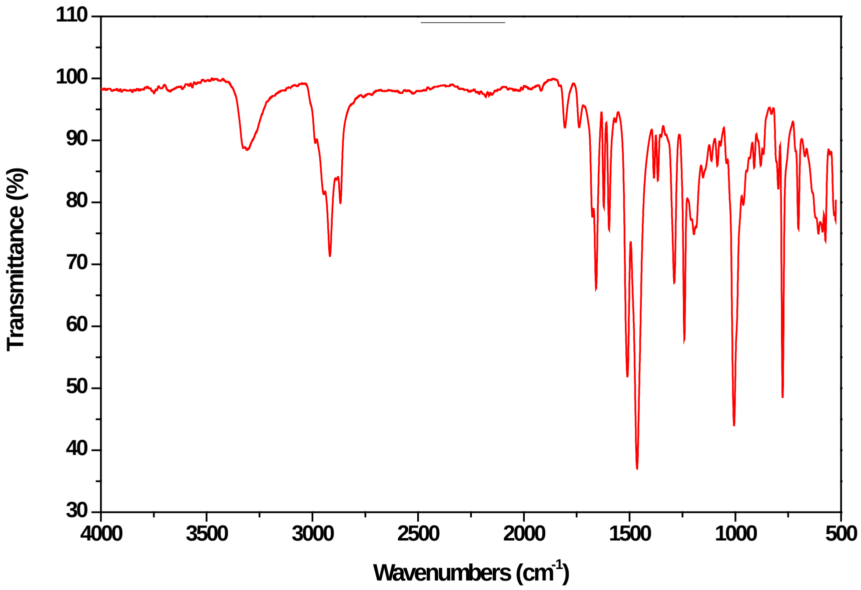

(1S,2S,5S)-N-(4-fluorophenyl)-6,6-dimethylbicyclo[3.1.1]heptane-2-carboxamide (4g)

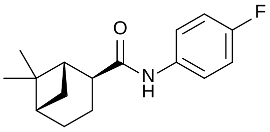

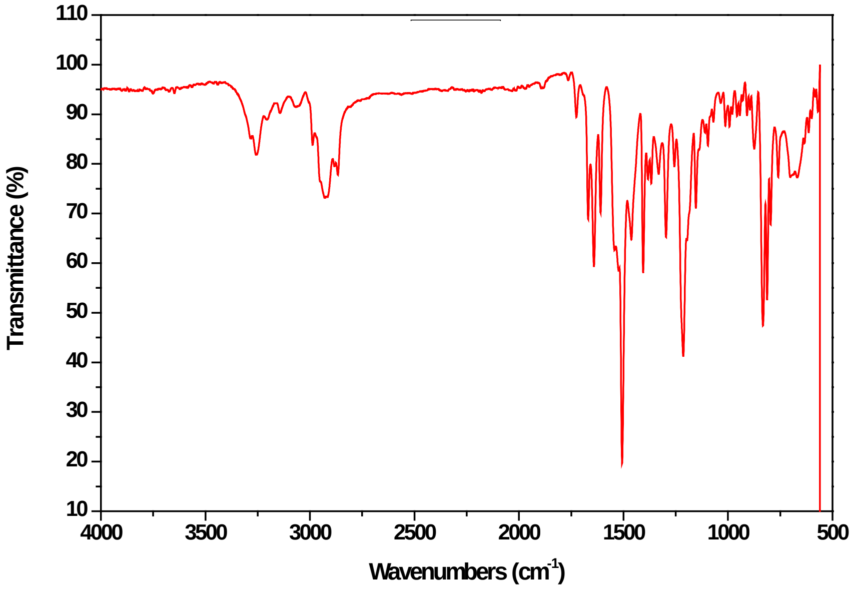

(1S,2S,5S)-6,6-dimethyl-N-(4-nitrophenyl)bicyclo[3.1.1]heptane-2-carboxamide (4h)

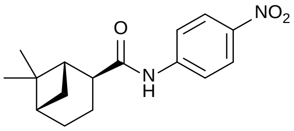

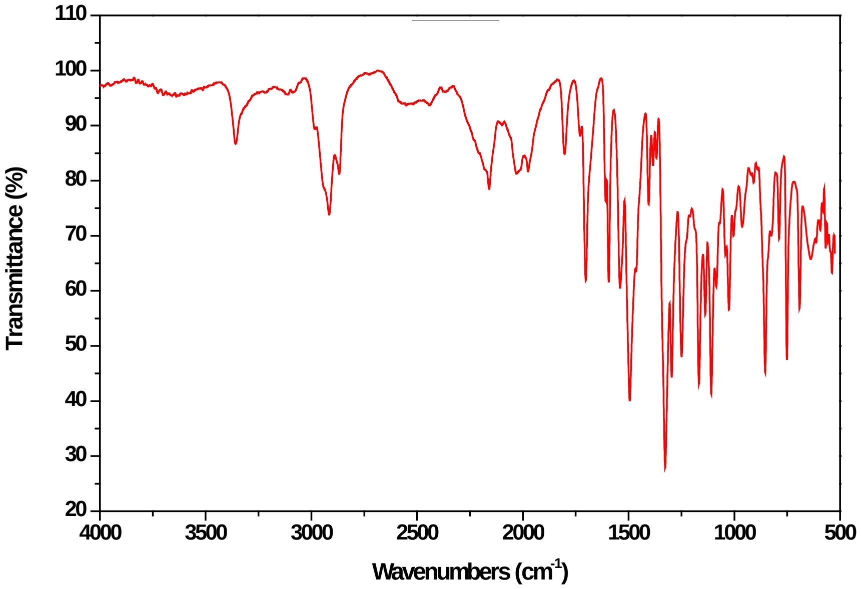

(1S,2S,5S)-6,6-dimethyl-N-(pyridin-2-yl)bicyclo[3.1.1]heptane-2-carboxamide (4i)

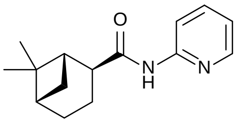

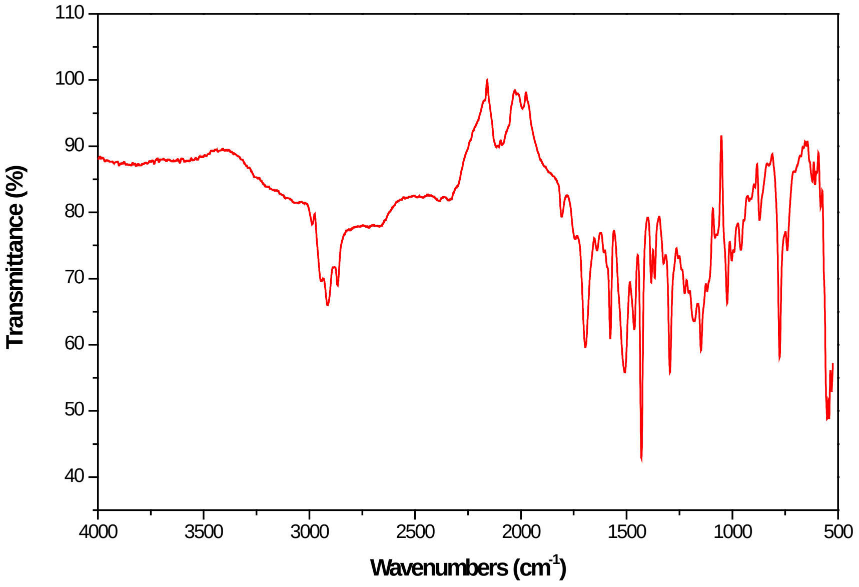

(1S,2S,5S)-N-benzyl-6,6-dimethylbicyclo[3.1.1]heptane-2-carboxamide (4j)

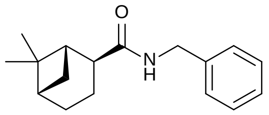

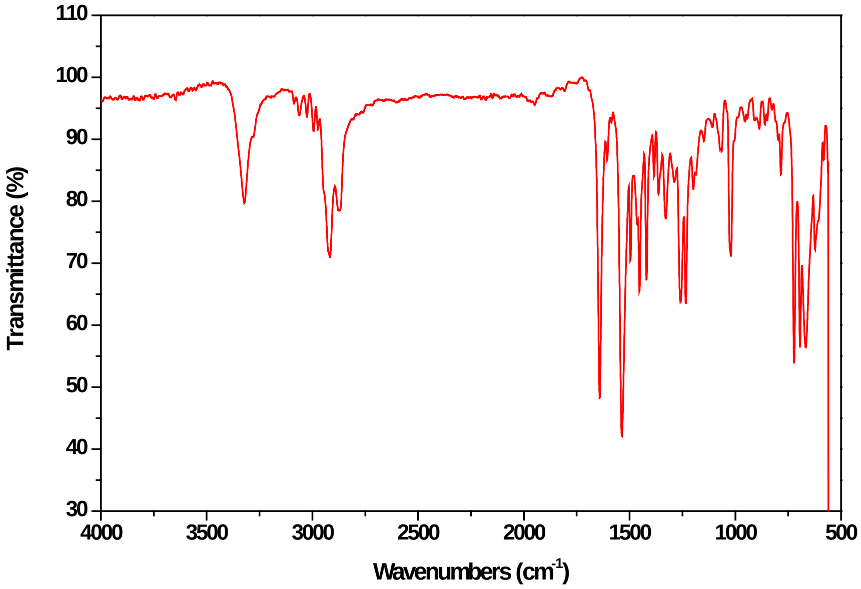

(1S,2S,5S)-N-(4-(4-fluorophenyl)thiazol-2-yl)-6,6-dimethylbicyclo[3.1.1]heptane-2-carboxamide

(4k)

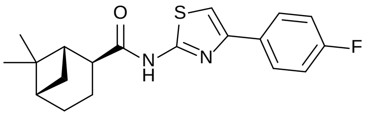

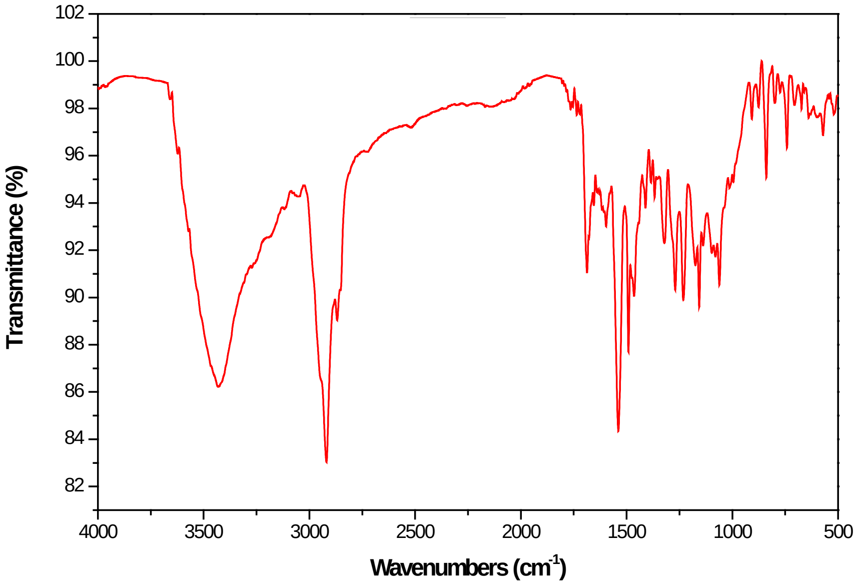

(1S,2S,5S)-N-(4-(4-methoxyphenyl)thiazol-2-yl)-6,6-dimethylbicyclo[3.1.1]heptane-2-carboxamide (4l)

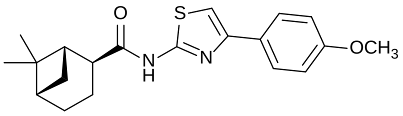

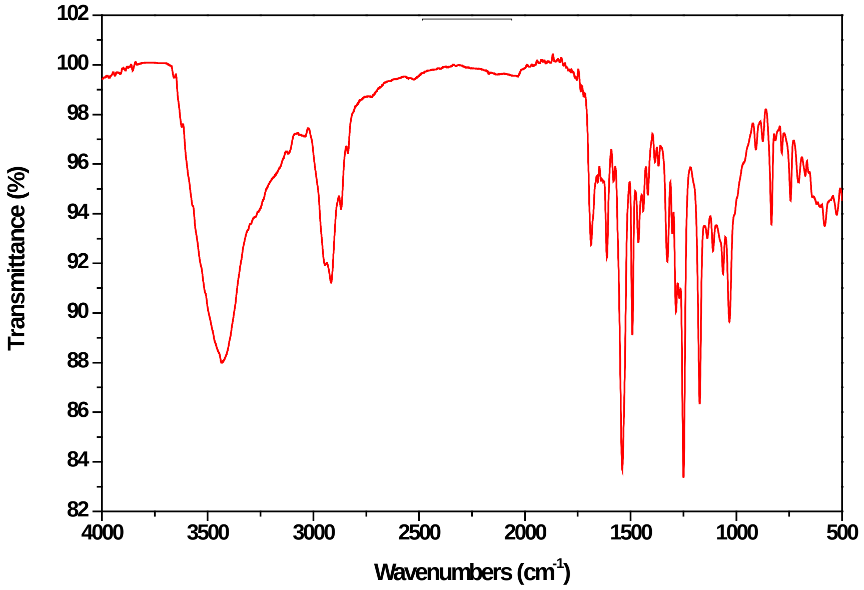

(1S,2S,5S)-6,6-dimethyl-N-(phenylcarbamothioyl)bicyclo[3.1.1]heptane-2-carboxamide (4m)

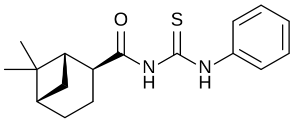

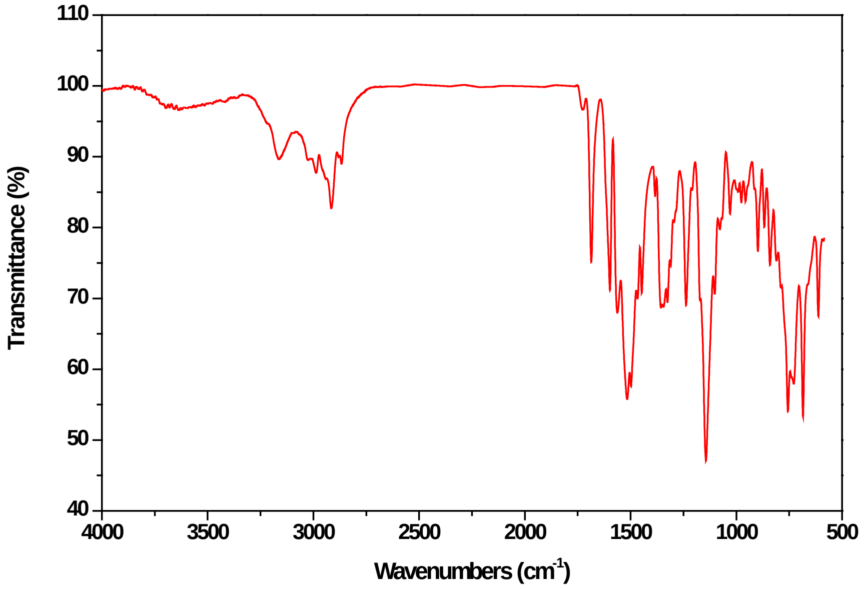

(1S,2S,5S)-N-((2-bromophenyl)carbamothioyl)-6,6-dimethylbicyclo[3.1.1]heptane-2-carboxamide

(4n)

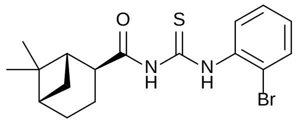

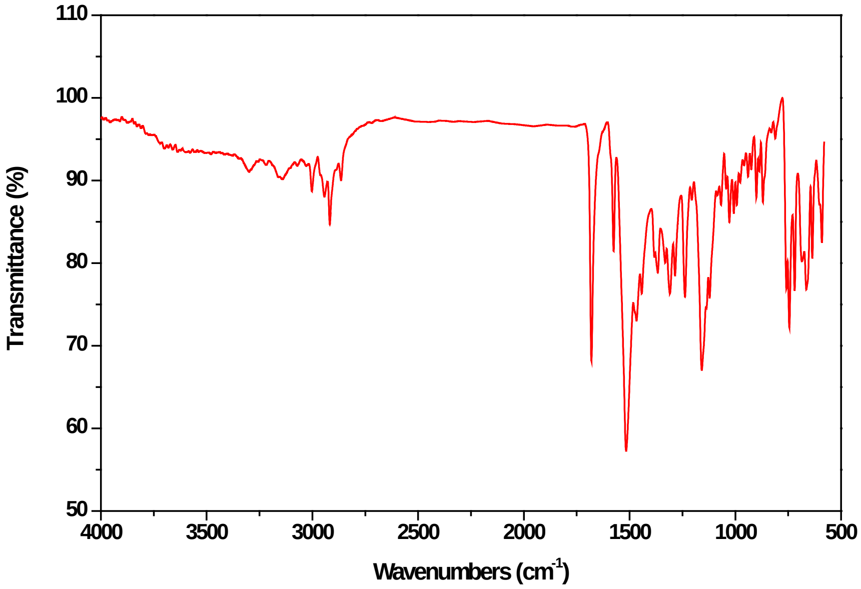

(1S,2S,5S)-N-((4-ethylphenyl)carbamothioyl)-6,6-dimethylbicyclo[3.1.1]heptane-2-carboxamide (4o)

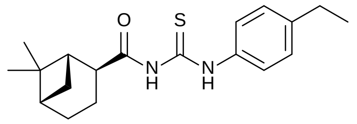

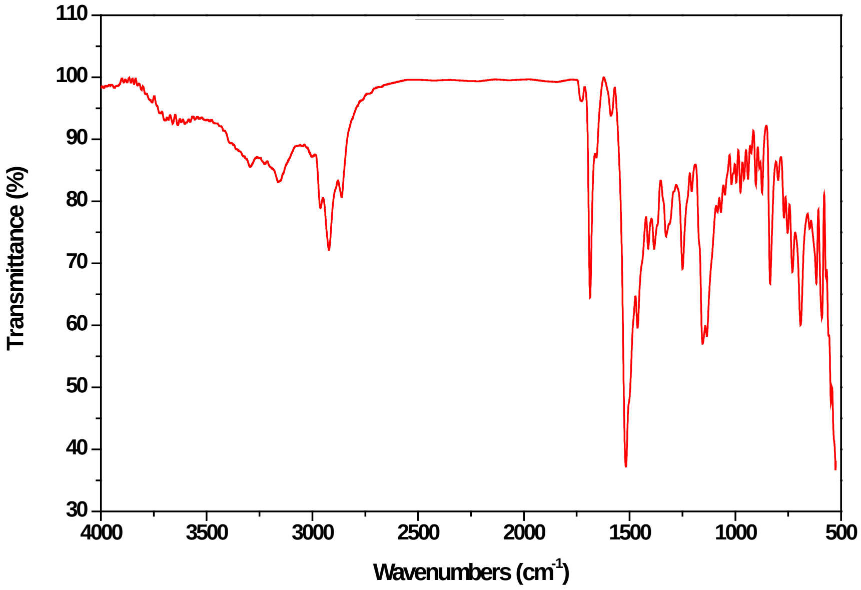

(1S,2S,5S)-6,6-dimethyl-N-((4-(trifluoromethyl)phenyl)carbamothioyl)bicyclo[3.1.1]heptane-2-carboxamide (4p)

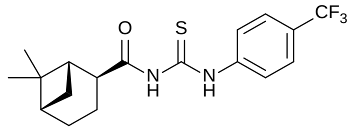

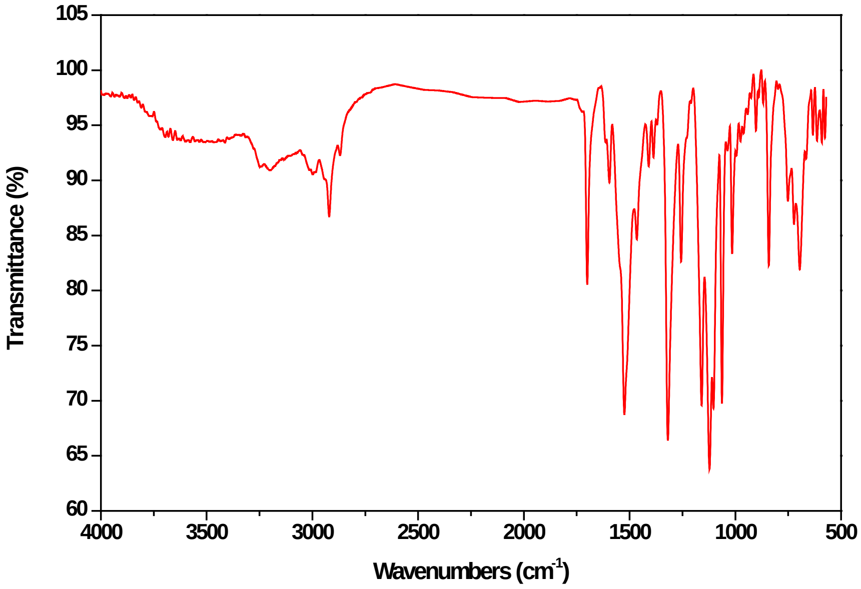

(1S,2S,5S)-N-((2,6-difluorophenyl)carbamothioyl)-6,6-dimethylbicyclo[3.1.1]heptane-2-carboxamide (4q)

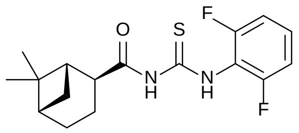

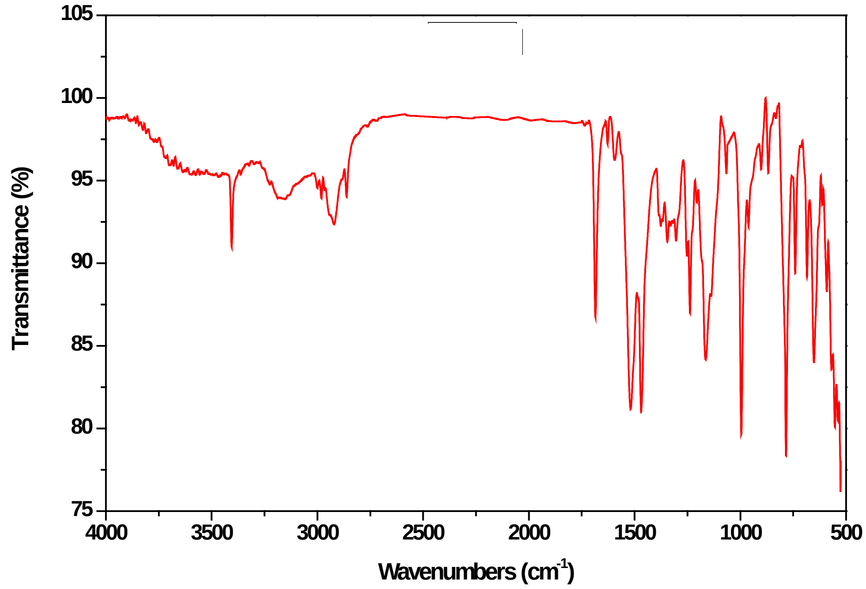

(1S,2S,5S)-N-((4-fluorophenyl)carbamothioyl)-6,6-dimethylbicyclo[3.1.1]heptane-2-carboxamide (4r)

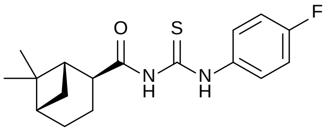

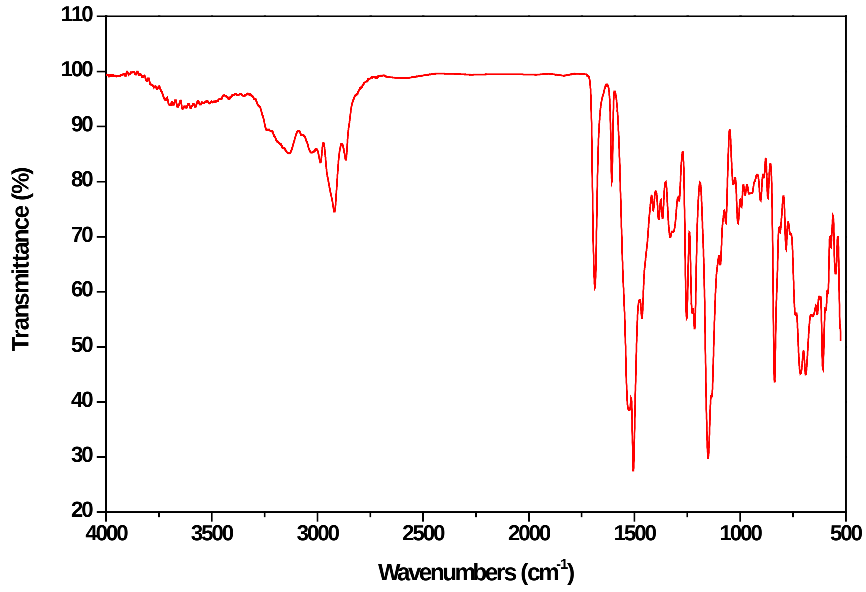

(1S,2S,5S)-N-((4-(4-fluorophenyl)thiazol-2-yl)carbamothioyl)-6,6-dimethylbicyclo[3.1.1]heptane-2-carboxamide (4s)

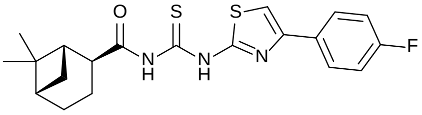

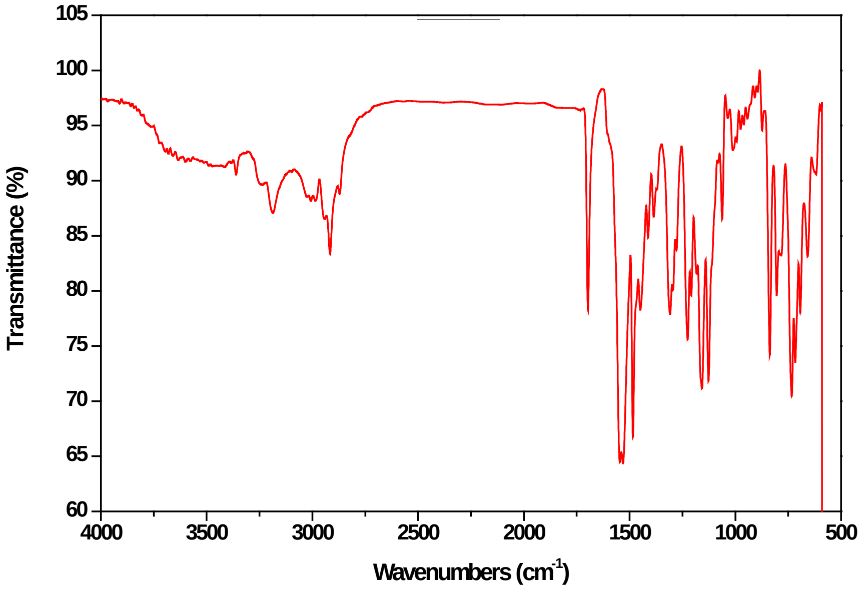

(1S,2S,5S)-6,6-dimethyl-N-((4-(4-nitrophenyl)thiazol-2-yl)carbamothioyl)bicyclo[3.1.1]heptane-2-carboxamide (4t)

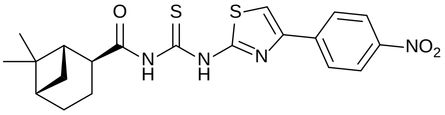

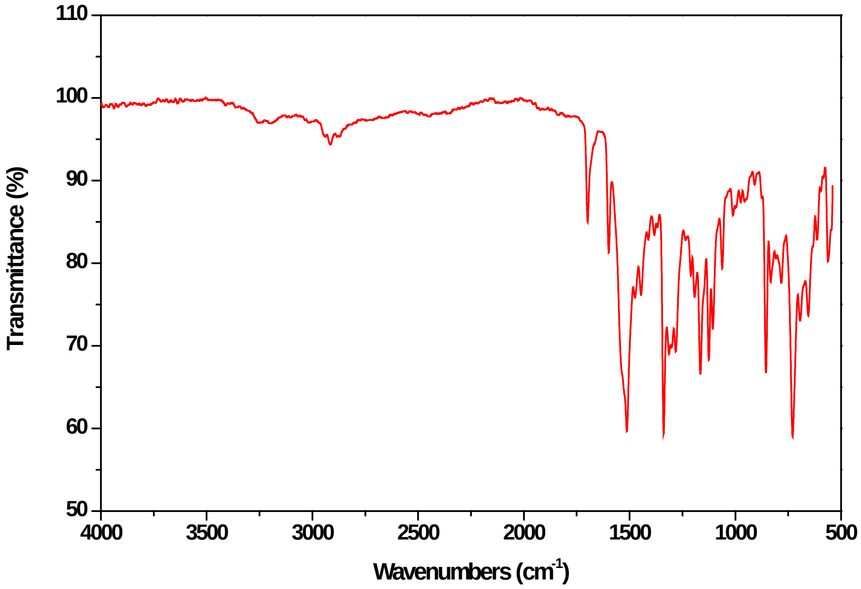

NMR Spectra of 3 and 4a-4t

(1S,2S,5S)-6,6-dimethylbicyclo[3.1.1]heptane-2-carboxylic acid (3)

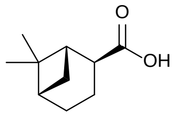

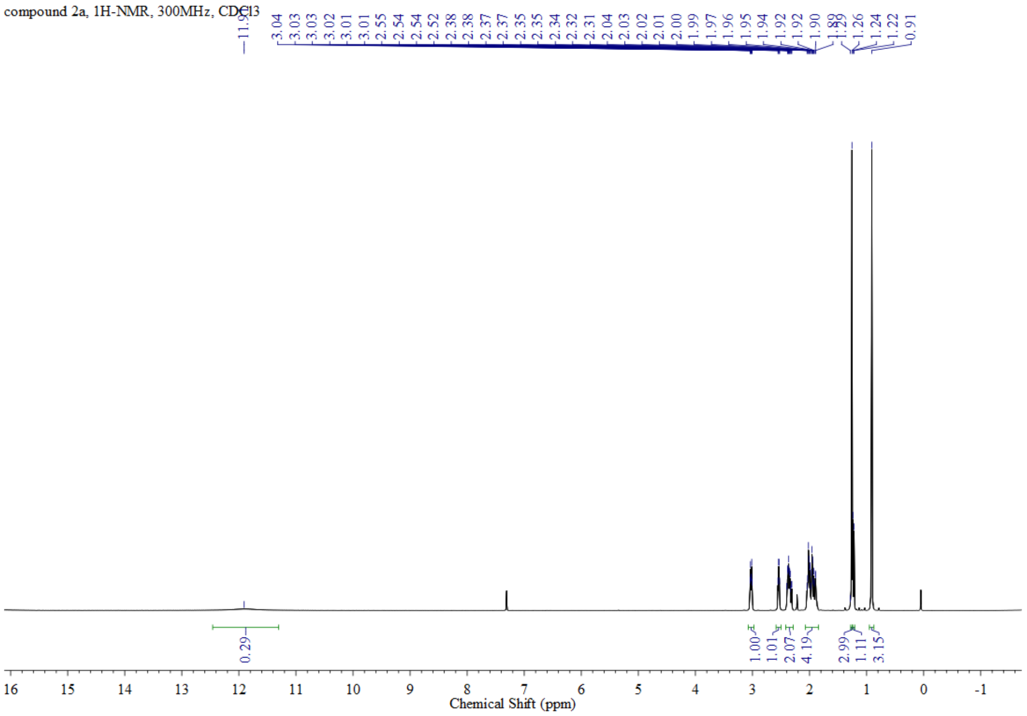

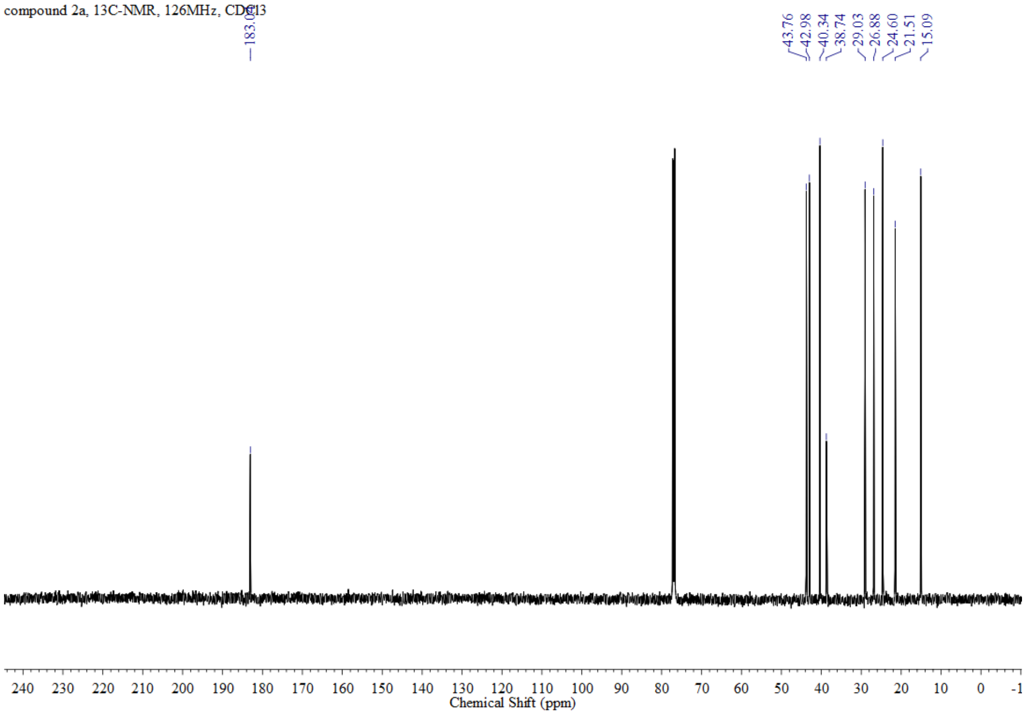

(1S,2S,5S)-6,6-dimethyl-N-phenylbicyclo[3.1.1]heptane-2-carboxamide (4a)

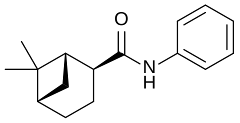

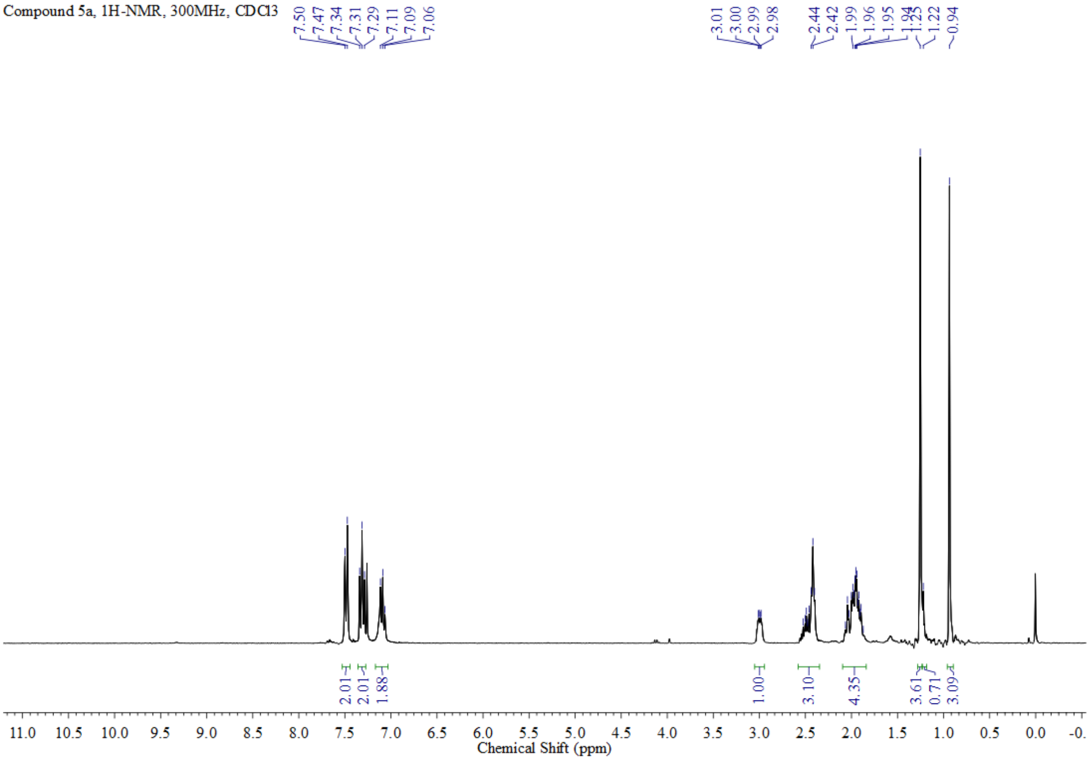

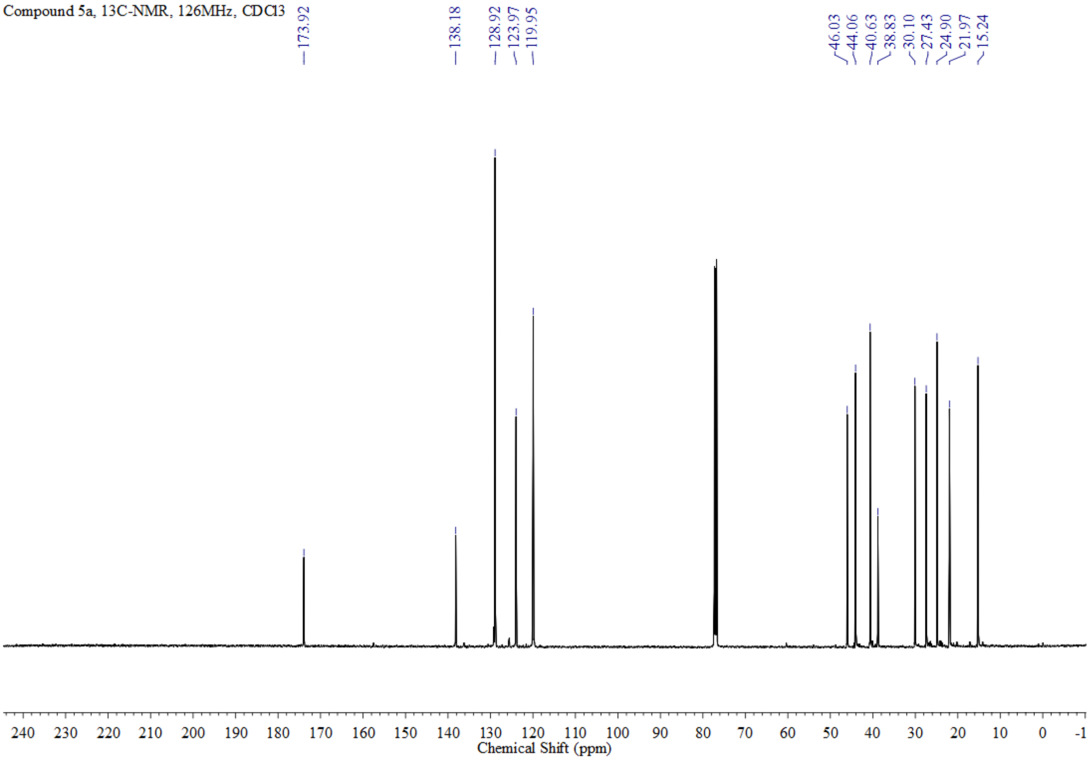

(1S,2S,5S)-N-(2-bromophenyl)-6,6-dimethylbicyclo[3.1.1]heptane-2-carboxamide (4b)

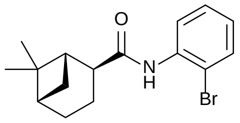

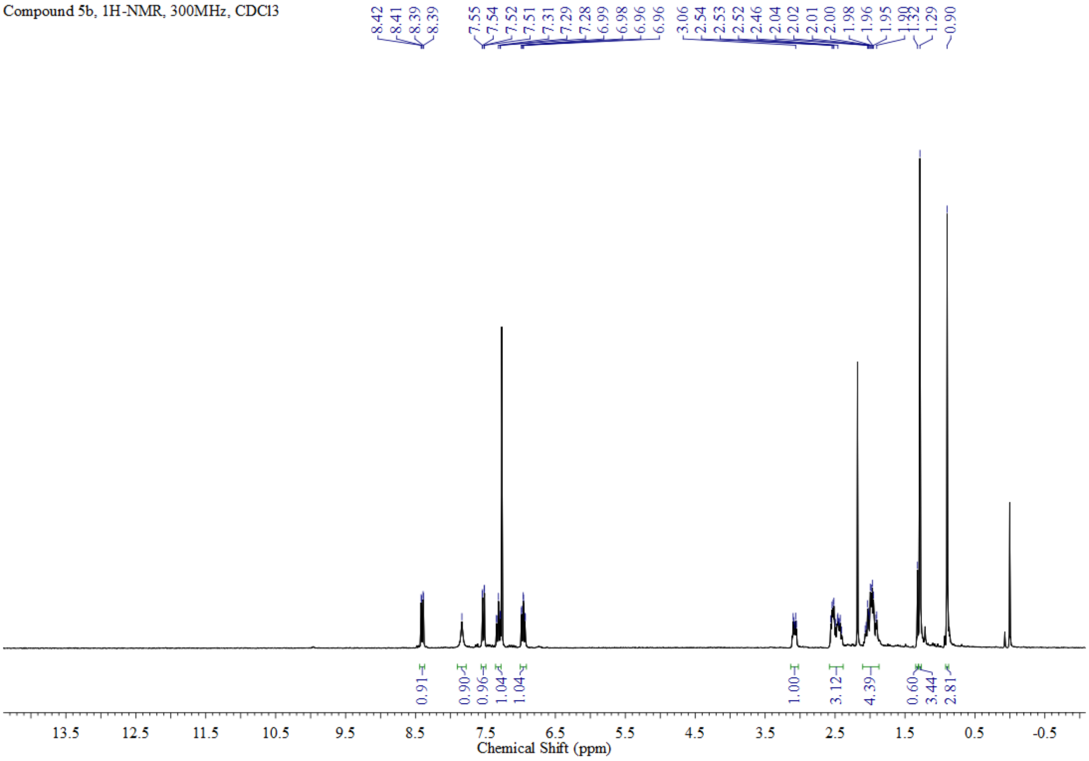

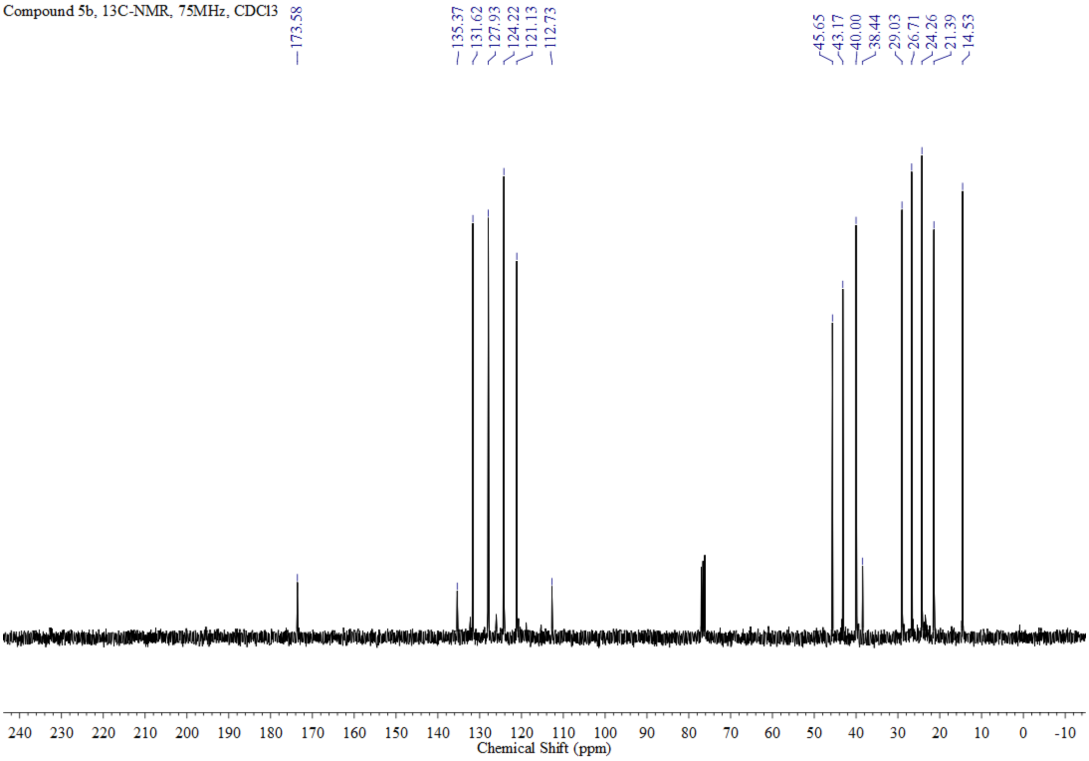

(1S,2S,5S)-N-(3-bromophenyl)-6,6-dimethylbicyclo[3.1.1]heptane-2-carboxamide (4c)

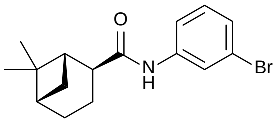

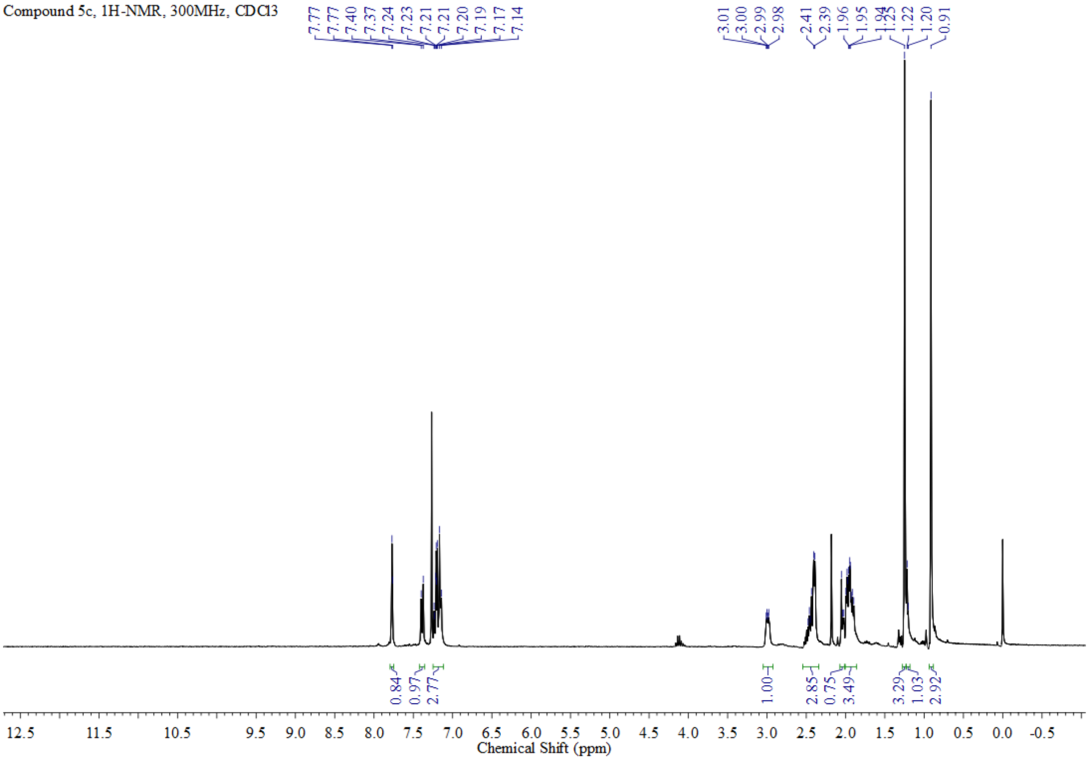

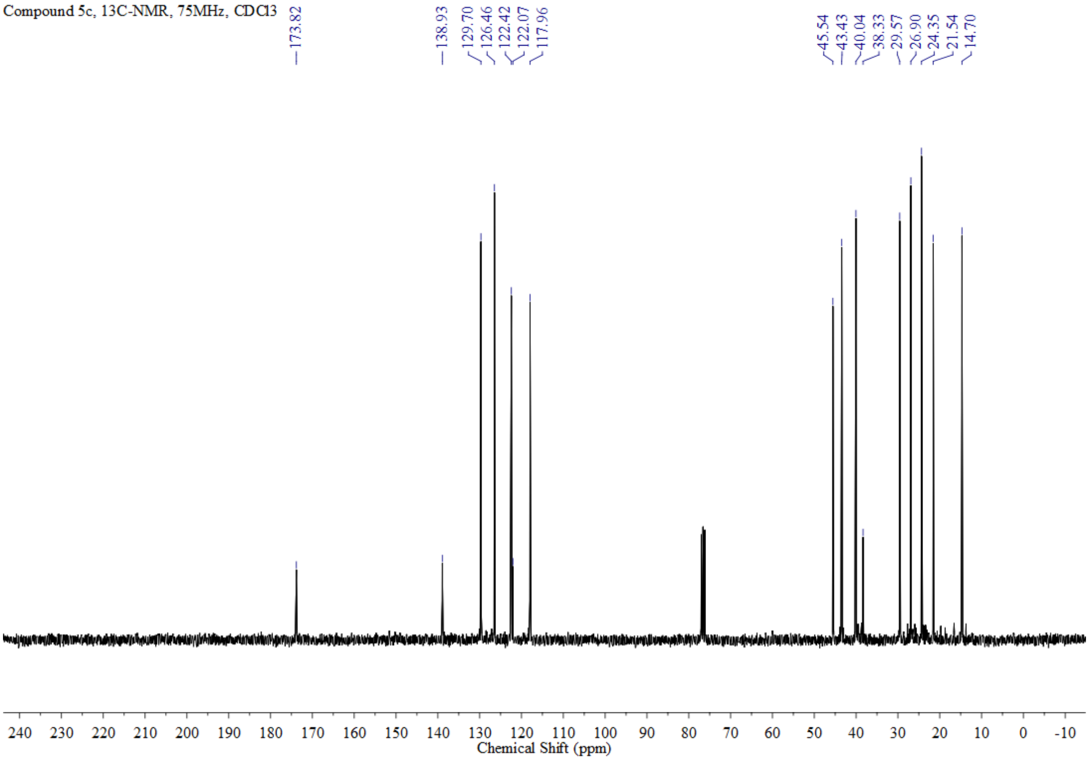

(1S,2S,5S)-N-(4-ethylphenyl)-6,6-dimethylbicyclo[3.1.1]heptane-2-carboxamide (4d)

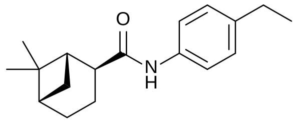

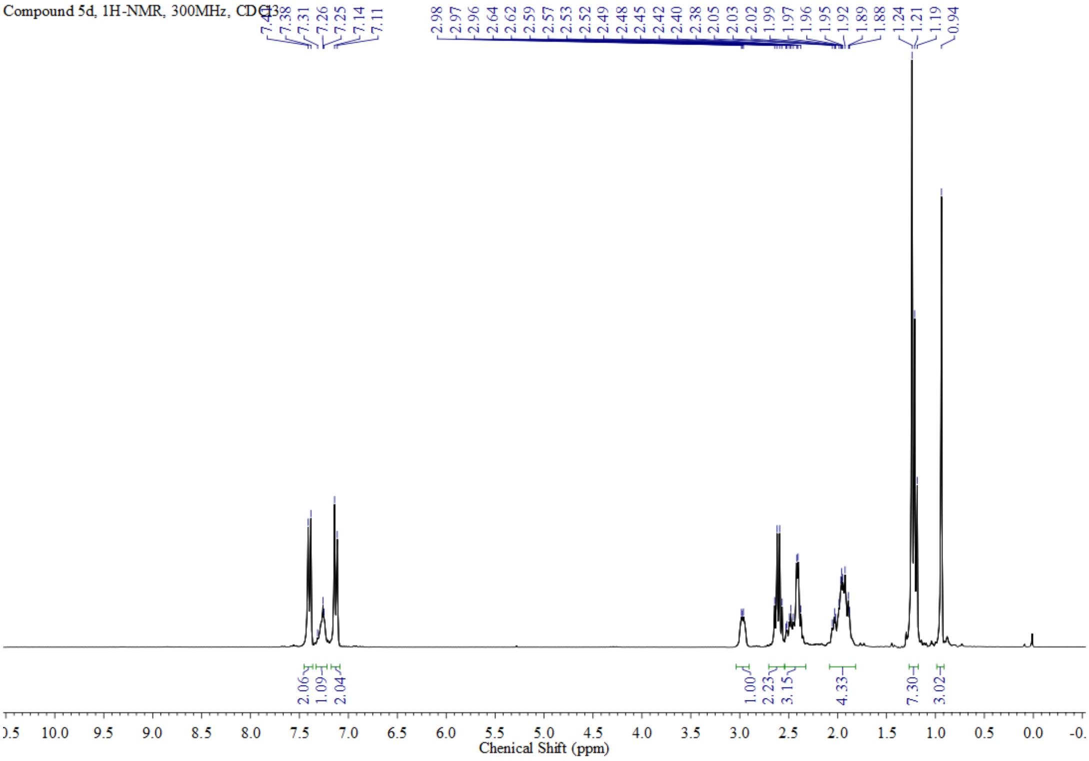

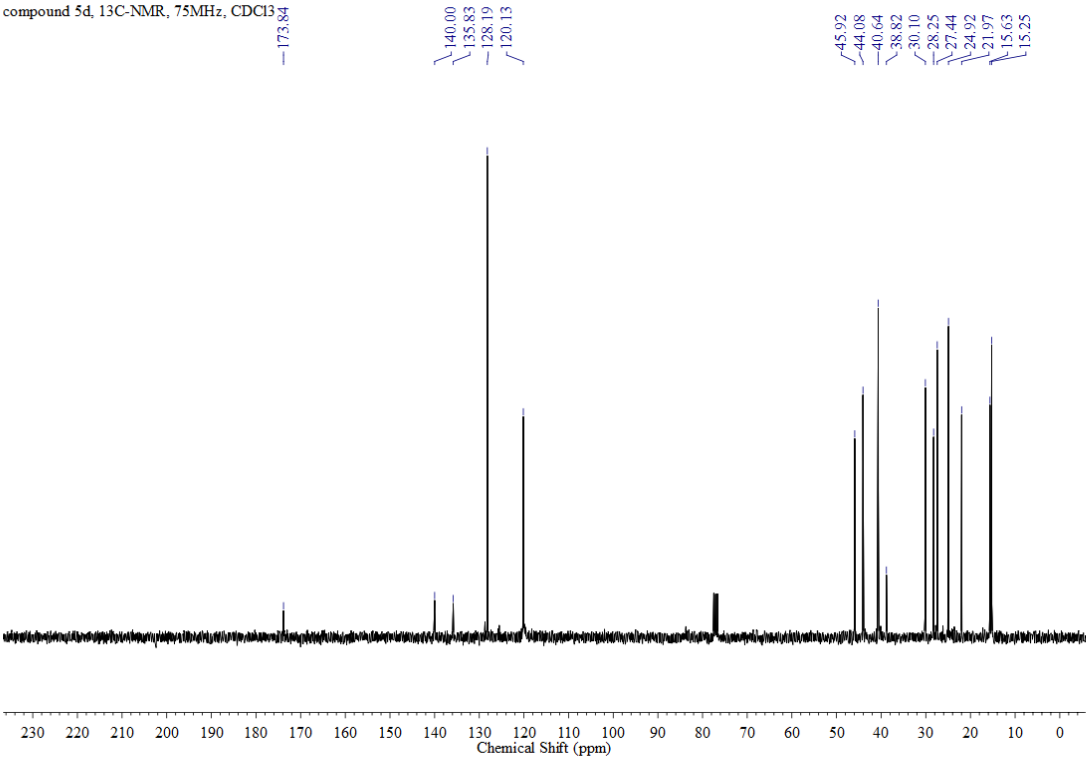

(1S,2S,5S)-6,6-dimethyl-N-(4-(trifluoromethyl)phenyl)bicyclo[3.1.1]heptane-2-carboxamide (4e)

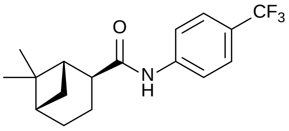

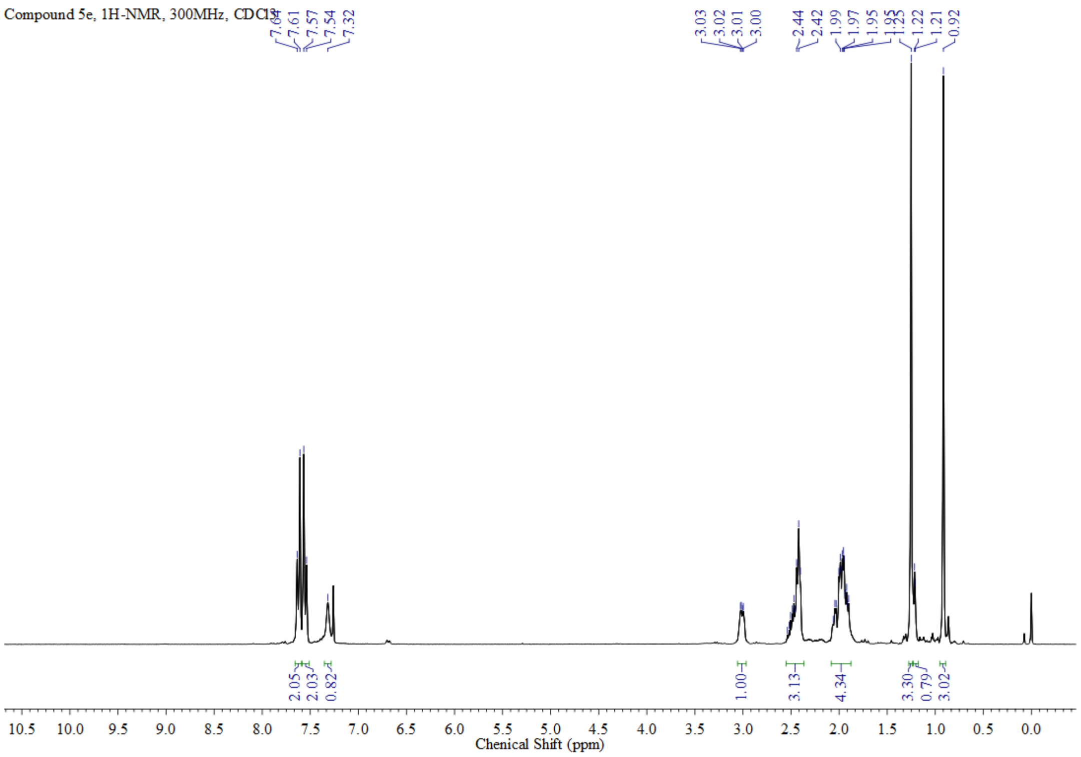

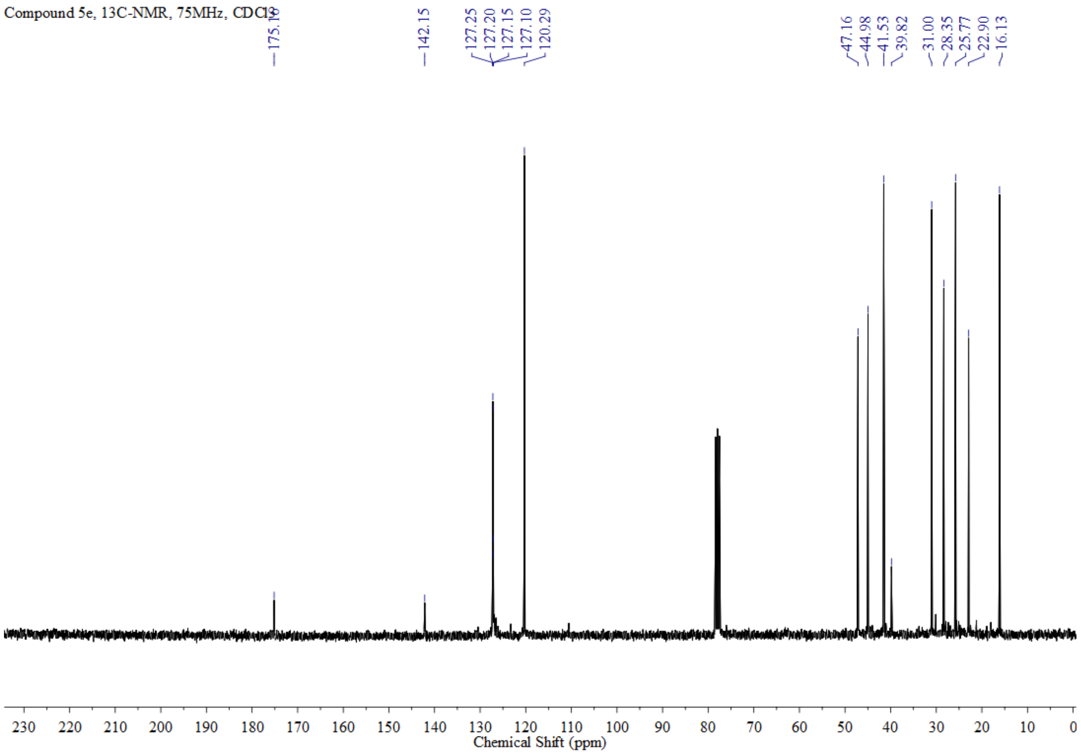

(1S,2S,5S)-N-(2,6-difluorophenyl)-6,6-dimethylbicyclo[3.1.1]heptane-2-carboxamide (4f)

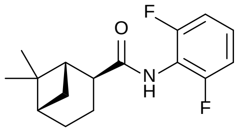

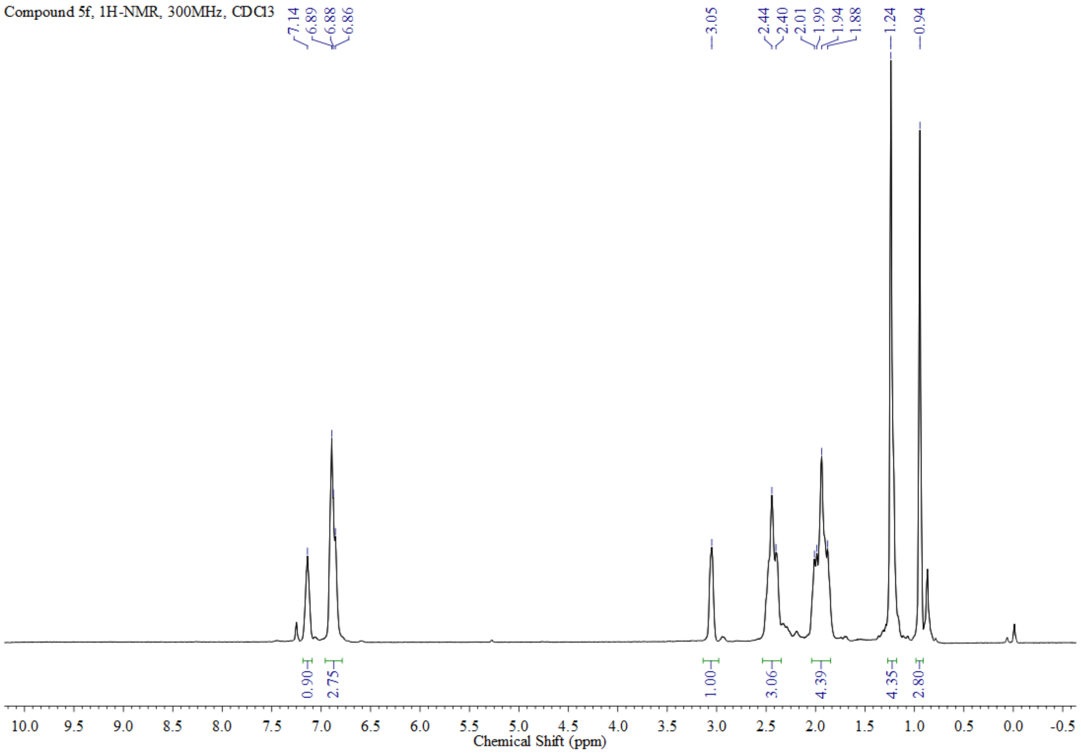

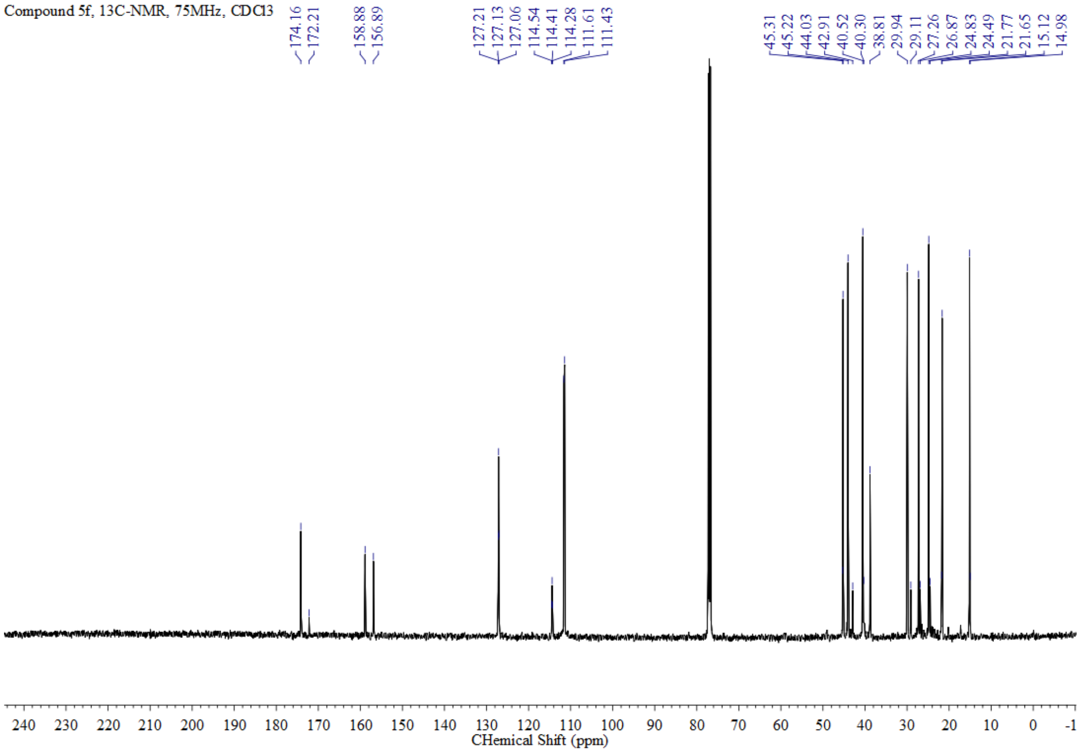

(1S,2S,5S)-N-(4-fluorophenyl)-6,6-dimethylbicyclo[3.1.1]heptane-2-carboxamide (4g)

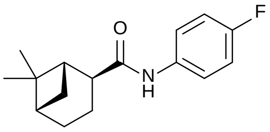

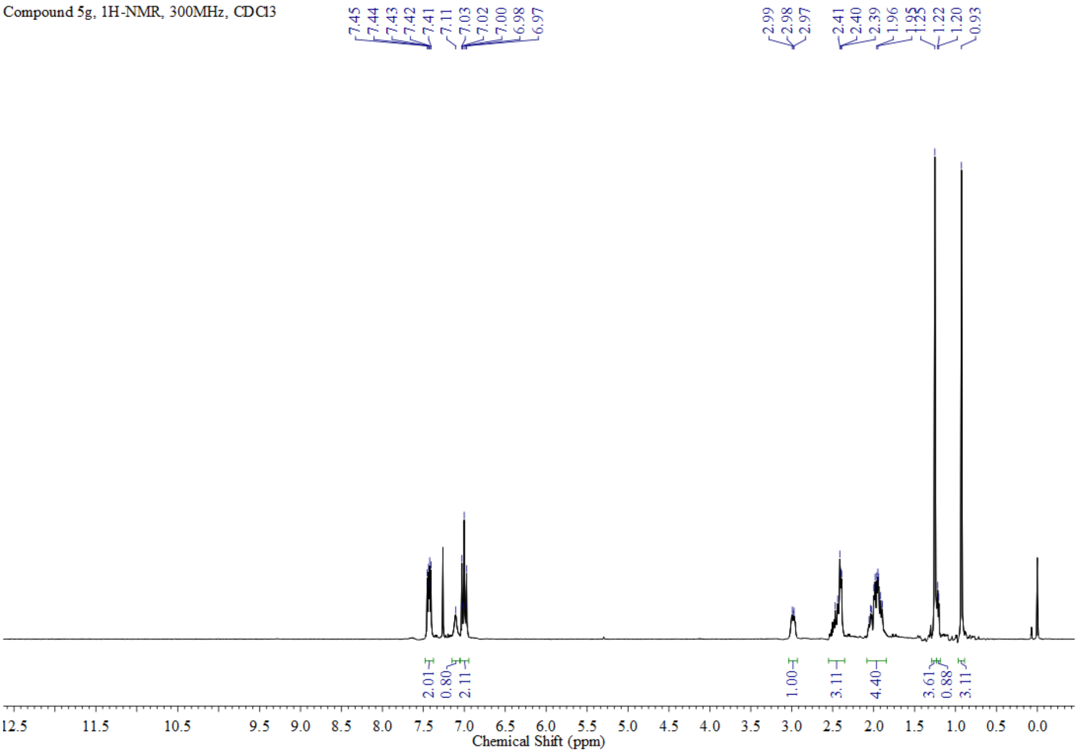

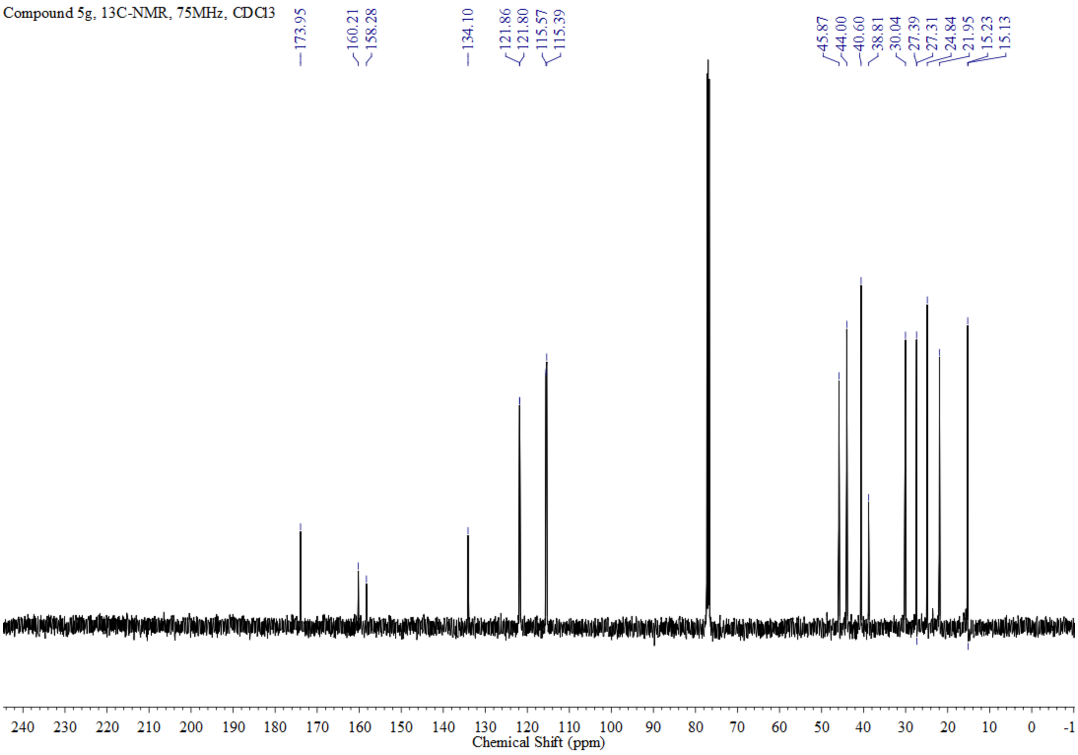

(1S,2S,5S)-6,6-dimethyl-N-(4-nitrophenyl)bicyclo[3.1.1]heptane-2-carboxamide (4h)

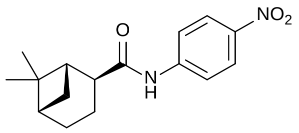

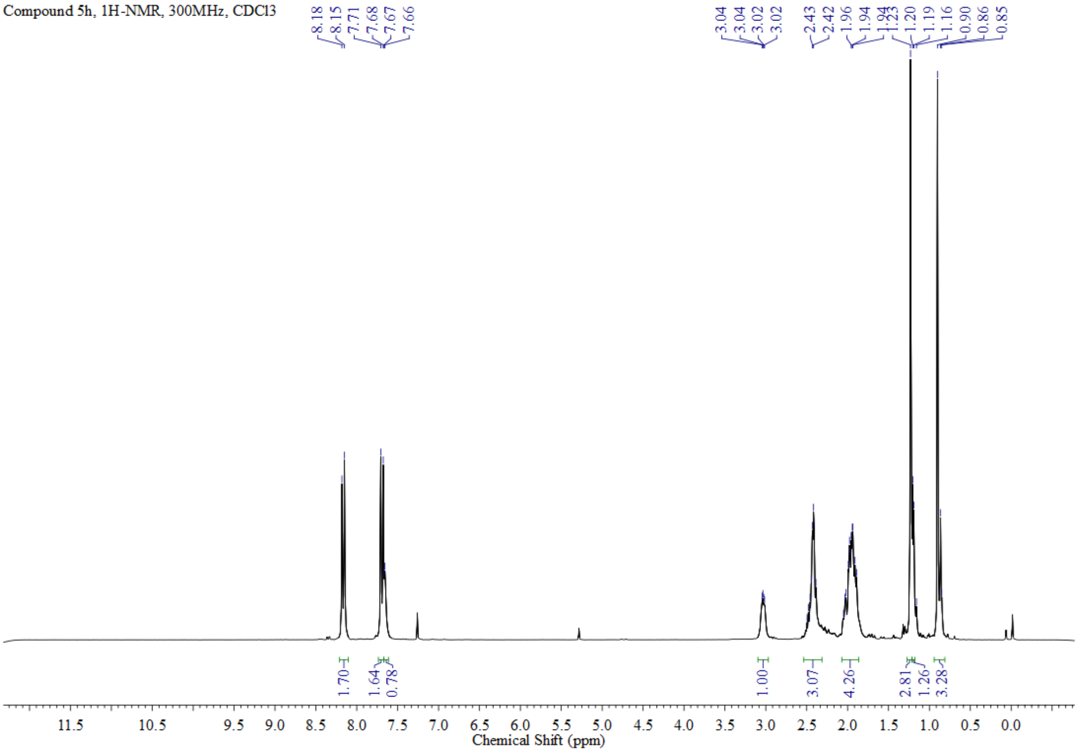

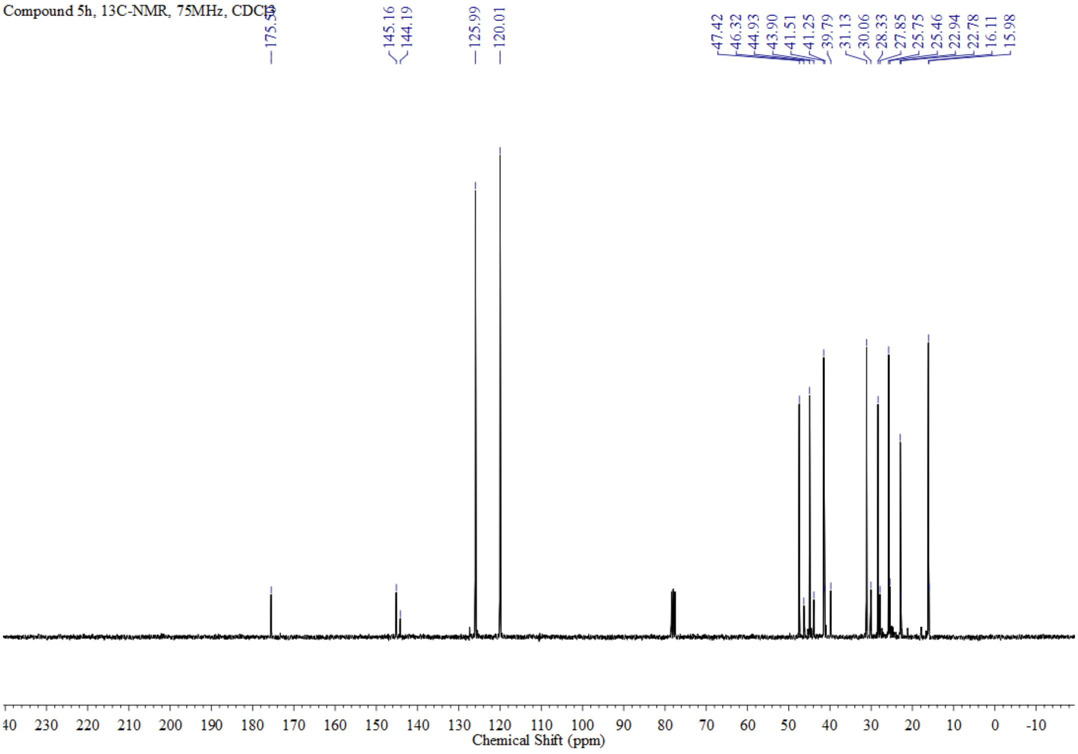

(1S,2S,5S)-6,6-dimethyl-N-(pyridin-2-yl)bicyclo[3.1.1]heptane-2-carboxamide (4i)

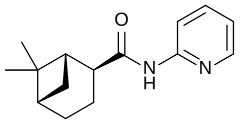

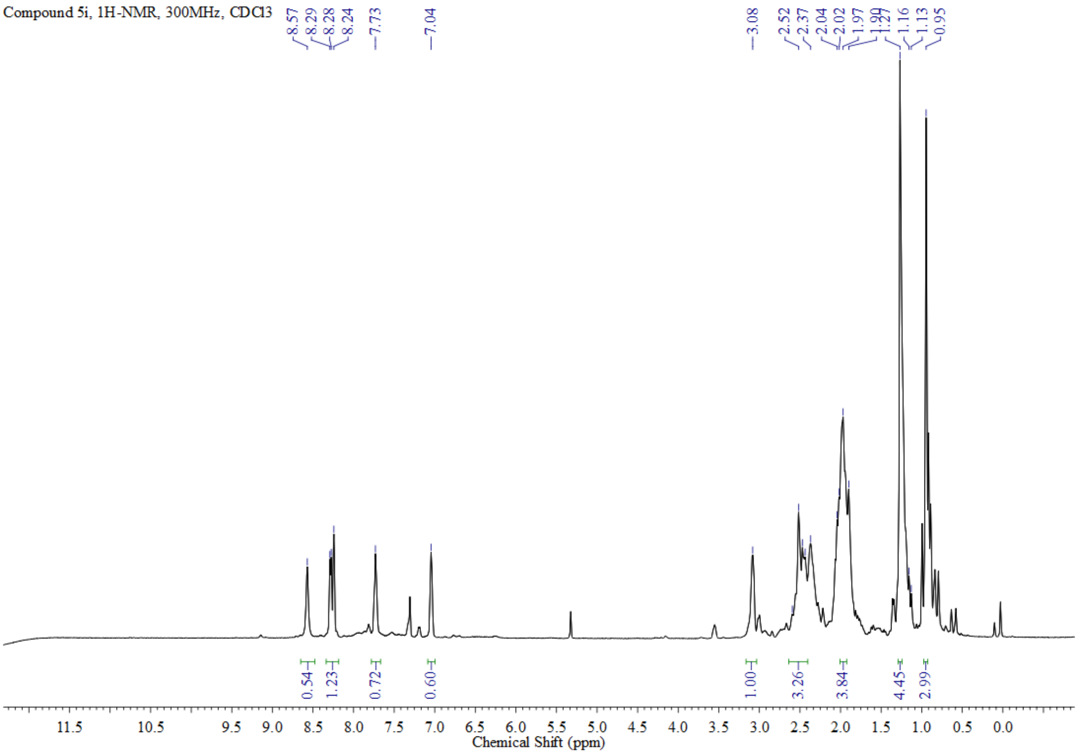

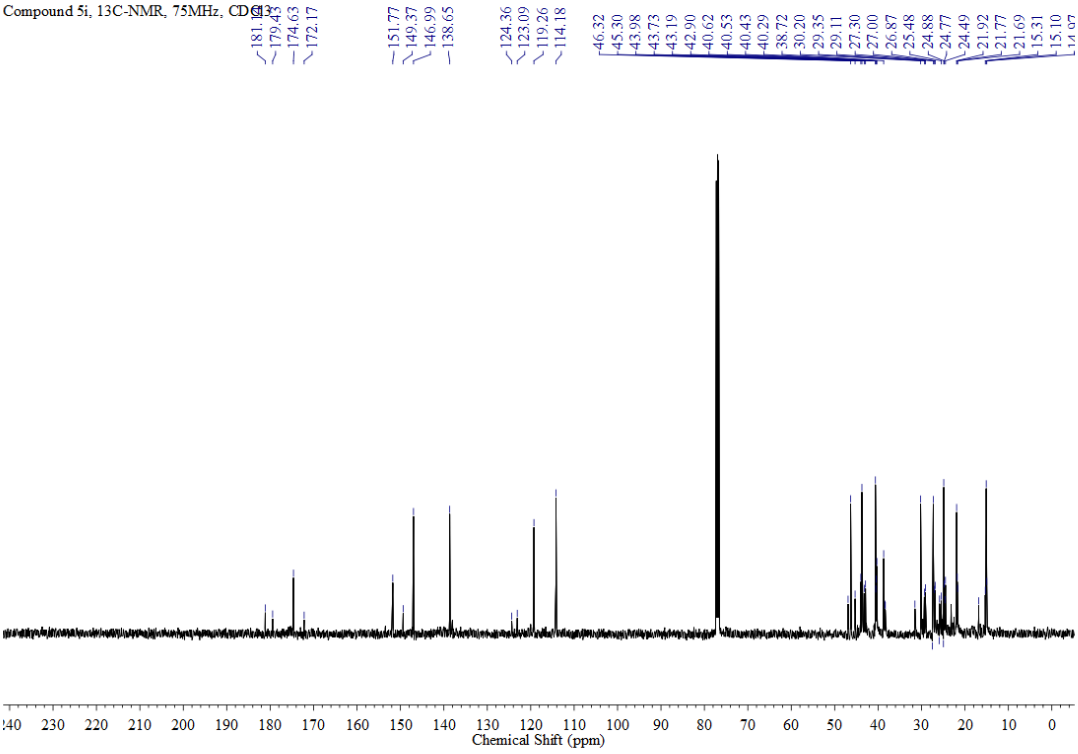

(1S,2S,5S)-N-benzyl-6,6-dimethylbicyclo[3.1.1]heptane-2-carboxamide (4j)

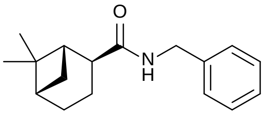

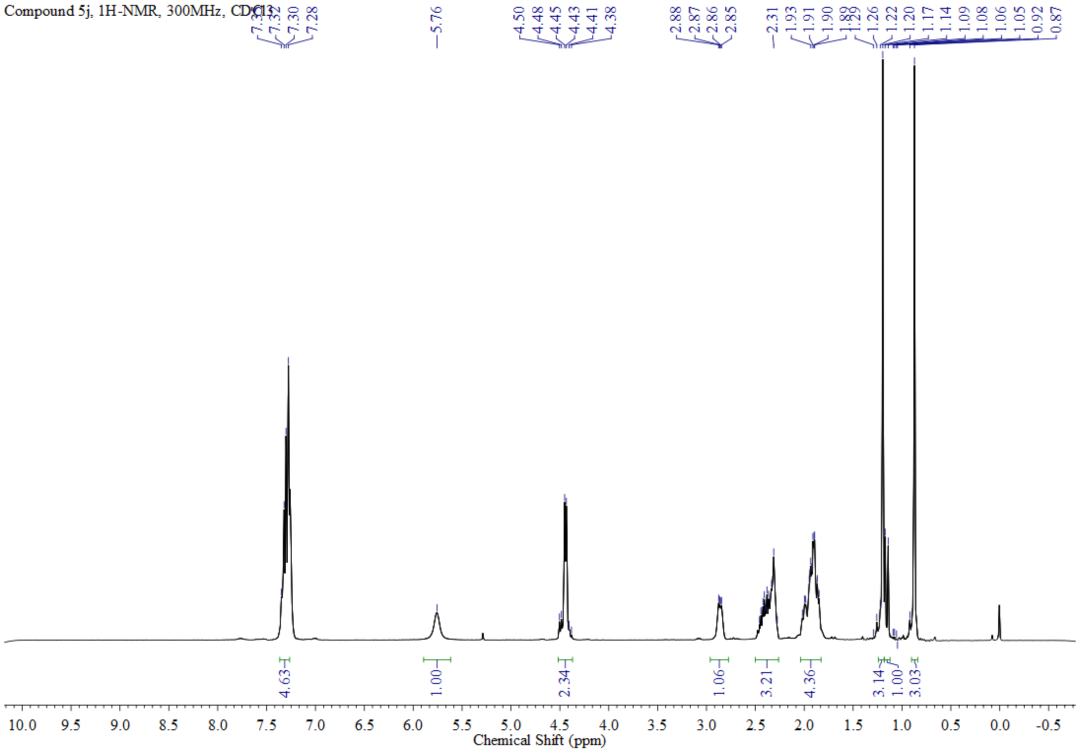

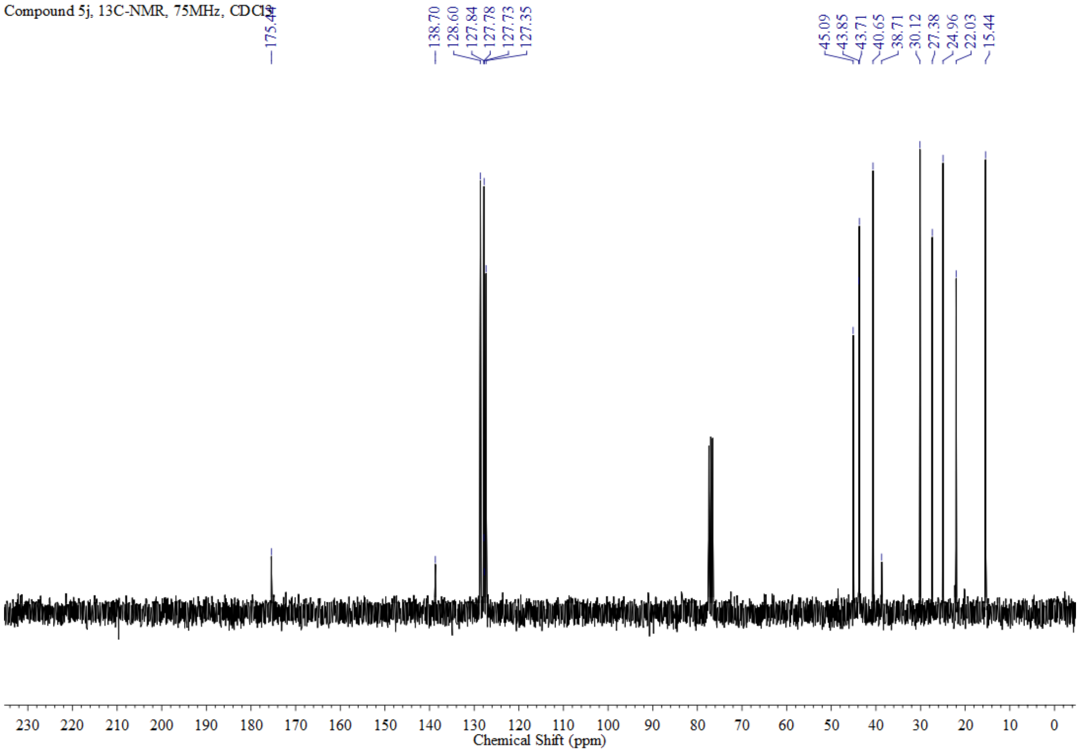

(1S,2S,5S)-N-(4-(4-fluorophenyl)thiazol-2-yl)-6,6-dimethylbicyclo[3.1.1]heptane-2-carboxamide (4k)

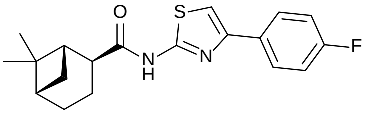

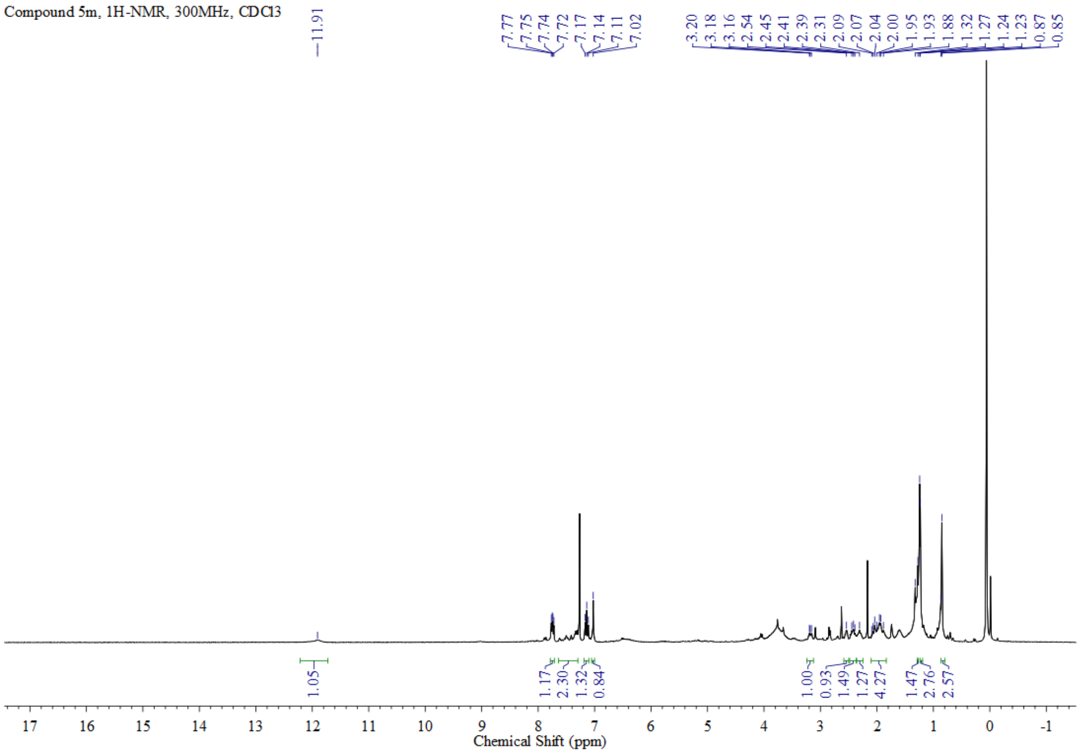

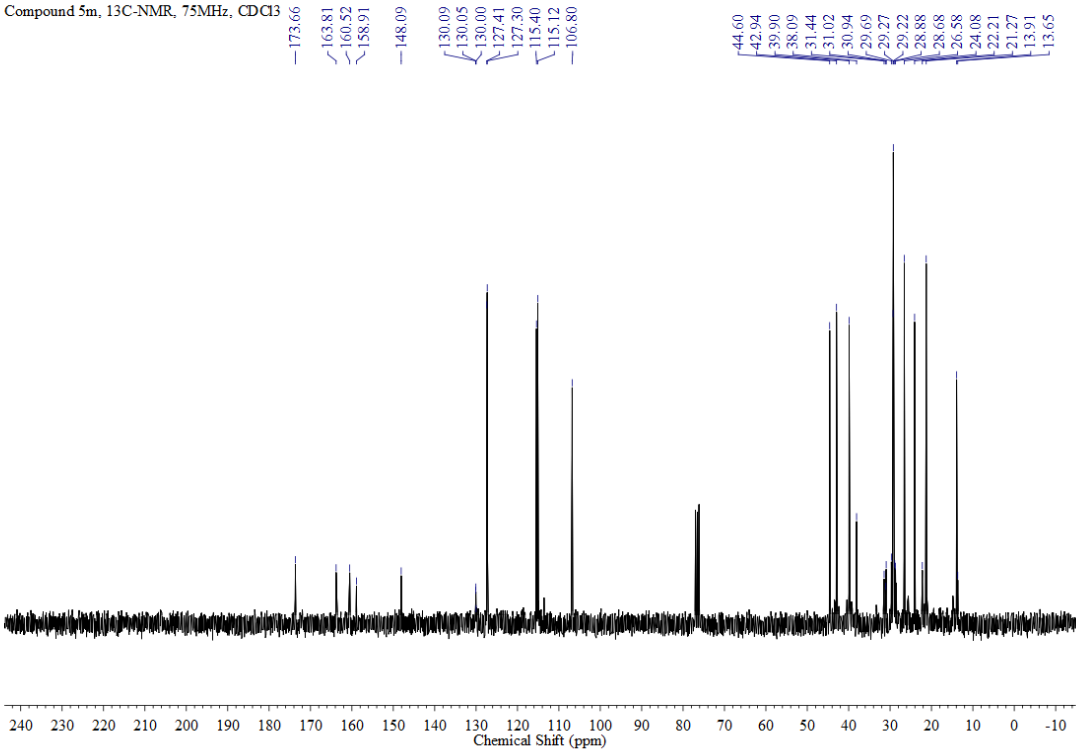

(1S,2S,5S)-N-(4-(4-methoxyphenyl)thiazol-2-yl)-6,6-dimethylbicyclo[3.1.1]heptane-2-carboxamide (4l)

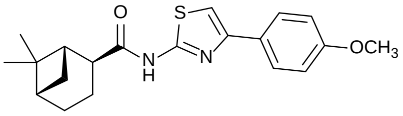

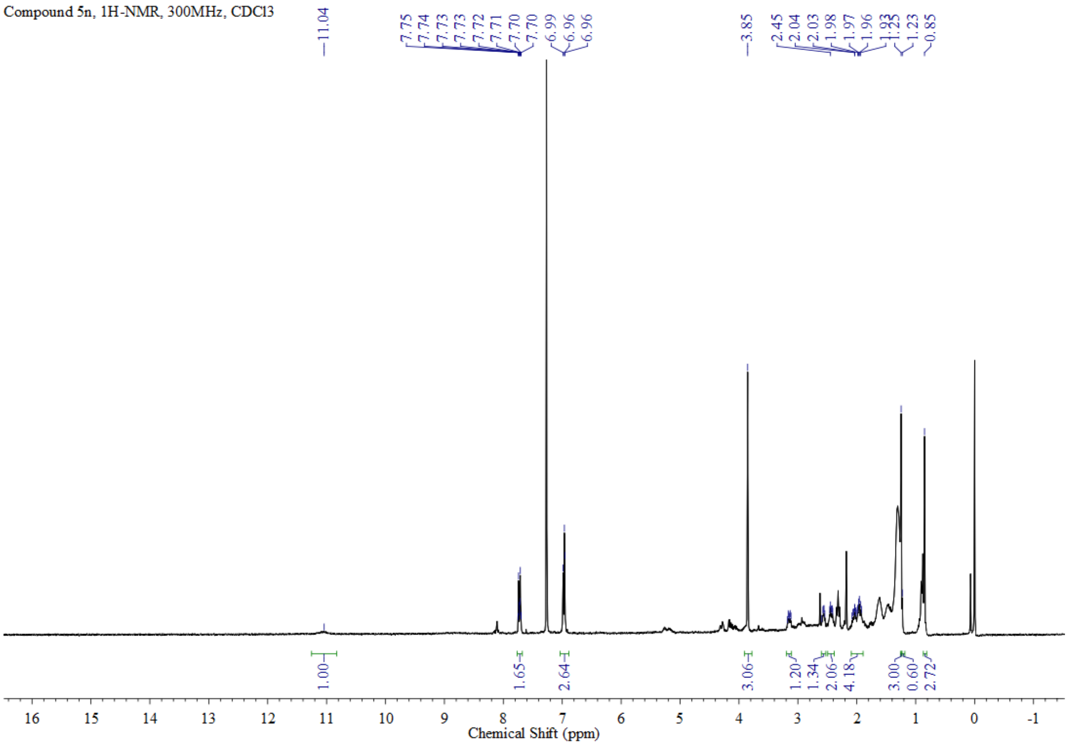

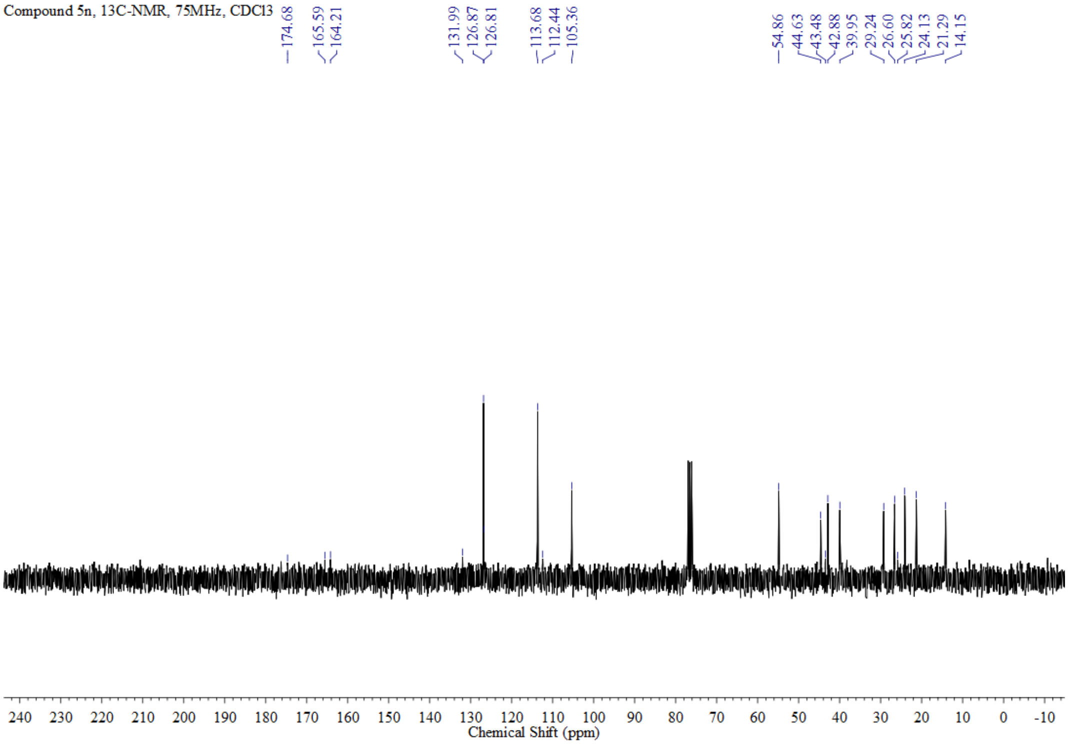

(1S,2S,5S)-6,6-dimethyl-N-(phenylcarbamothioyl)bicyclo[3.1.1]heptane-2-carboxamide (4m)

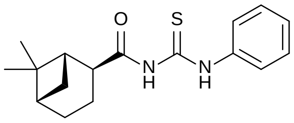

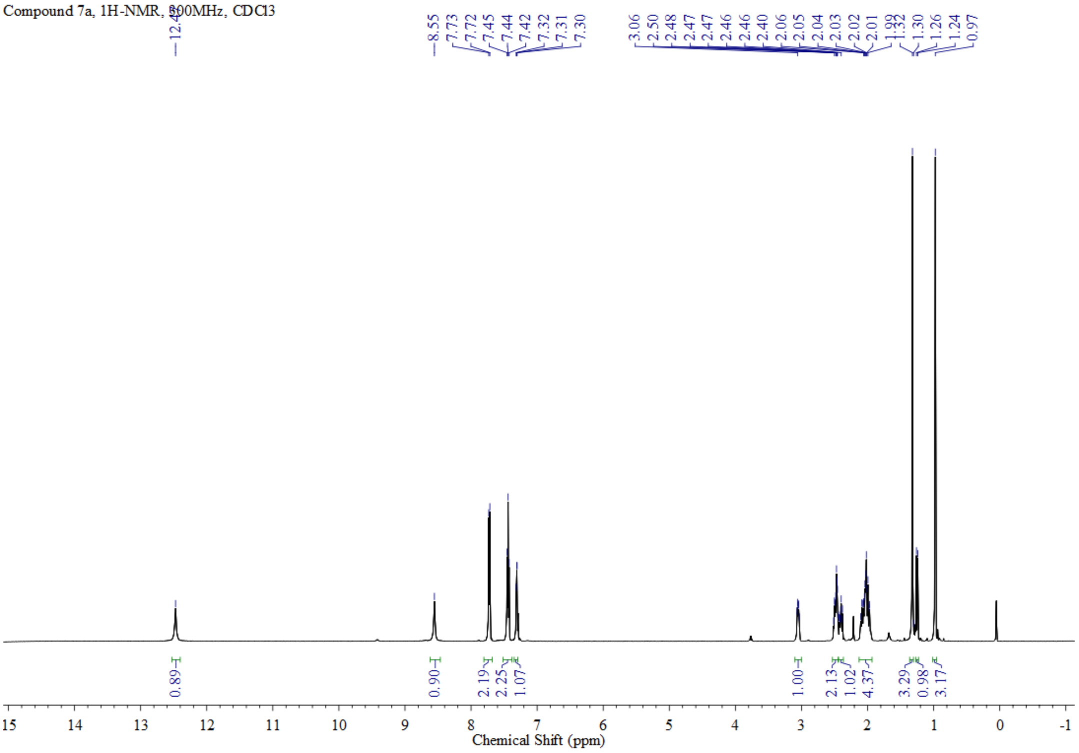

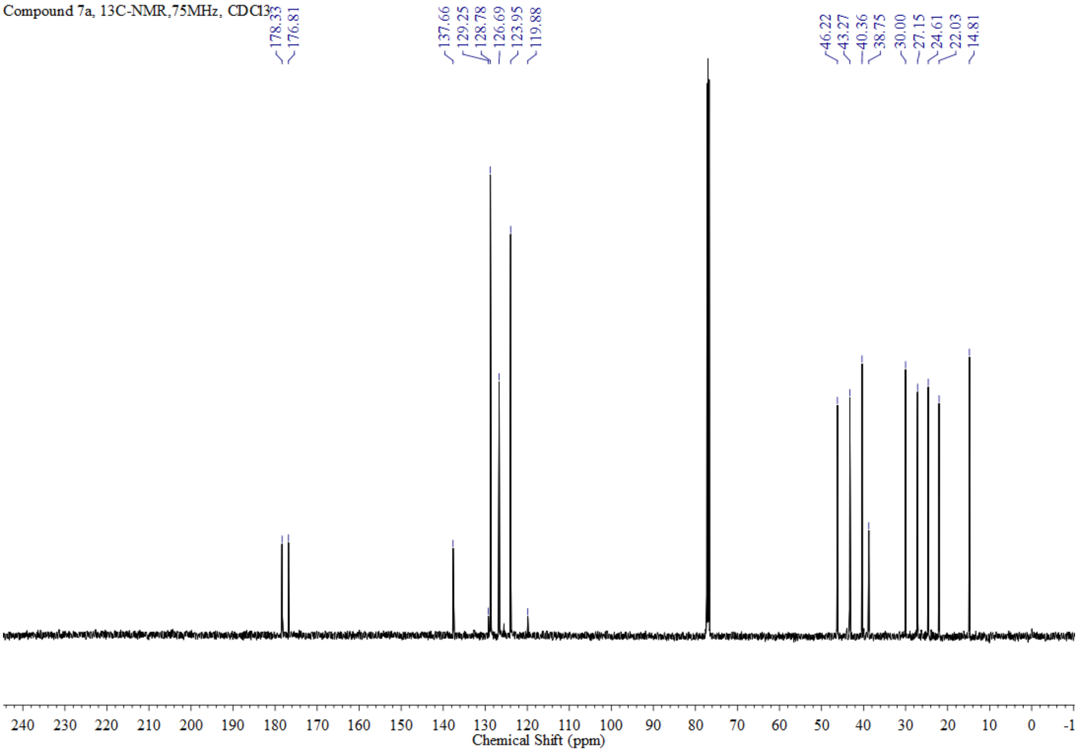

(1S,2S,5S)-N-((2-bromophenyl)carbamothioyl)-6,6-dimethylbicyclo[3.1.1]heptane-2-carboxamide (4n)

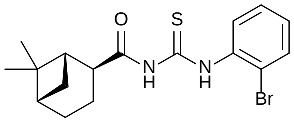

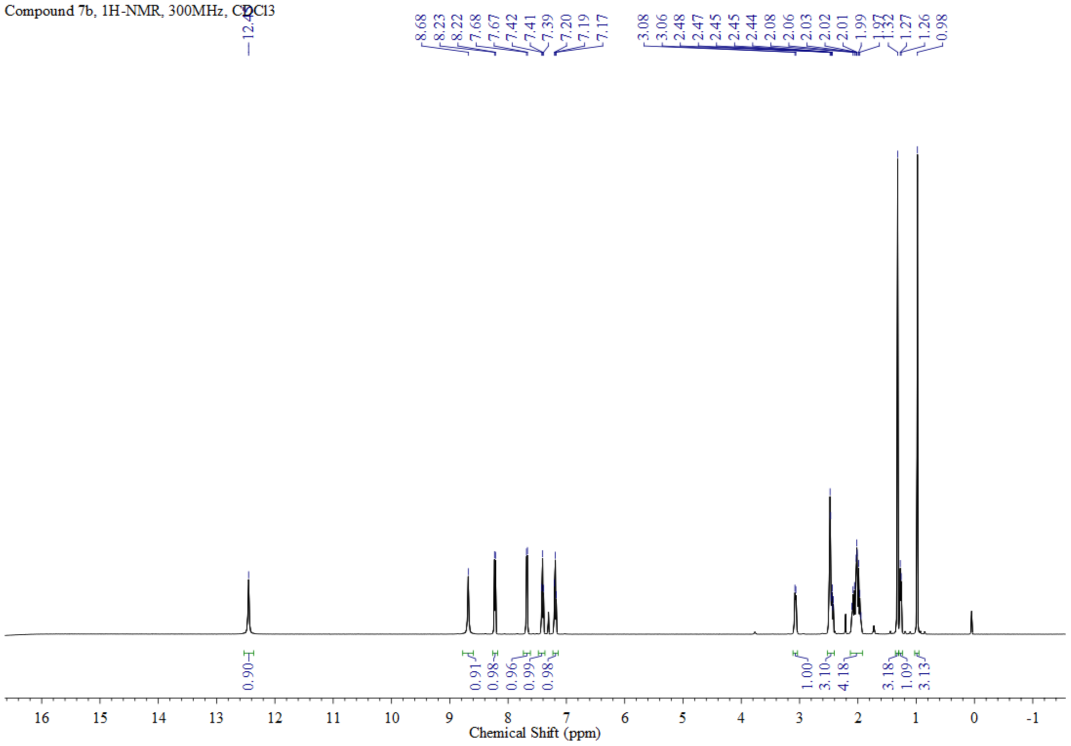

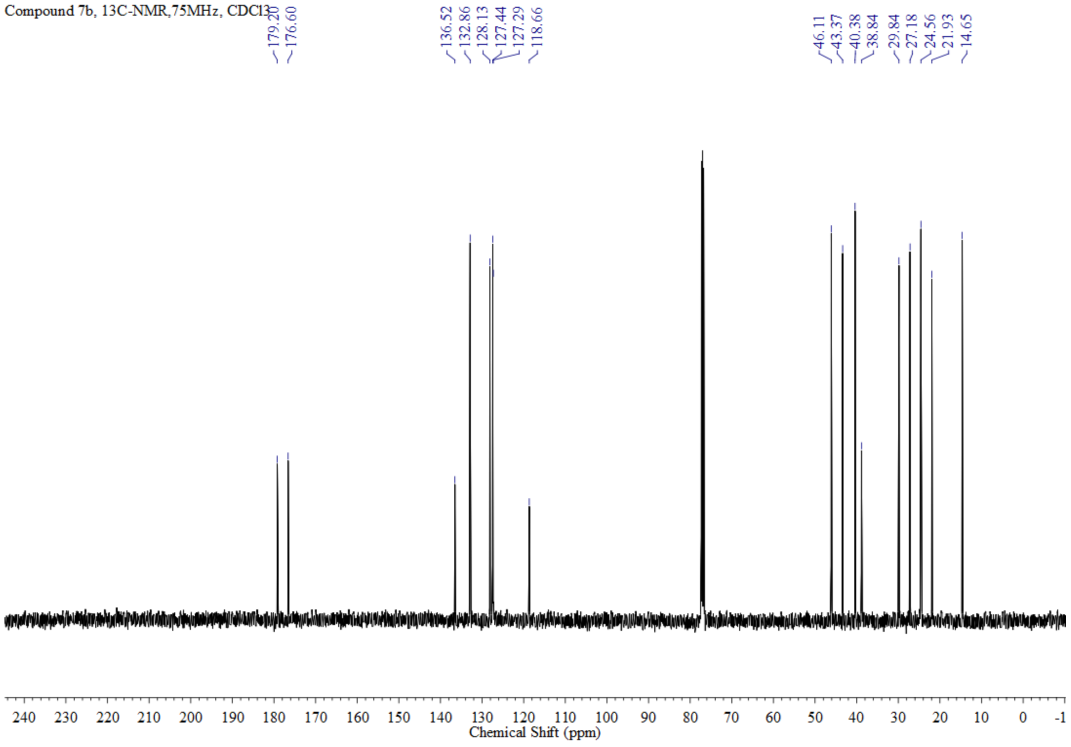

(1S,2S,5S)-N-((4-ethylphenyl)carbamothioyl)-6,6-dimethylbicyclo[3.1.1]heptane-2-carboxamide (4o)

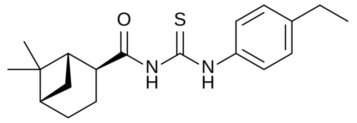

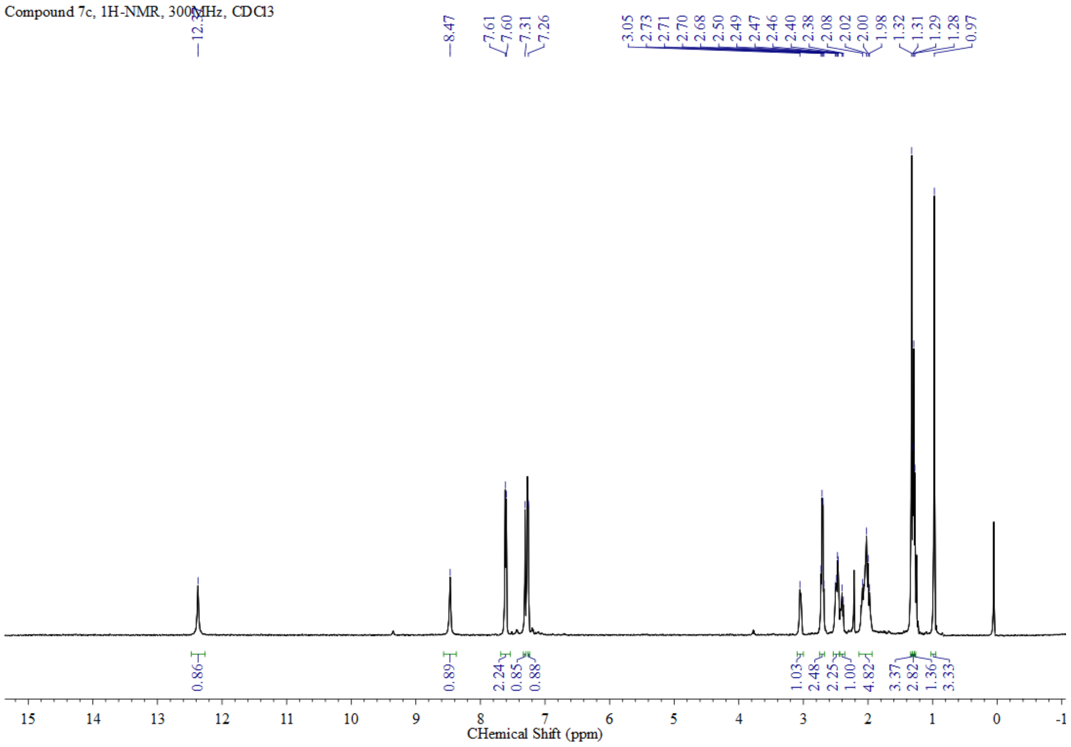

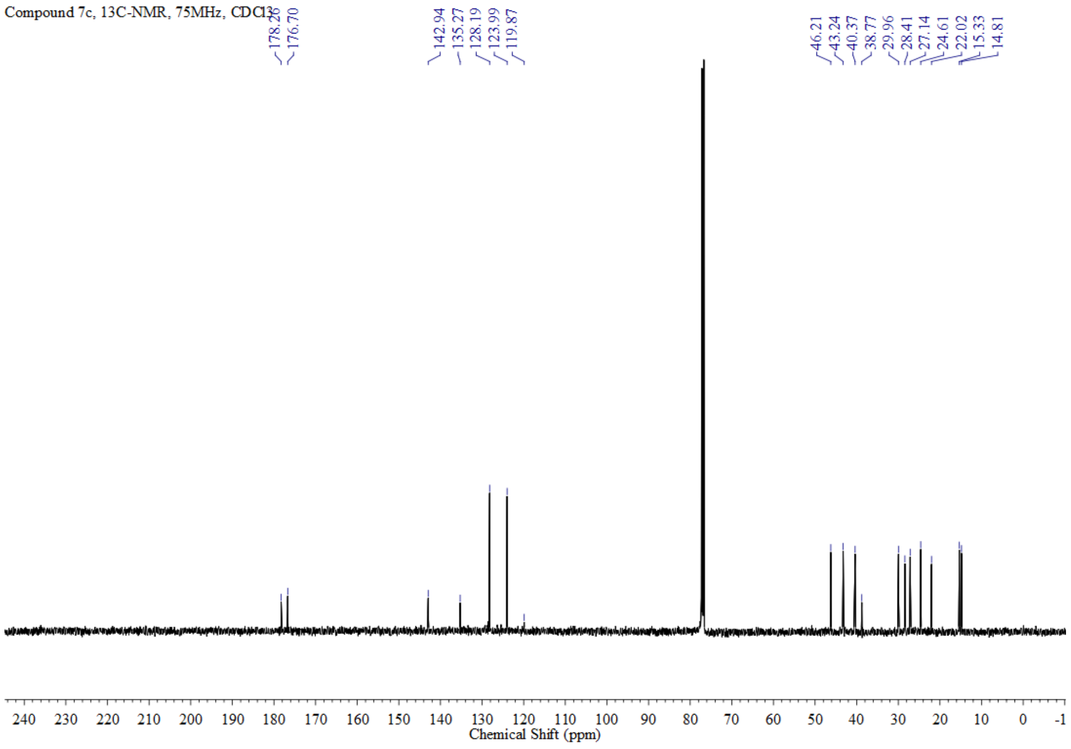

(1S,2S,5S)-6,6-dimethyl-N-((4-(trifluoromethyl)phenyl)carbamothioyl)bicyclo[3.1.1]heptane-2-carboxamide (4p)

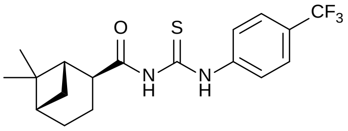

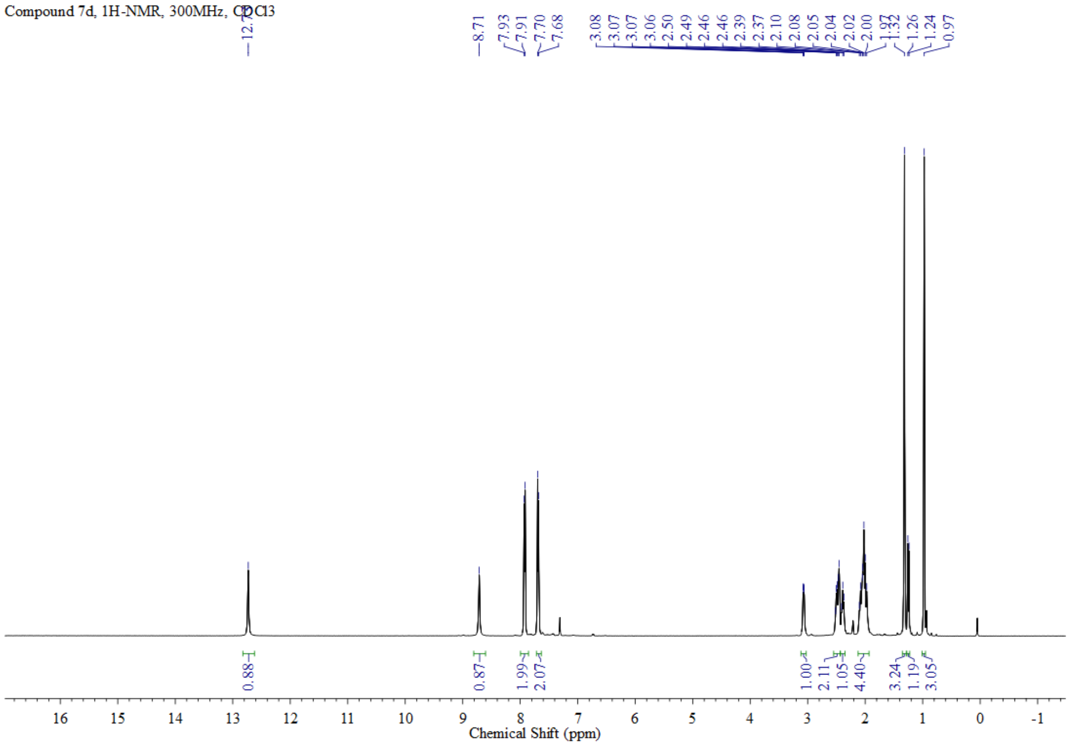

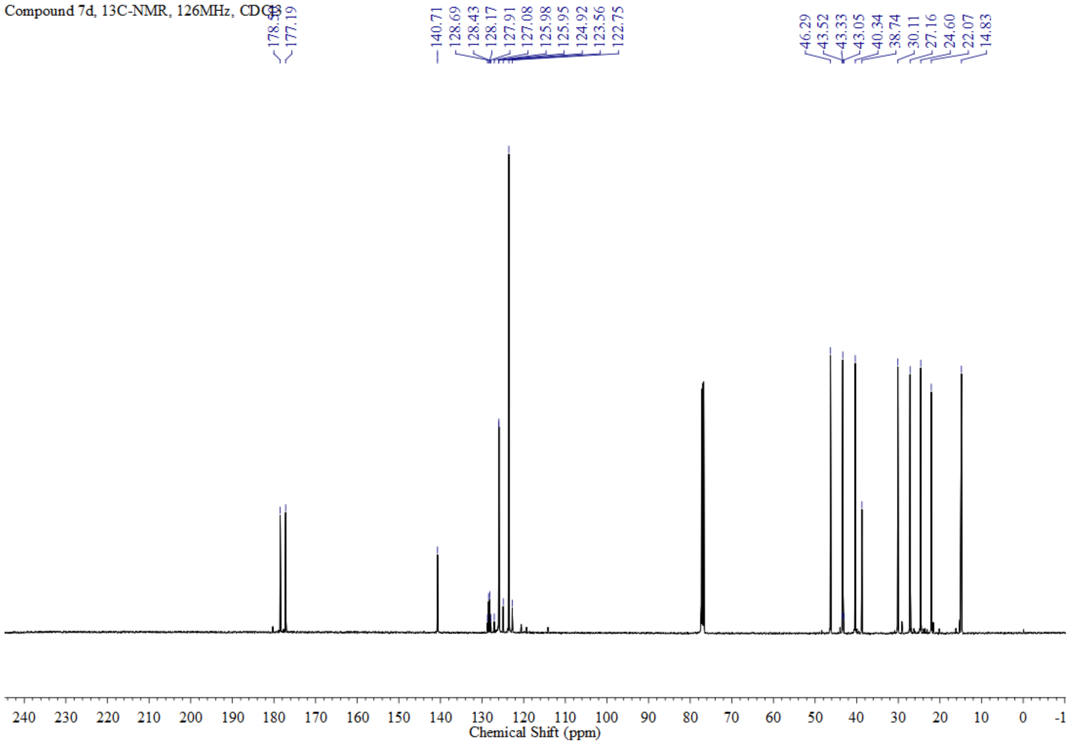

(1S,2S,5S)-N-((2,6-difluorophenyl)carbamothioyl)-6,6-dimethylbicyclo[3.1.1]heptane-2-carboxamide (4q)

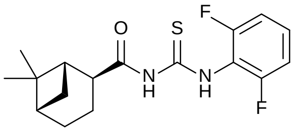

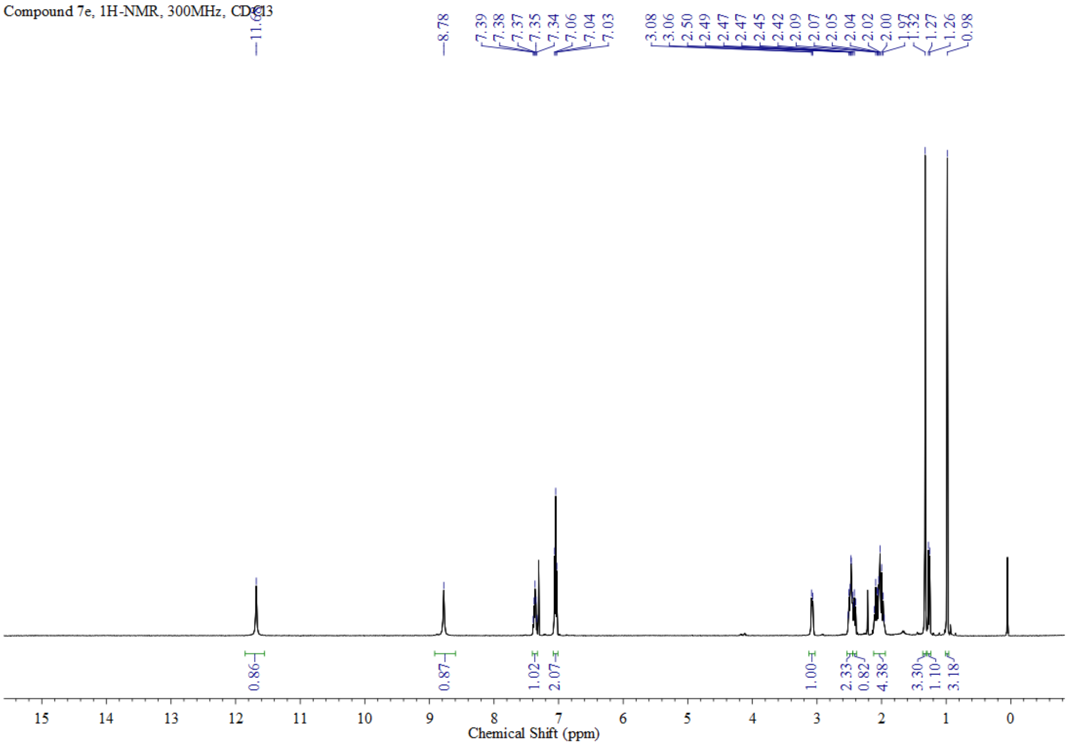

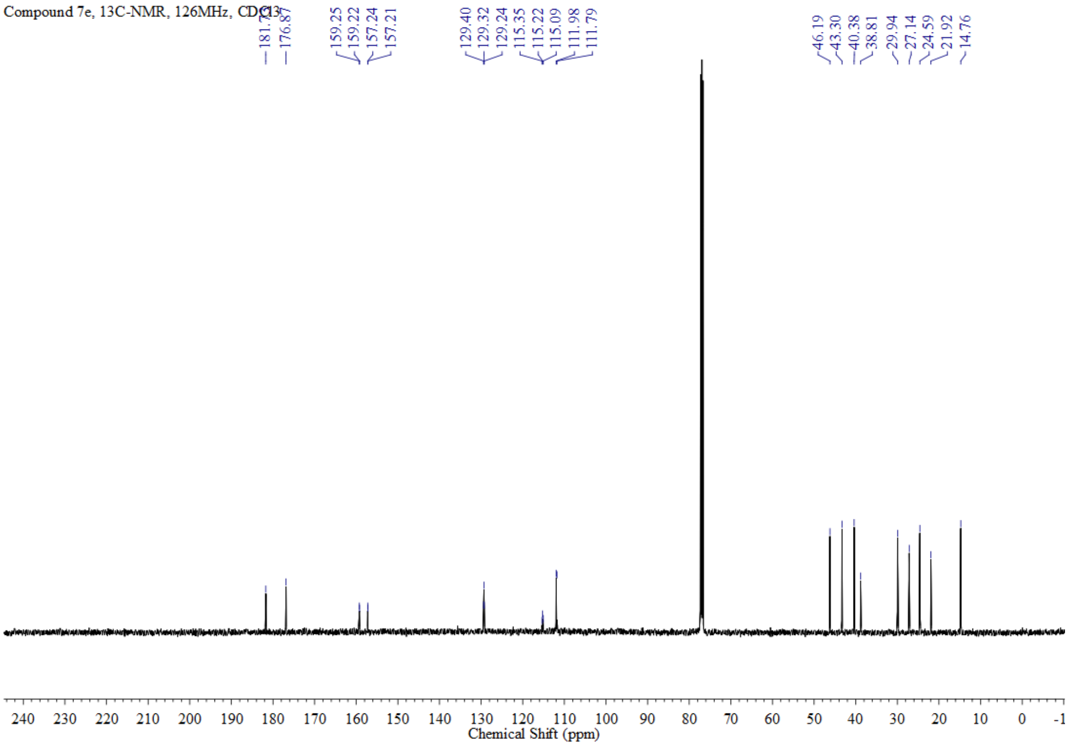

(1S,2S,5S)-N-((4-fluorophenyl)carbamothioyl)-6,6-dimethylbicyclo[3.1.1]heptane-2-carboxamide (4r)

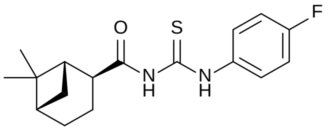

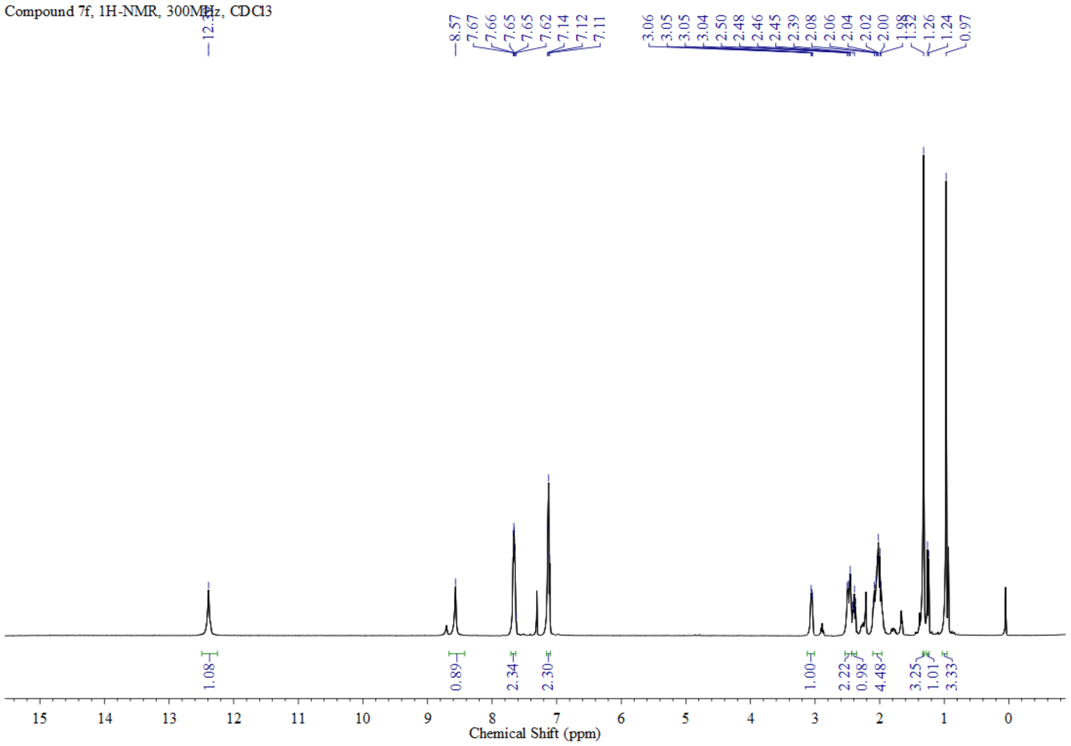

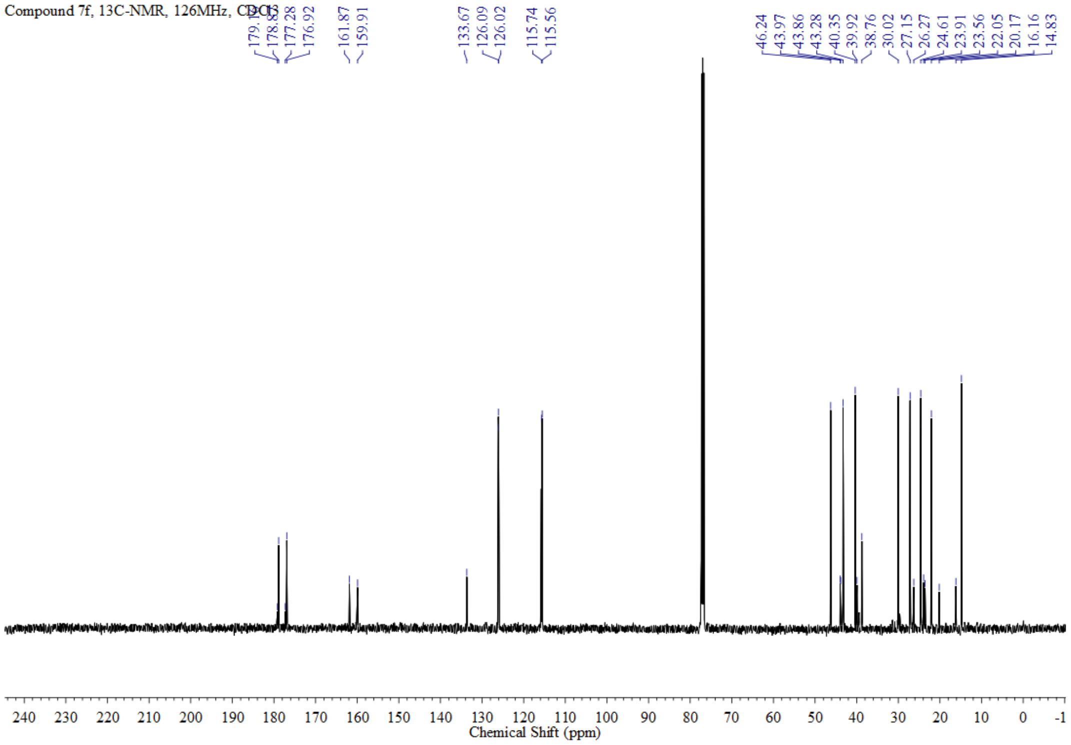

(1S,2S,5S)-N-((4-(4-fluorophenyl)thiazol-2-yl)carbamothioyl)-6,6-dimethylbicyclo[3.1.1]heptane-2-carboxamide (4s)

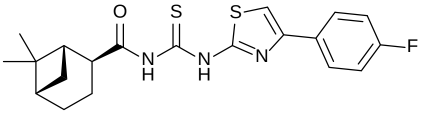

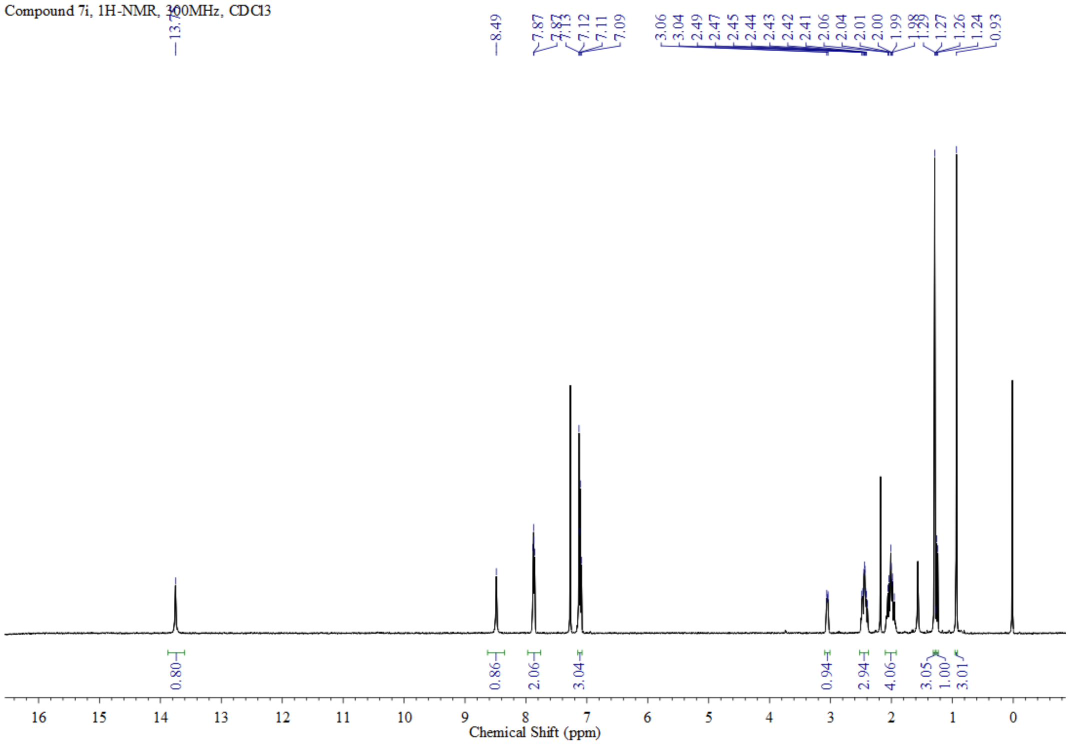

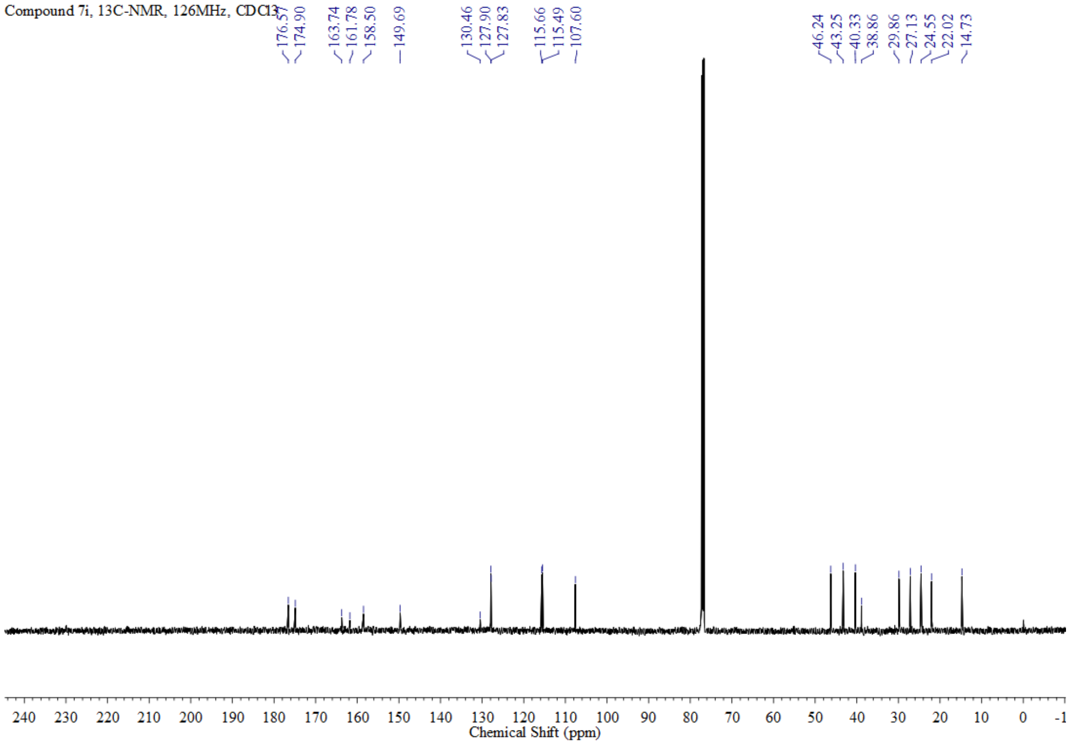

(1S,2S,5S)-6,6-dimethyl-N-((4-(4-nitrophenyl)thiazol-2-yl)carbamothioyl)bicyclo[3.1.1]heptane-2-carboxamide (4t)

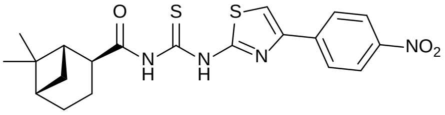

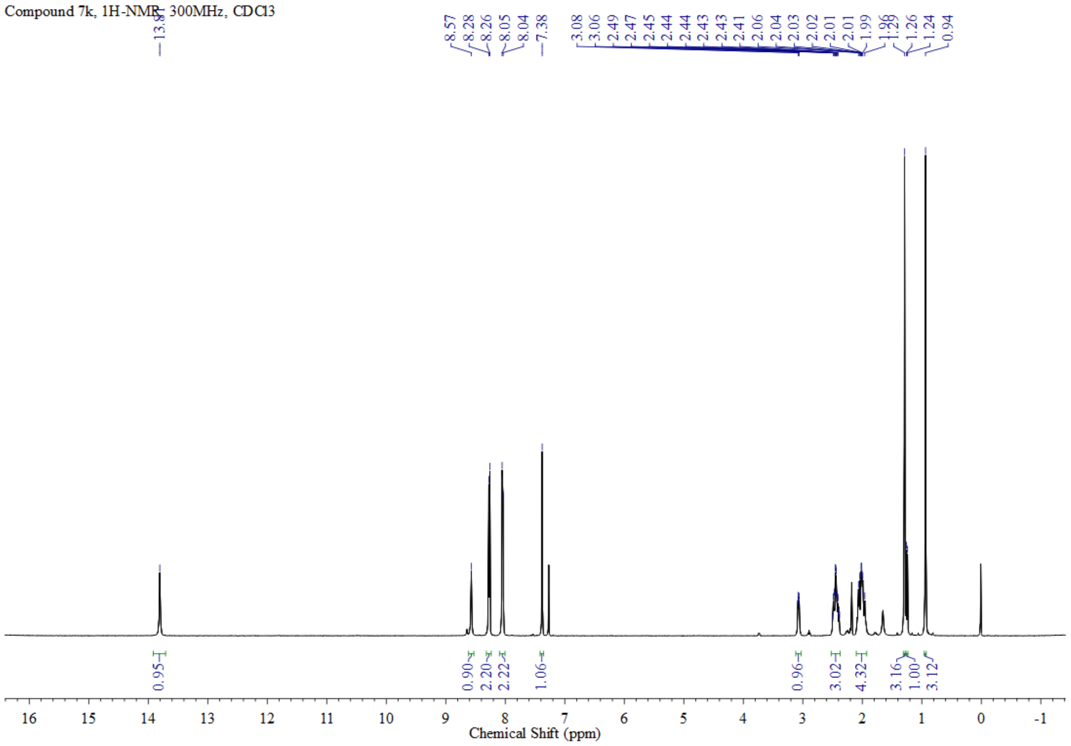

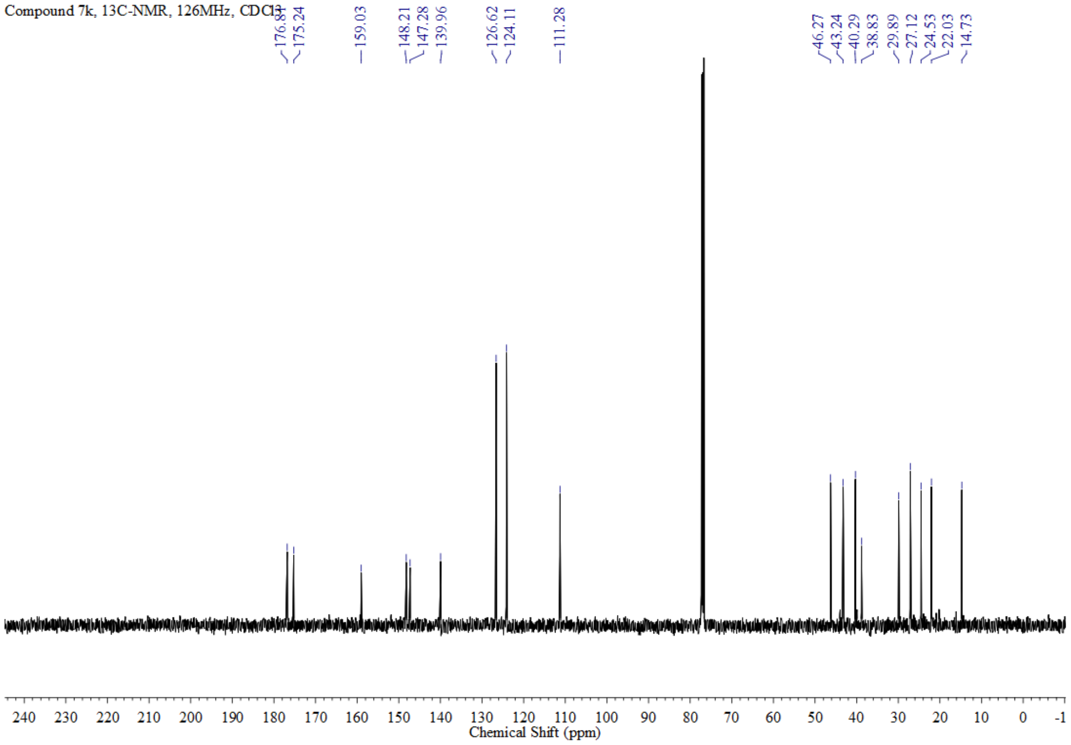

ESI-MS Spectra of 3 and 4a-4t

(1S,2S,5S)-6,6-dimethylbicyclo[3.1.1]heptane-2-carboxylic acid (3)

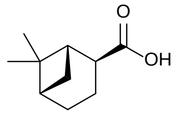

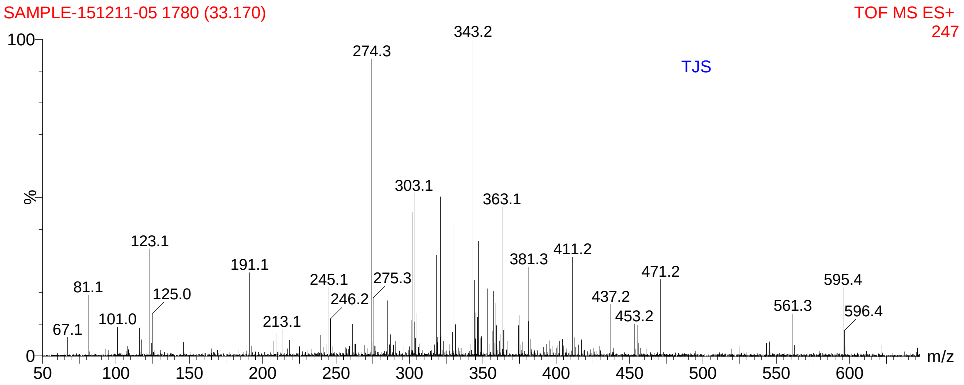

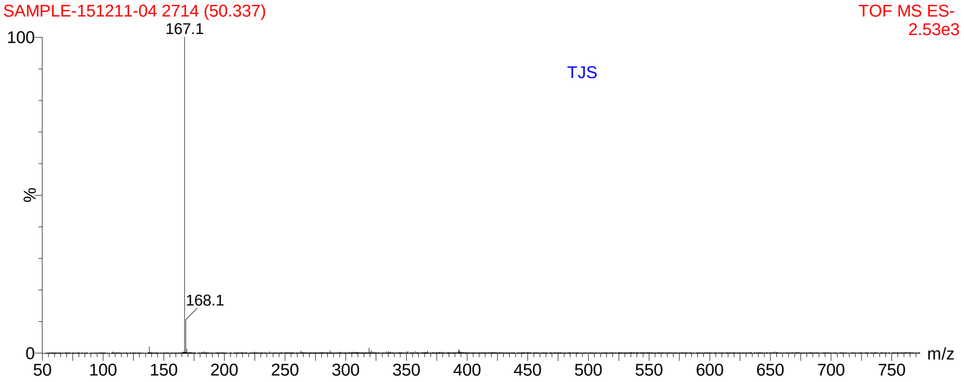

(1S,2S,5S)-6,6-dimethyl-N-phenylbicyclo[3.1.1]heptane-2-carboxamide (4a)

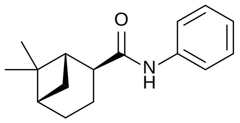

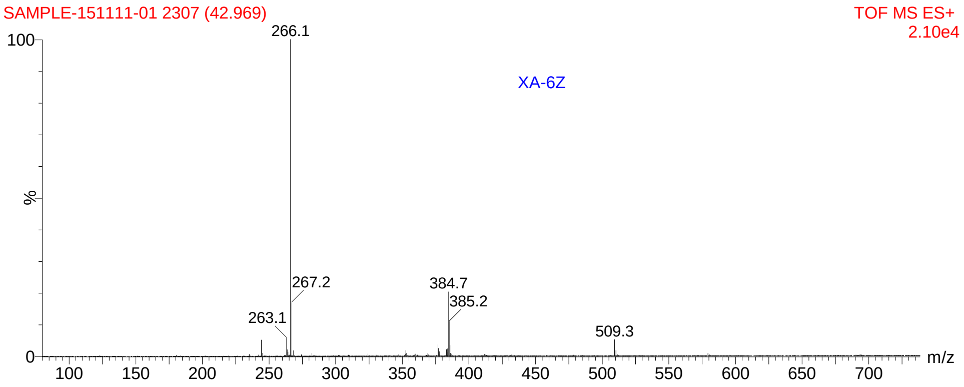

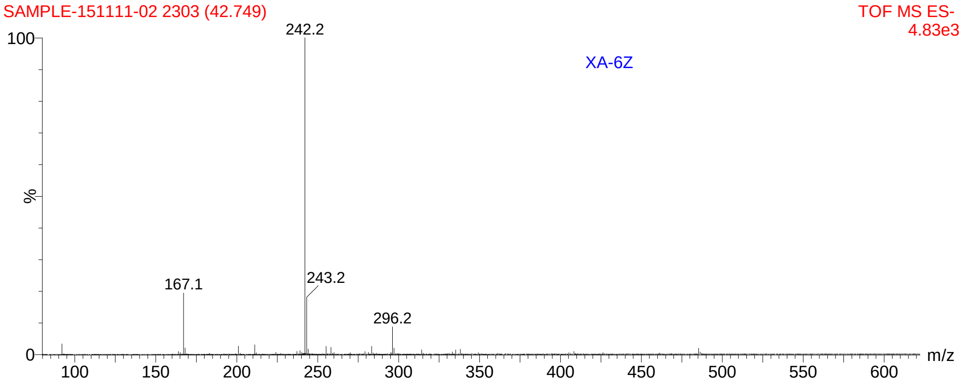

(1S,2S,5S)-N-(2-bromophenyl)-6,6-dimethylbicyclo[3.1.1]heptane-2-carboxamide (4b)

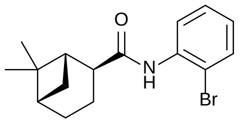

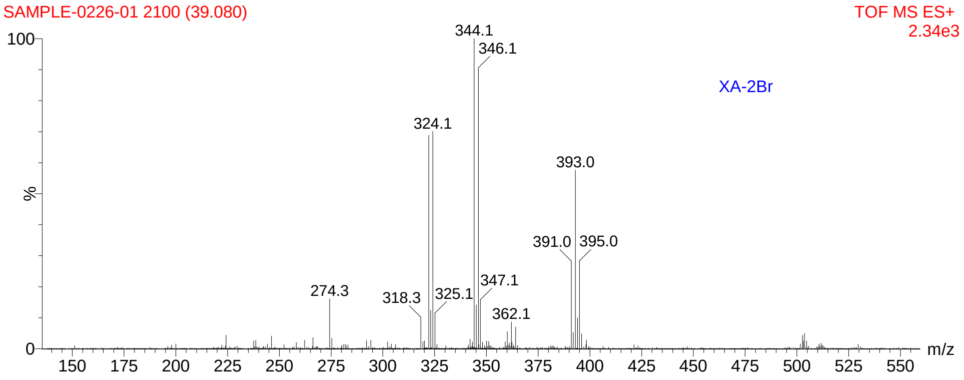

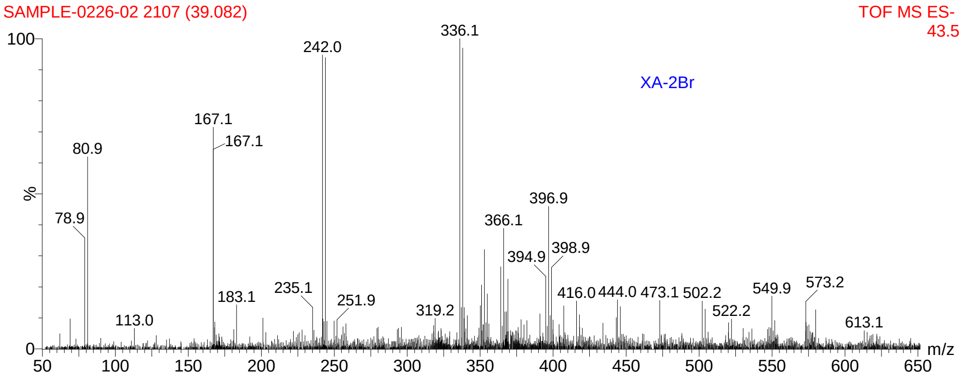

(1S,2S,5S)-N-(3-bromophenyl)-6,6-dimethylbicyclo[3.1.1]heptane-2-carboxamide (4c)

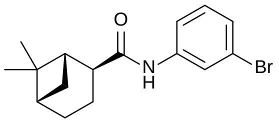

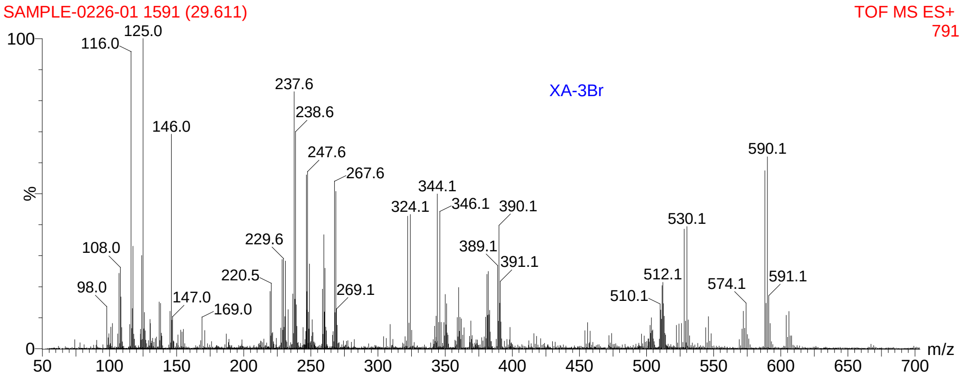

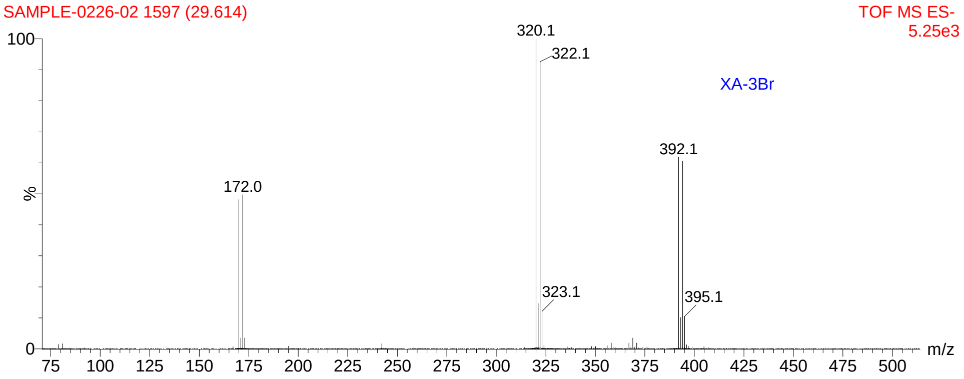

(1S,2S,5S)-N-(4-ethylphenyl)-6,6-dimethylbicyclo[3.1.1]heptane-2-carboxamide (4d)

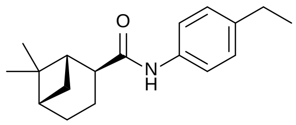

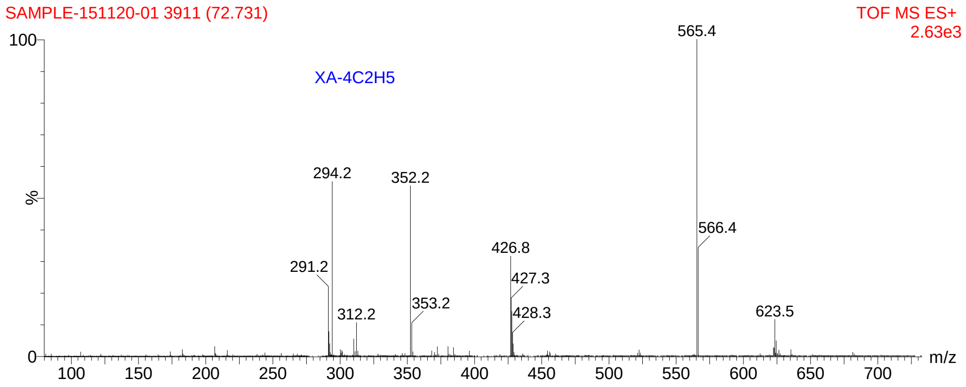

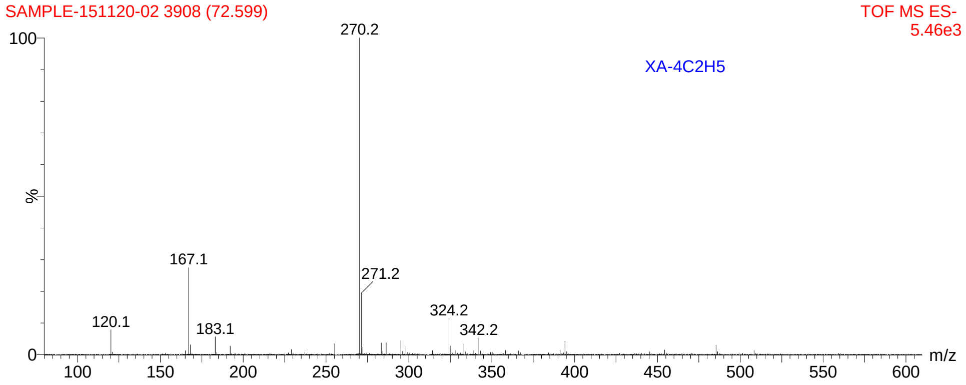

(1S,2S,5S)-6,6-dimethyl-N-(4-(trifluoromethyl)phenyl)bicyclo[3.1.1]heptane-2-carboxamide (4e)

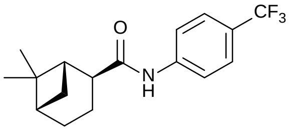

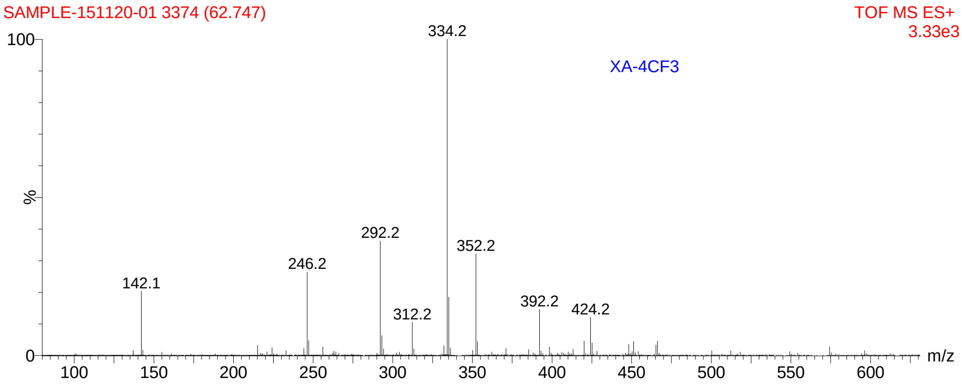

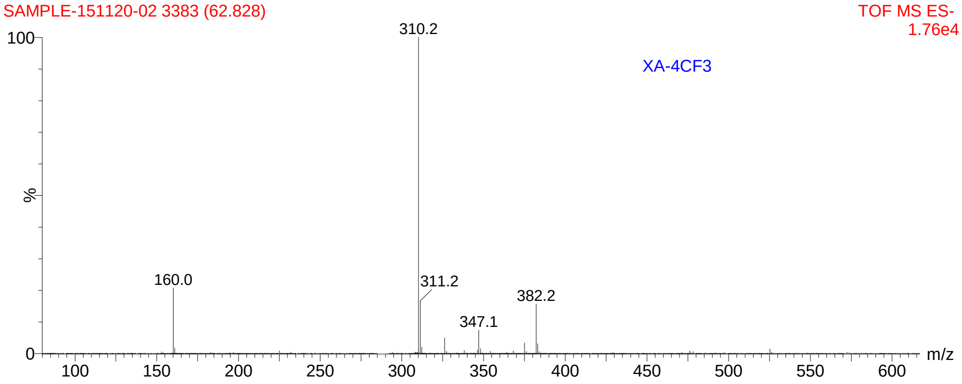

(1S,2S,5S)-N-(2,6-difluorophenyl)-6,6-dimethylbicyclo[3.1.1]heptane-2-carboxamide (4f)

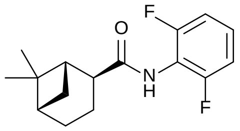

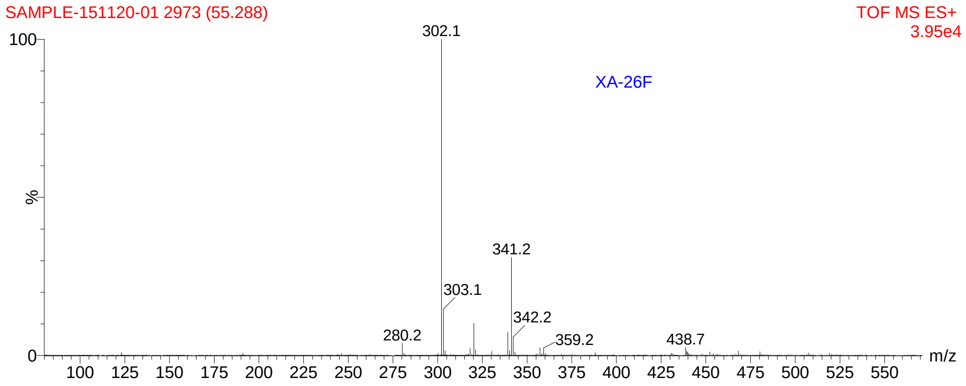

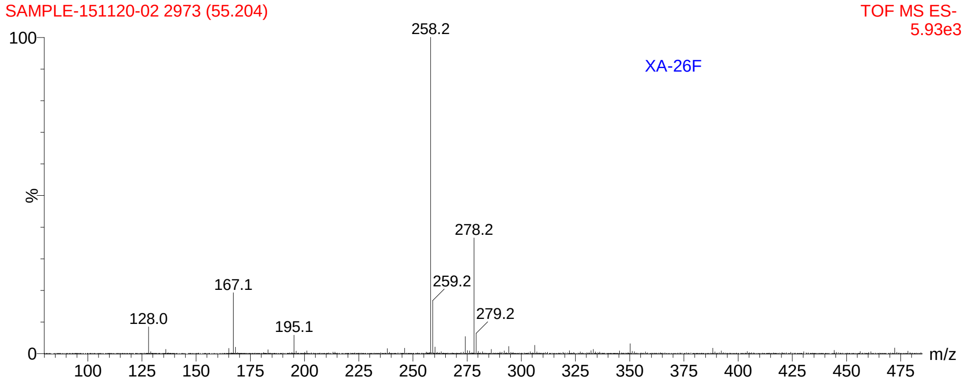

(1S,2S,5S)-N-(4-fluorophenyl)-6,6-dimethylbicyclo[3.1.1]heptane-2-carboxamide (4g)

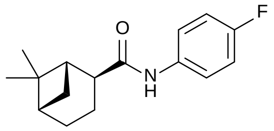

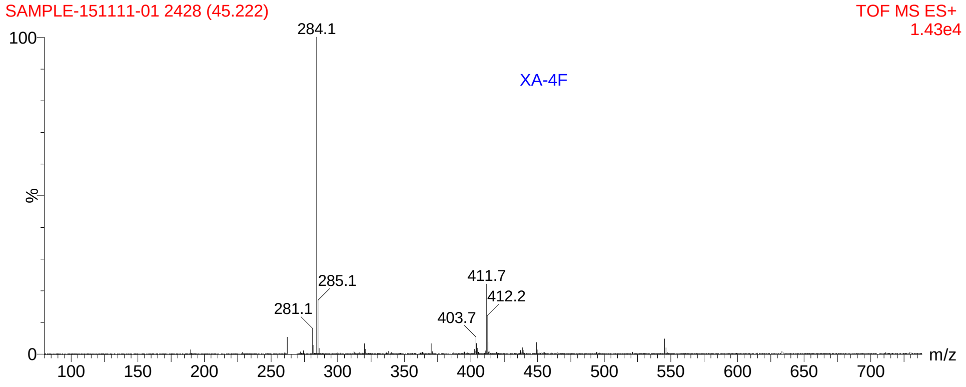

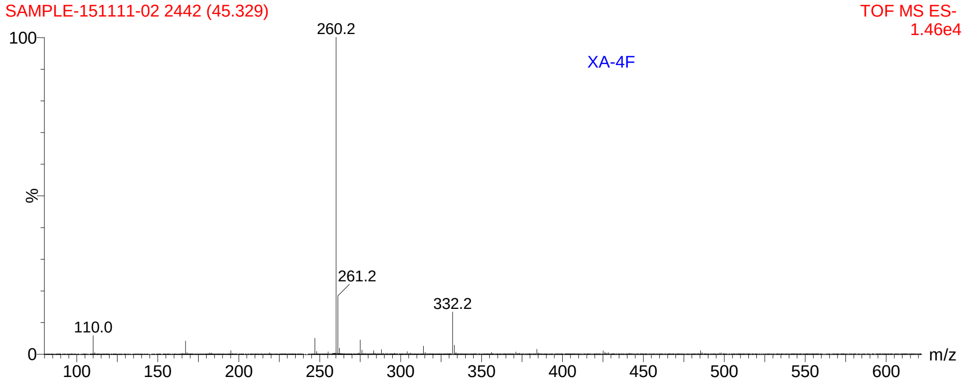

(1S,2S,5S)-6,6-dimethyl-N-(4-nitrophenyl)bicyclo[3.1.1]heptane-2-carboxamide (4h)

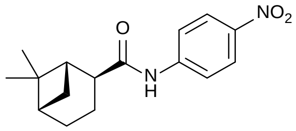

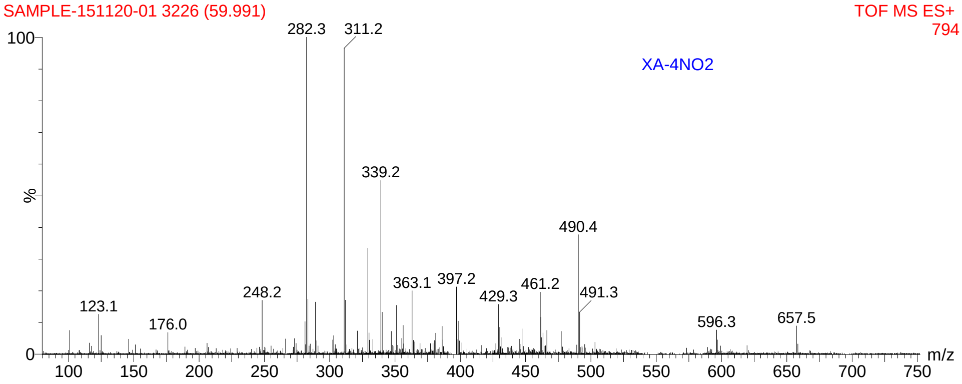

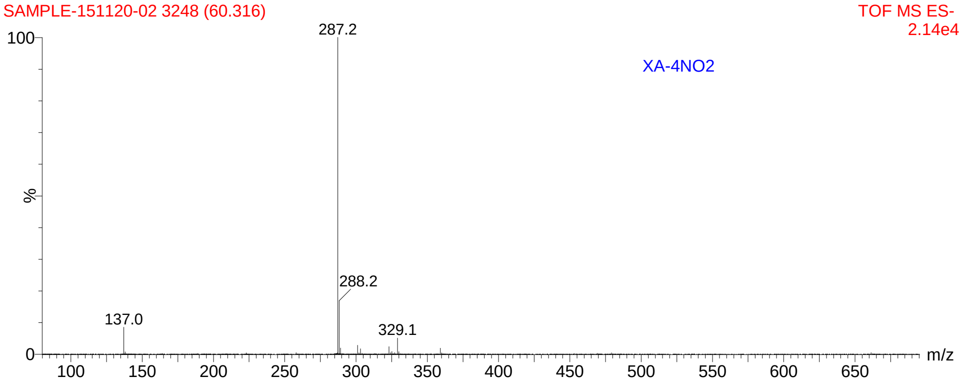

(1S,2S,5S)-6,6-dimethyl-N-(pyridin-2-yl)bicyclo[3.1.1]heptane-2-carboxamide (4i)

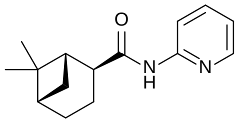

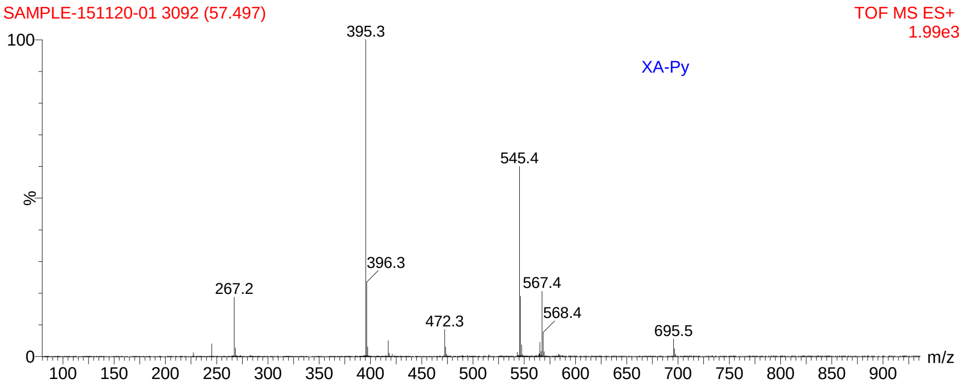

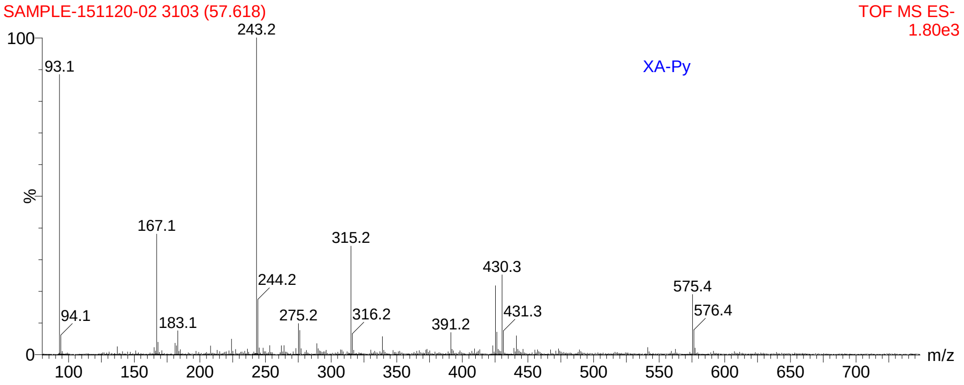

(1S,2S,5S)-N-benzyl-6,6-dimethylbicyclo[3.1.1]heptane-2-carboxamide (4j)

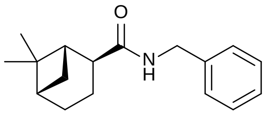

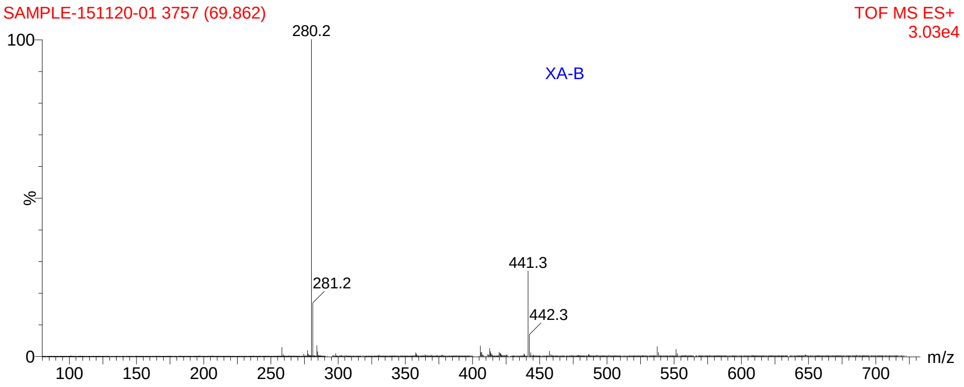

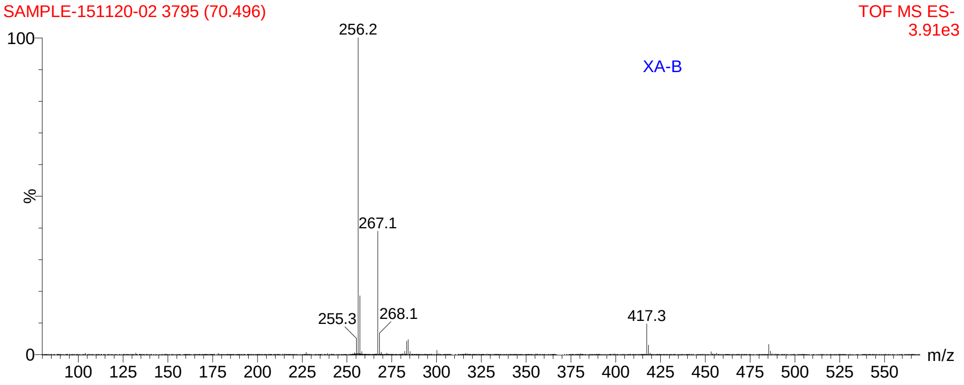

(1S,2S,5S)-N-(4-(4-fluorophenyl)thiazol-2-yl)-6,6-dimethylbicyclo[3.1.1]heptane-2-carboxamide (4k)

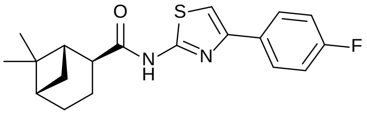

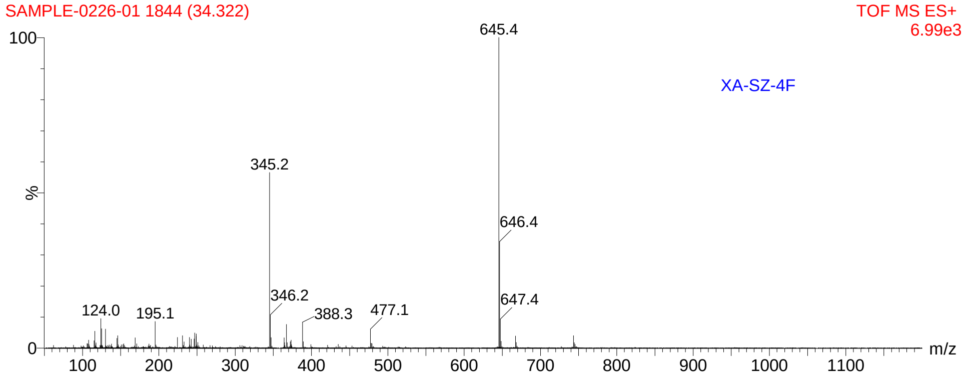

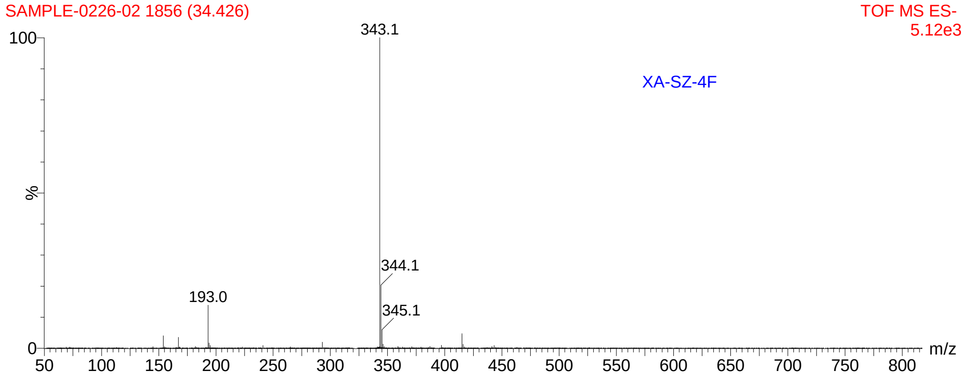

(1S,2S,5S)-N-(4-(4-methoxyphenyl)thiazol-2-yl)-6,6-dimethylbicyclo[3.1.1]heptane-2-carboxamide (4l)

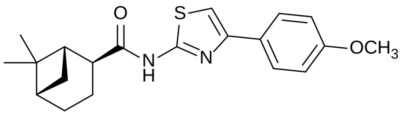

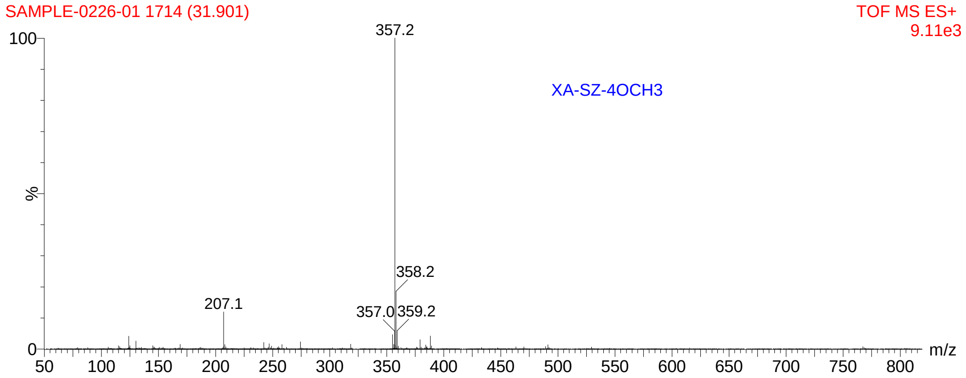

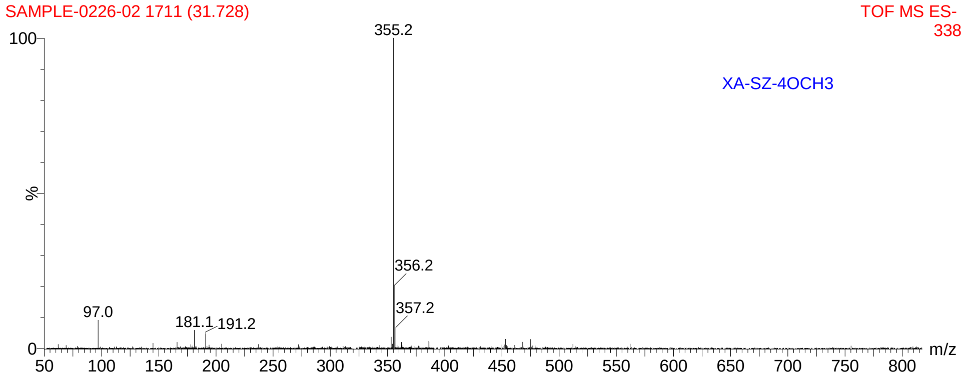

(1S,2S,5S)-6,6-dimethyl-N-(phenylcarbamothioyl)bicyclo[3.1.1]heptane-2-carboxamide (4m)

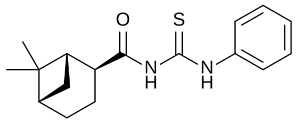

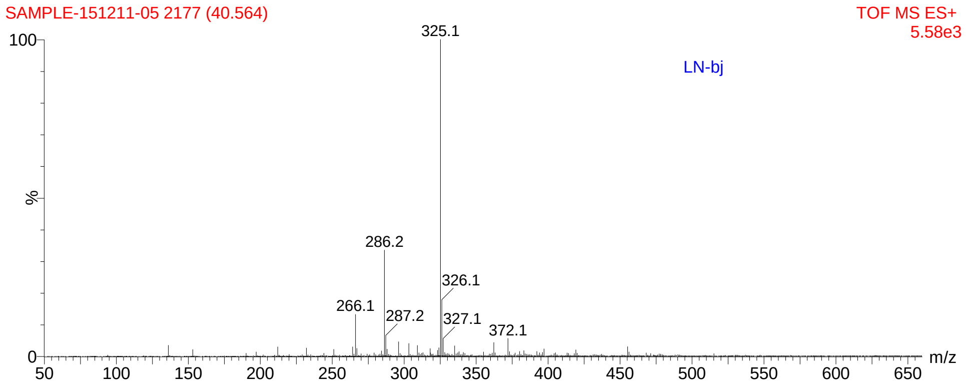

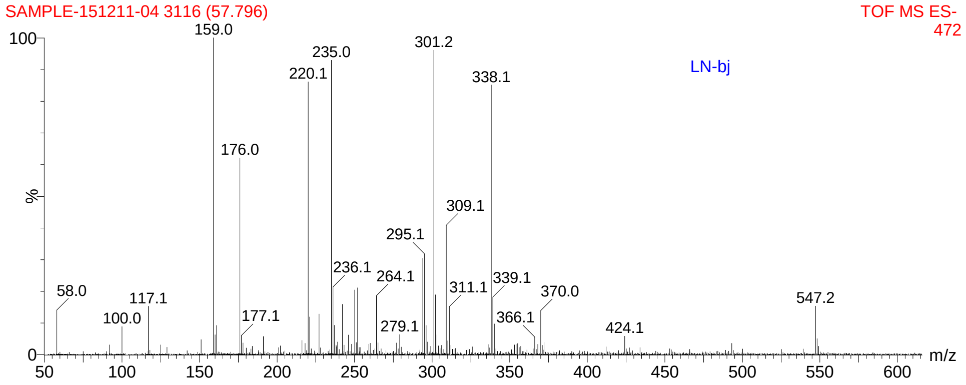

(1S,2S,5S)-N-((2-bromophenyl)carbamothioyl)-6,6-dimethylbicyclo[3.1.1]heptane-2-carboxamide

(4n)

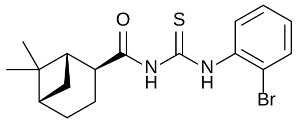

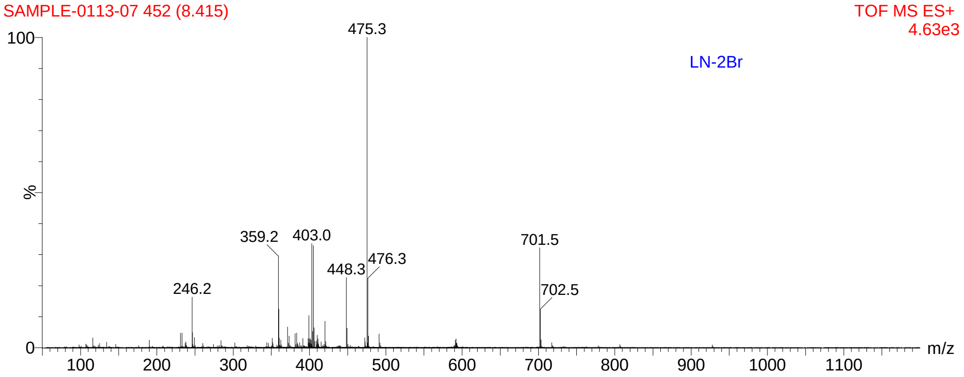

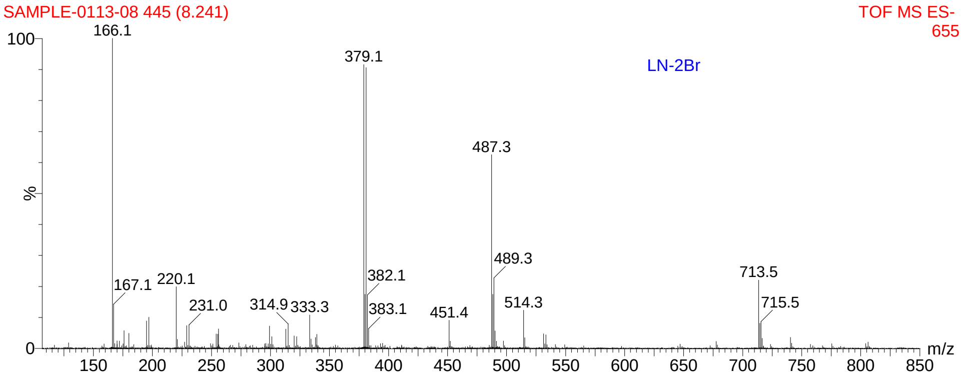

(1S,2S,5S)-N-((4-ethylphenyl)carbamothioyl)-6,6-dimethylbicyclo[3.1.1]heptane-2-carboxamide

(4o)

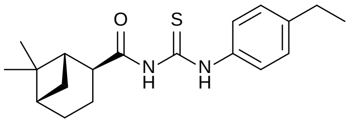

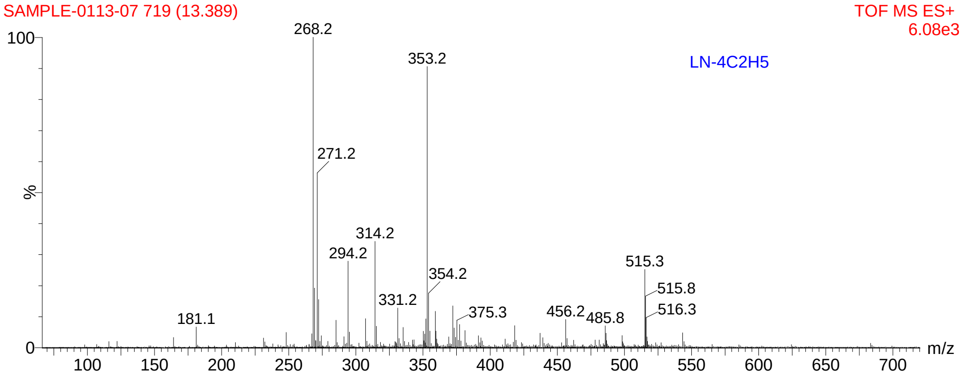

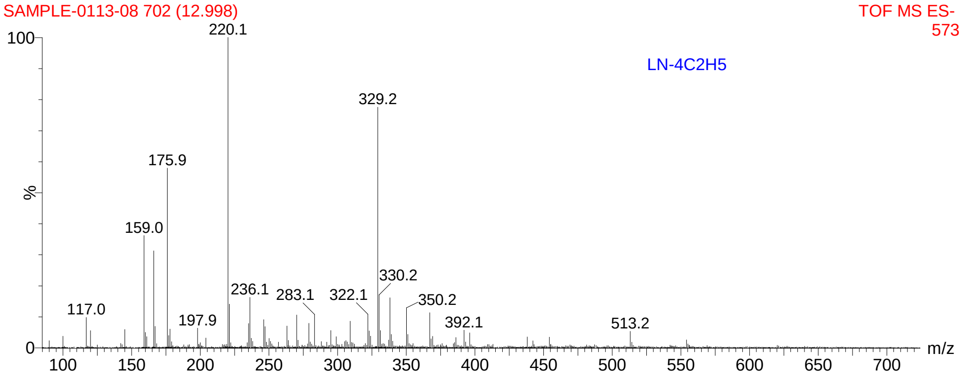

(1S,2S,5S)-6,6-dimethyl-N-((4-(trifluoromethyl)phenyl)carbamothioyl)bicyclo[3.1.1]heptane-2-carboxamide (4p)

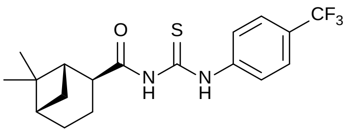

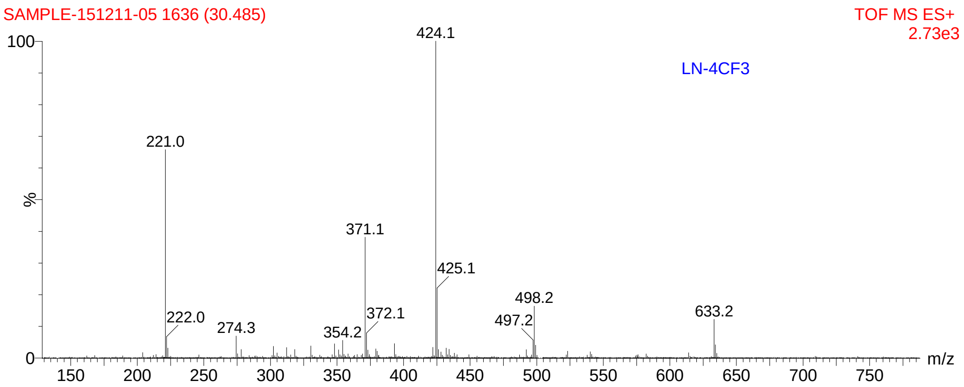

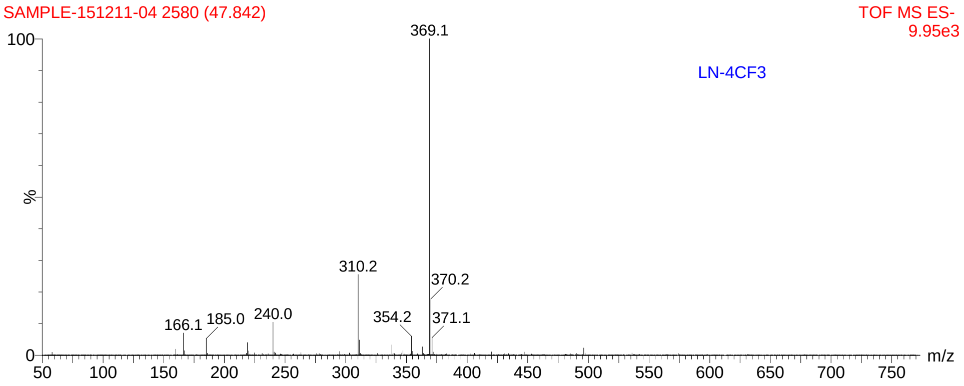

(1S,2S,5S)-N-((2,6-difluorophenyl)carbamothioyl)-6,6-dimethylbicyclo[3.1.1]heptane-2-carboxamide (4q)

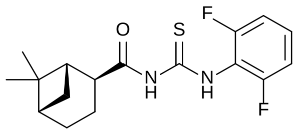

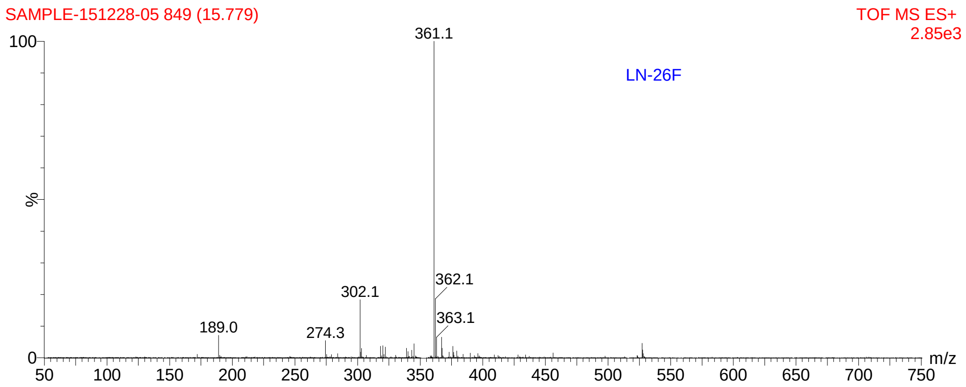

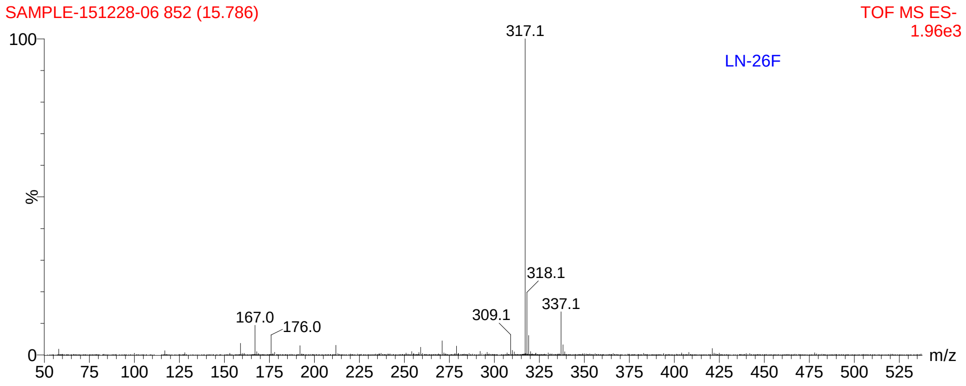

(1S,2S,5S)-N-((4-fluorophenyl)carbamothioyl)-6,6-dimethylbicyclo[3.1.1]heptane-2-carboxamide

(4r)

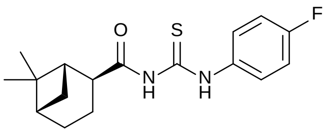

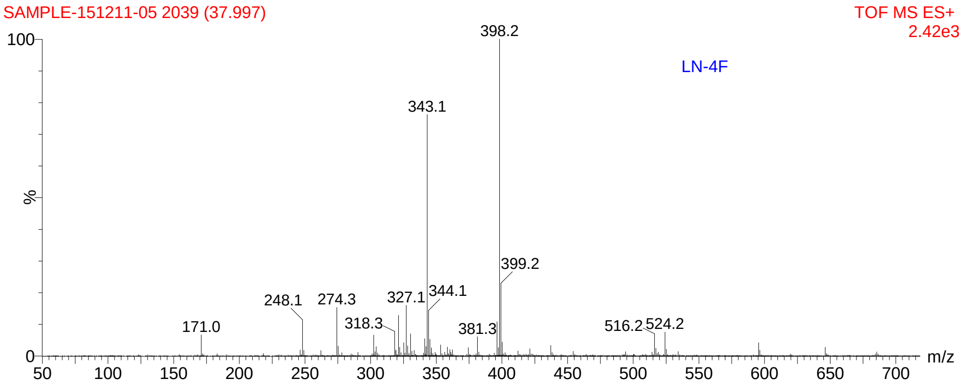

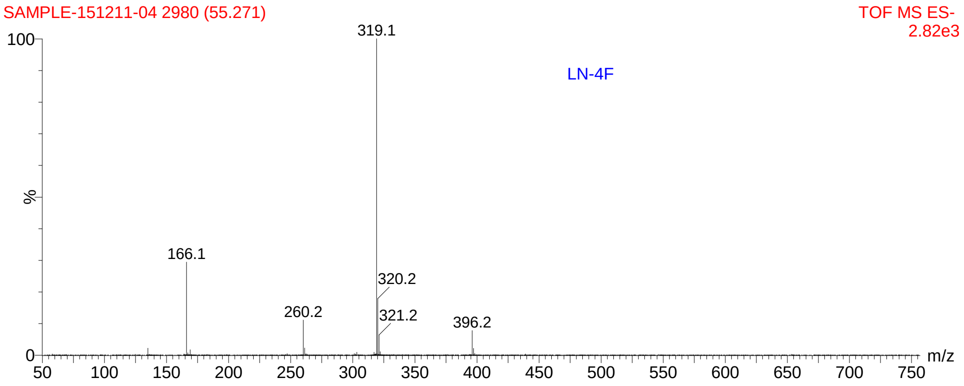

(1S,2S,5S)-N-((4-(4-fluorophenyl)thiazol-2-yl)carbamothioyl)-6,6-dimethylbicyclo[3.1.1]heptane-2-carboxamide (4s)

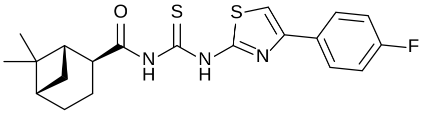

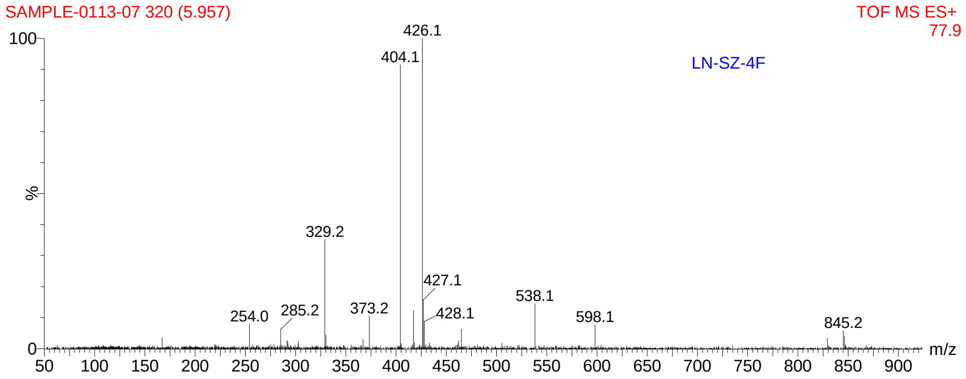

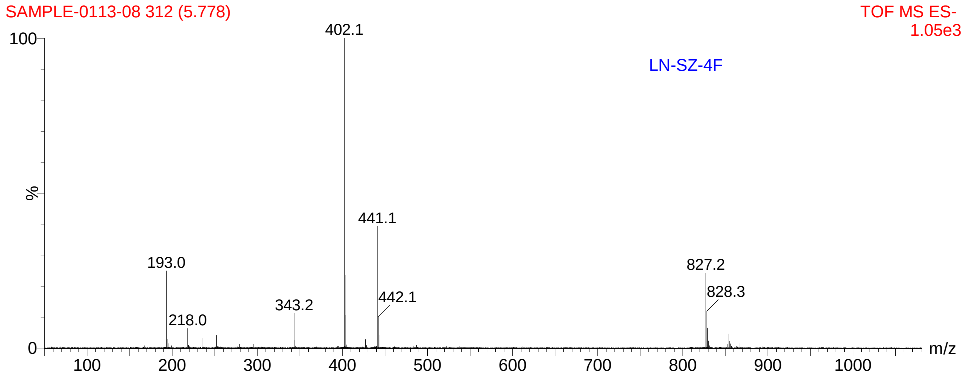

(1S,2S,5S)-6,6-dimethyl-N-((4-(4-nitrophenyl)thiazol-2-yl)carbamothioyl)bicyclo[3.1.1]heptane-2-carboxamide (4t)

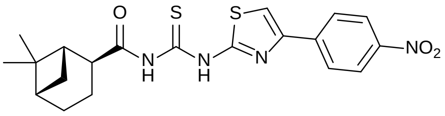

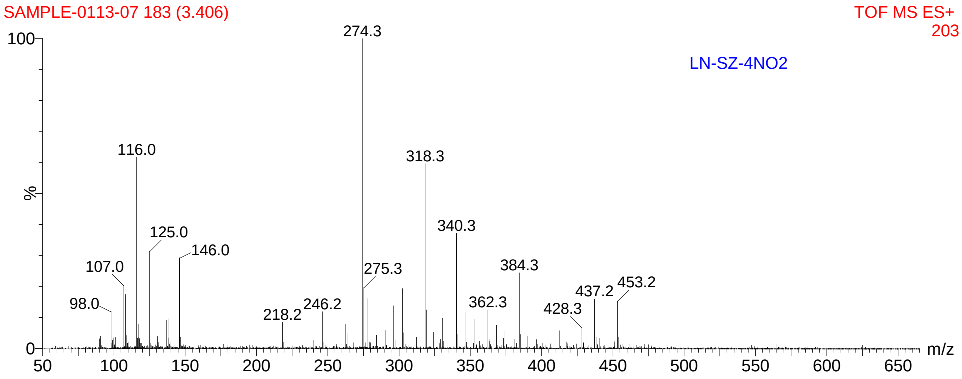

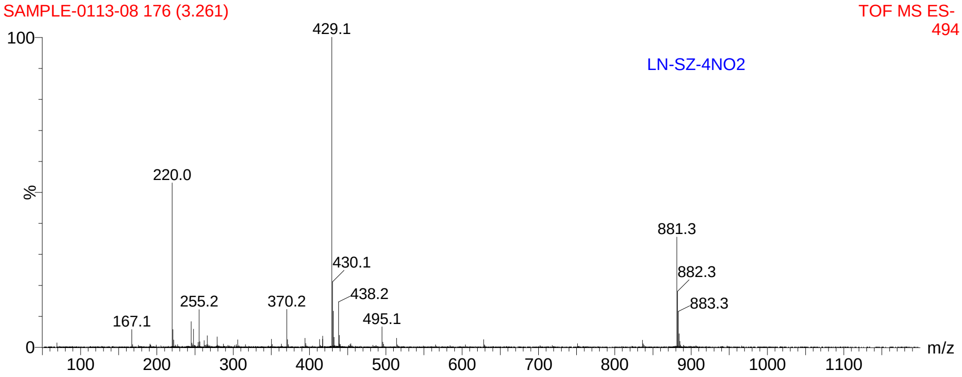

Supplement: Supplementary file 1 [file molecules-24-03144-s001.zip › molecules-567096-supplementary/Supplementary file 1.pdf]
