# Supplementary material for: Derivatization of Natural Compound β-Pinene Enhances Its In Vitro Antifungal Activity against Plant Pathogens
Source: Molecules. 2019 Aug 29;24(17):3144. doi: 10.3390/molecules24173144 (PMC6749435; doi:10.3390/molecules24173144)

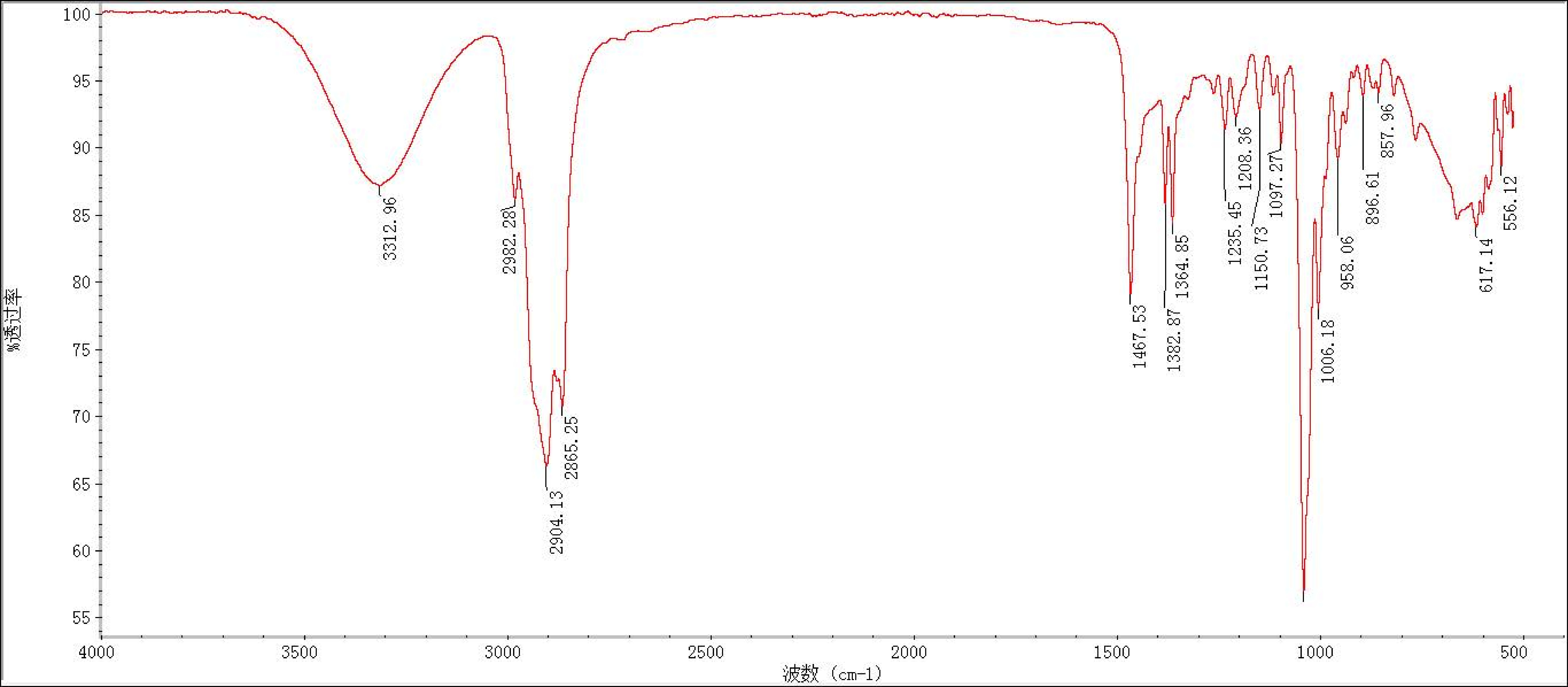

%透过率
波数 (cm-1)
100
95
90
85
80
75
70
65
60
55
4000
3500
3000
2500
2000
1500
1000
500
3312.96
2982.28
2904.13
2865.25
1467.53
1382.87
1364.85
1235.45
1208.36
1150.73
1097.27
1006.18
958.06
896.61
857.96
617.14
556.12

trans-myrtanol, 1H-NMR, 300MHz, CDCl3

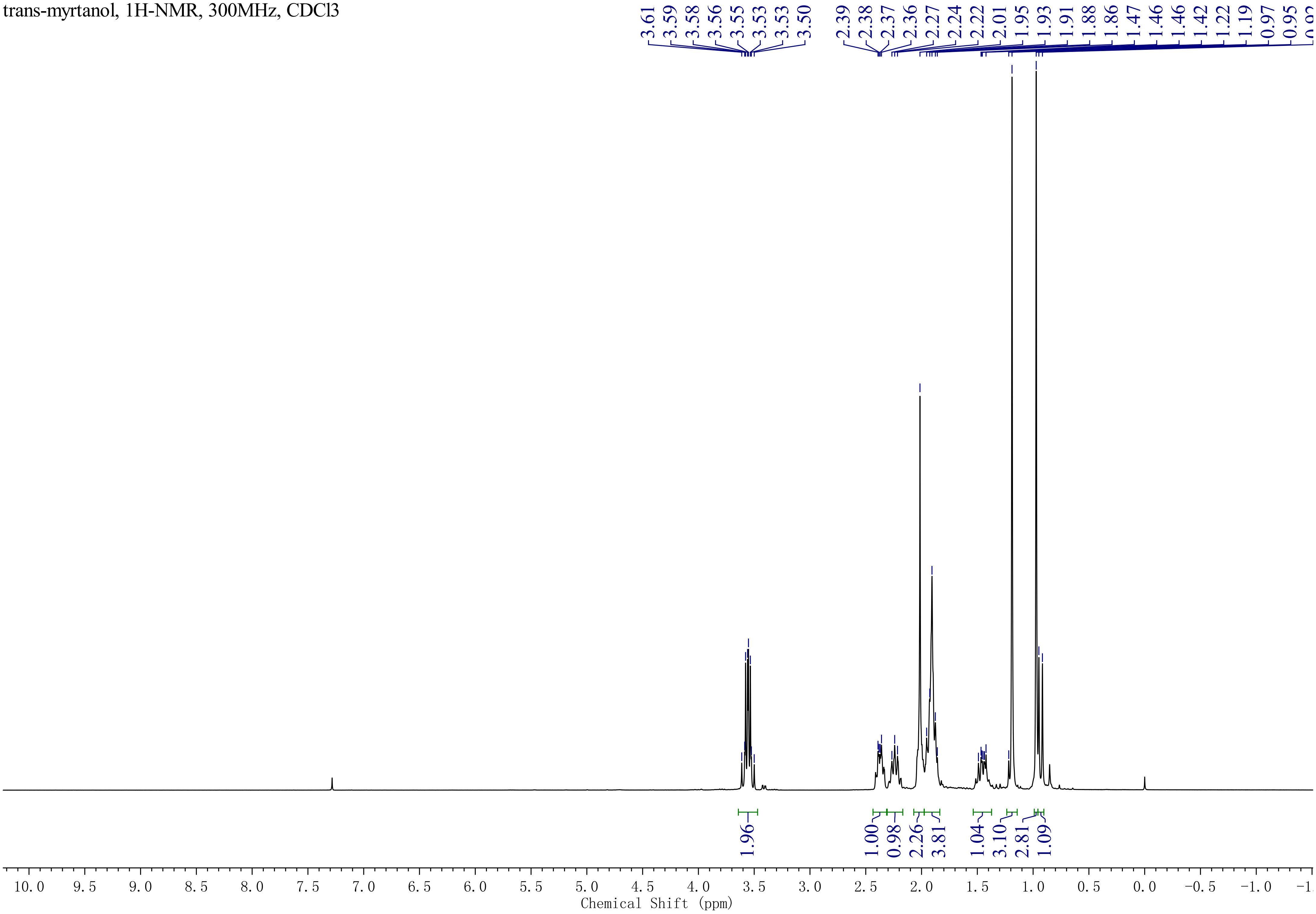

桃金娘烷醇, 13C-NMR, 75MHz,CDCl3

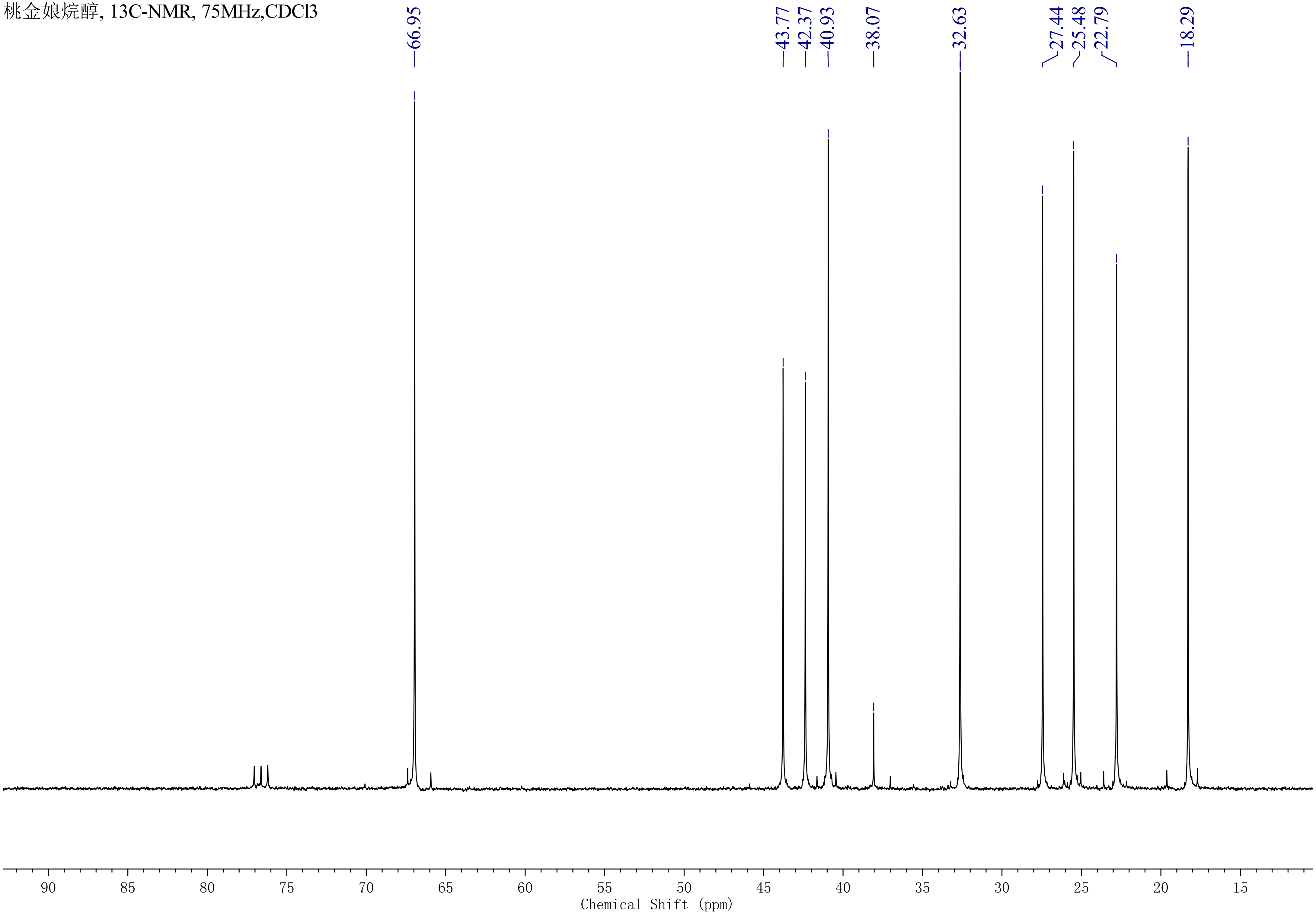

已检索谱库： C:\Database\NIST08.L
质量 ： 81
ID ： Bicyclo[3.1.1]heptane-2-methanol, 6,6-dimethyl-

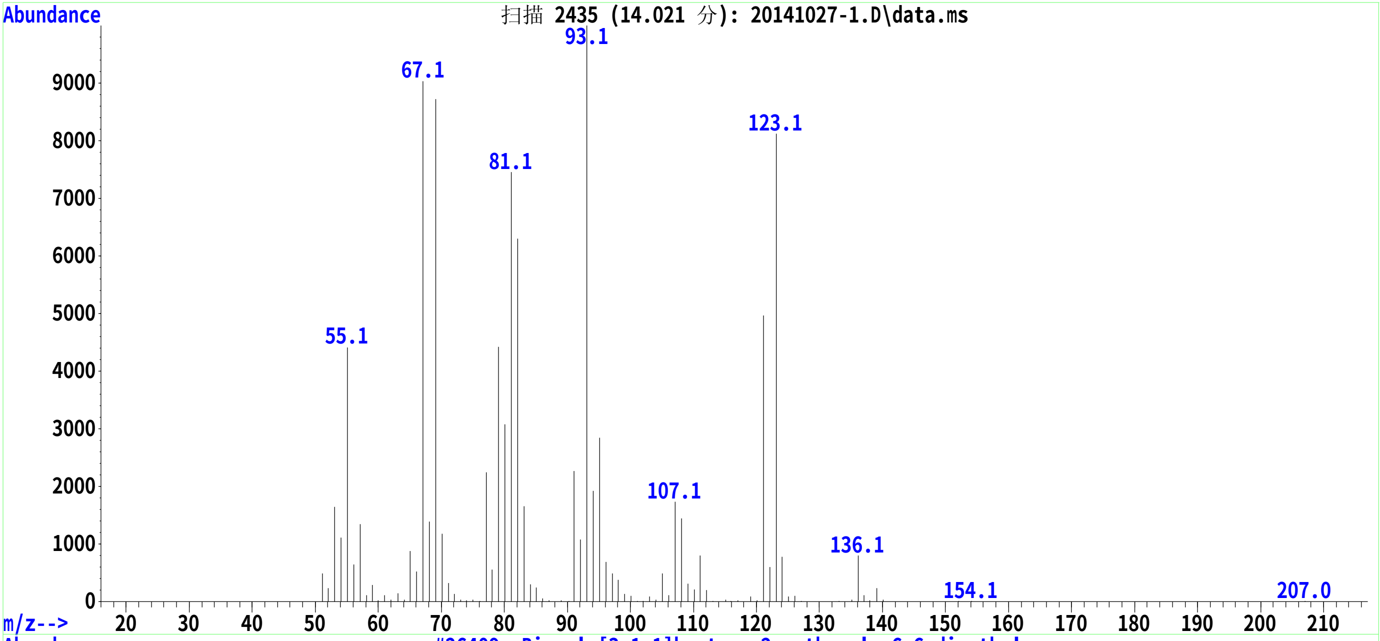

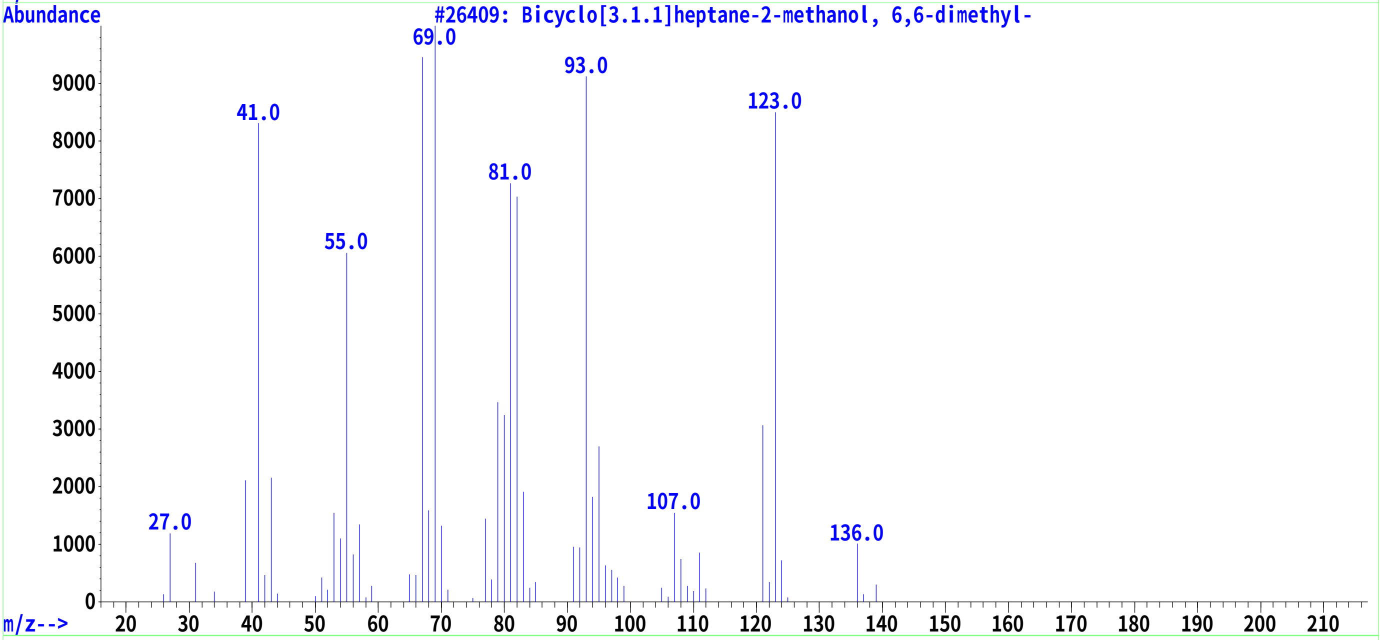

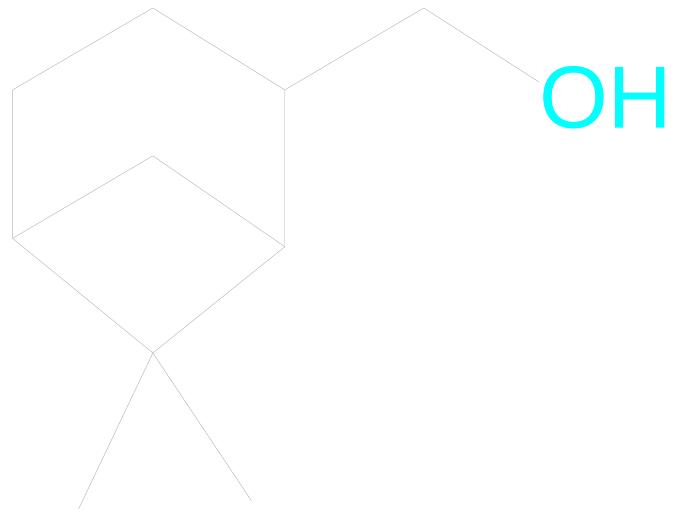

Supplement: Supplementary file 1 [file molecules-24-03144-s001.zip › molecules-567096-supplementary/Supplementary file 2-Spectra of myrtanol(IR+NMR+MS).pdf]
